# Genetic risk scores for coronary artery disease and its traditional risk factors: Their role in the progression of coronary artery calcification—Results of the Heinz Nixdorf Recall study

**DOI:** 10.1371/journal.pone.0232735

**Published:** 2020-05-07

**Authors:** Sonali Pechlivanis, Susanne Moebus, Nils Lehmann, Raimund Erbel, Amir A. Mahabadi, Per Hoffmann, Karl-Heinz Jöckel, Markus M. Nöthen, Hagen S. Bachmann, on behalf of the Heinz Nixdorf Recall Study Investigative Group

**Affiliations:** 1 Institute for Medical Informatics, Biometry and Epidemiology, University Hospital of Essen, University Duisburg-Essen, Essen, Germany; 2 Centre for Urban Epidemiology, University Hospital Essen, Essen, Germany; 3 Department of Cardiology and Vascular Medicine, West German Heart and Vascular Center, University Hospital Essen, Essen, Germany; 4 Department of Genomics, Life & Brain Center, University of Bonn, Bonn, Germany; 5 Division of Medical Genetics, Department of Biomedicine, University of Basel, Basel, Switzerland; 6 Institute of Pharmacology and Toxicology, Centre for Biomedical Education and Research, Witten/Herdecke University, Witten, Germany; Chuo University, JAPAN

## Abstract

**Background:**

Atherosclerosis is the primary cause of coronary artery disease (CAD). Several observational studies have examined the association of traditional CAD risk factors with the progression of coronary artery calcification (CAC). In our study we investigated the effect of 11 different genetic risk scores associated with CAD and CAD risk factors on the progression of CAC.

**Methods and results:**

We included 3097 participants from the Heinz Nixdorf Recall study who had available CAC measurements at baseline (CAC_b_) and at the 5-year follow-up (CAC_5y_). A weighted genetic risk score for CAD and each of the CAD-associated risk factors was constructed. Multiple regression analyses were applied to i) the difference between the observed log(CAC_5y_+1) (log(obs)) and expected log(CAC_5y_+1) (log(exp)) at the 5-year follow-up following the individual’s log(CAC_b_+1) percentile for the time between scans (log(obs)–log(exp)) and ii) the 5-year CAC progression, defined as 5*(log(CAC_5y_+1)–log(CAC_b_+1))/time between the scans, adjusted for age, sex, and log(CAC_b_+1) as well as for risk factors. The median percent deviation from the expected (CAC_5y_+1) and the 5-year progression of (CAC+1) in our study were 0 (first quartile: Q1; third quartile: Q3: -0.32; 0.48) and 45.4% (0%; 171.0%) respectively. In the age-, sex- and log(CAC_b_+1)-adjusted model, the per-standard deviation (SD) increase in CAD genetic risk score was associated with the percent deviation from the expected (CAC_5y_+1) (9.7% (95% confidence interval: 5.2%; 14.5%), p = 1.6x10^-5^) and the 5-year progression of CAC (7.1% (3.0%; 11.4%), p = 0.0005). The CAD genetic risk score explains an additional 0.6% of the observed phenotypic variance for “log(obs)–log(exp)” and 0.4% for 5-year progression of CAC. Additionally, the per-SD increase in the CAC genetic risk score was associated with the percent deviation from the expected (CAC_5y_+1) (6.2% (1.9%; 10.8%, p = 0.005)) explaining an additional 0.2% of the observed phenotypic variance. However, the per-SD increase in the CAC genetic risk score was not associated with the 5-year progression of CAC (4.4% (0.4%; 8.5%), p = 0.03) after multiple testing. Adjusting for risk factors did not change the results. None of the other genetic risk scores showed an association with the percent deviation from the expected (CAC_5y_+1) or with the 5-year progression of CAC.

**Conclusions:**

The association of the CAC genetic risk score and the CAD genetic risk score provides evidence that genetic determinants for CAC and CAD influence the progression of CAC.

## Introduction

Atherosclerosis is the primary cause of coronary artery disease (CAD) and precedes the onset of coronary heart disease (CHD) by decades. Atherosclerosis appears in the asymptomatic phase of CAD and can be detected as coronary artery calcification (CAC). Quantification of CAC allows better risk prediction of future cardiovascular disease (CVD) events [1–7]. It has been reported that the heritability of CAC progression is strong and accounts for approximately 40% of the observed variance, with 14% of the variation explained by genetic factors [8].

Several observational studies have examined the association of risk factors for CAD, including type 2 diabetes (T2D), circulating lipids, obesity, smoking and hypertension, with the progression of CAC [9–19]. However, different algorithms were used to model the progression of CAC in most of these studies. These studies modeled progression of CAC as: the annual CAC change, the annual CAC change on a log scale, the annualized relative rate of CAC change, mixed models or others. A recent study from our group used several published algorithms to model the progression of CAC for the risk prediction of coronary and CVD events [1].

The CAD-associated risk factors also have genetic determinants, which might influence the progression of CAC. Numerous large genome-wide association studies (GWAS) meta-analyses carried out on CAD as well as T2D, body mass index (BMI), low-density lipoprotein-cholesterol (LDL), high-density lipoprotein-cholesterol (HDL), triglycerides, total cholesterol (TC), systolic blood pressure (SBP), diastolic blood pressure (DBP) and pulse pressure (PP) have shown associations with several single nucleotide polymorphisms (SNPs) at the genome-wide significant level [20–32]. In addition, cross-sectional genetic studies on CAC have shown three SNPs to be associated with CAC [33–35].

In our study, we aimed to investigate the effect of genetic risk scores associated with CAD and each of the CAD risk factors (CAC, T2D, BMI, SBP, DBP, PP, LDL-cholesterol, HDL-cholesterol, triglycerides and TC) with progression of CAC.

## Materials and methods

Due to data security reasons i.e. the data contain potentially participant identifying information, the Heinz Nixdorf Recall Study does not allow sharing data as a public use file. However, for the purpose of replication, other authors/researchers are allowed to access data upon request, which is the same way the authors of the present paper obtained the data. Data requests can be addressed to: recall@uk-essen.de.

### Study population

At baseline (b), 4814 participants, aged 45 to 75 years (50% females), were randomly selected from the registration lists of the densely populated Ruhr metropolitan cities in Germany (residents of Essen, Bochum, and Mülheim) between December 2000 and August 2003. The rationale and design of the study were previously described in detail [36]. The participants were reinvited for the first follow-up examination, which took place 5 years after the baseline examinations. For this study, we excluded participants with prior CAD (coronary artery bypass surgery and/or interventional revascularization and history of prior myocardial infarction) (n = 327) at baseline. Of the remaining participants, we included only the participants with CAC measured during two time points i.e. at baseline (CAC_b_) and at 5-years (CAC_5y_) (approximately five years apart, 5.1+/-0.3 years) (n = 3675). We further excluded the participants: i) with stent implementation, bypass, balloon dilatation or myocardial infarction during 5-year follow-up (n = 154), ii) outside the study age range (45–74 at baseline, 50–79 at 5-year follow-up, n = 12) or iii) with missing Framingham risk factors information (n = 28) [1,10,17].

The Heinz Nixdorf Recall study participants were genotyped using Illumina GWAS chips (Illumina Omni1, OmniExpress, OmniExpress1, HumanCoreExome (v1.0 and v1.1) and the Metabochip [33,37]. The imputation of the study participants was carried out with IMPUTE v2.3.1 with reference data from 1000 Genomes Phase 1, release March 2012, for the Metabochip and 1000 Genomes Phase 3, release October 2014, for all the GWAS chip [38]. The imputed data were thereafter converted to the PLINK ped format using the threshold ≥ 0.8 in GTOOL v0.7.5. Thus, for all our analyses, we used 3097 participants with data on CAC_b_ and CAC_5y_ as well as the genotypes and were present both on the Metabochip and GWAS chips. The study was approved by the ethical committee at the University Hospital of Essen, Germany and was conducted in accordance with the principles expressed in the Declaration of Helsinki. The study was certified and recertified according to DIN EN ISO 9001:2000/2008. All study participants gave their written informed consent.

### Assessment of coronary artery calcification

CAC_b_ was assessed by a nonenhanced electron-beam scan with a C-100 or C-150 scanner (GE Imatron, San Francisco, CA, USA), as previously described [36]. The CAC_5y_ computer tomography (CT) was performed at the Radiology Department of the Alfred Krupp-Hospital, Essen with a C-150 scanner [17,39]. Prospective ECG triggering was performed at 80% of the RR interval, and contiguous 3 mm thick slices from the pulmonary bifurcation to the apex of the heart were obtained in both scans at an image acquisition time of 100 ms [10]. CAC was defined as a focus of at least 4 contiguous pixels with a CT density ≥130 Hounsfield units. The CAC score was determined using the methods of Agatston et al. [40]. The total CAC score was computed, comprising all calcified lesions in the coronary artery system. Analyses were performed using a Virtuoso workstation (Siemens Medical Solutions, Forchheim, Germany). CT scan results were not disclosed to the participants or to the study center.

A reassessment of CAC scoring, as previously reported, was implemented when extreme progression or regression from baseline to the 5-year examination was found (CAC_b_≤10 to CAC_5y_ >50, CAC_b_>20 to CAC_5y_ ≤10, or otherwise, >30% or <-7% annual change), accounting for the reproducibility by the given correction factors [10,41]. In two hundred forty-four cases, a reader with several years of experience in the evaluation of cardiac CT, who was blinded to the results of the initial reading as well as the risk factor profile of the participants, performed a second reading of the CAC score. At the end, the images of both CT examinations were re-evaluated offline using the same workstation (Aquarius, TerraRecon, Foster City, CA, USA) [10]. We further addressed the marked right-skewed distribution of CAC by using the log_e_ transformation of the CAC score plus 1, as previously suggested by Detrano et al. [42]

### Cardiovascular risk factors

BMI was calculated as weight divided by height squared (kg/m^2^). Medical history and smoking status (smokers (current or past) and non-smokers) were evaluated by computer-assisted interviews [39]. Current regular use of medication, including antihypertensive or lipid lowering drugs, was recorded in a standardized medication assessment. Resting blood pressure was measured with the participants seated, using an automated oscillometric blood pressure device (Omron, HEM-705CP-E). The mean of the second and third values of three measurements was calculated [43]. Standardized enzymatic methods were used to determine serum triglycerides, LDL-cholesterol and HDL-cholesterol values (ADVIA 1650, Siemens Medical Solutions, Erlangen, Germany) [1]. Diabetes was defined as meeting 1 of 4 criteria: (1) participants reported a history of clinically diagnosed diabetes, (2) participants took glucose-lowering drugs, (3) participants had fasting glucose levels of greater than 125 mg/dL, or (4) participants had nonfasting glucose levels of 200 mg/dL or greater. Socio economic status was defined by combining school and vocational training as total years of formal education according to the International Standard Classification of Education (UNESCO 1997) and categorized into two groups (≤13, ≥14 years).

### Genetic risk scores

We tested 11 genetic risk scores based on the known GWAS (p≤5x10^-8^) SNPs associated with CAD [22,26,28,32], T2D [21,23,29,31], BMI [30], BP (SBP, DBP and PP) [25,27], lipids (LDL-cholesterol, HDL-cholesterol, triglycerides and TC) [20,24] and a combined set of three CAC SNPs selected from the studies by O'Donnell CJ et al., van Setten et al. and Pechlivanis et al. [35,33,34]. If two SNPs were in high linkage disequilibrium (LD) (D’ = 1 and R2≥0.80) then only one of the two SNPs was included in the genetic risk score. The LD between the SNPs used in each of the genetic risk scores was calculated using the Ldlink software and R2 between the SNPs are presented in the (S7A Table–S7K Table) [44]. The average weighted genetic risk score for each individual was constructed by multiplying the risk estimate (odds ratio transformed by the natural log for CAD, T2D and the beta estimate for CAC, BMI, BP and lipids SNPs) with the number of risk alleles (0 (no risk allele), 1 (1 risk allele), 2 (2 risk alleles)) of each trait associated SNP. The products were then summed and divided by the number of SNPs used for each trait. If the genotype in the score for a particular individual was missing, then the expected value was imputed based on the sample allele frequency. The allelic scoring routine in PLINK was used to calculate the genetic risk score [45]. The mean and standard deviation (SD) of the study population were used to standardize each genetic risk score to have a mean of zero and unit variance. Genetic risk was then analyzed per-SD of the standardized genetic risk scores for each of the traits.

### Statistical methods

#### Progression of CAC as a continuous outcome

Currently no established mathematical model is accepted as a gold standard for the calculation of CAC progression. In our study, we used two published algorithms to model CAC progression as continuous outcomes. We modeled the continuous outcomes as

- We verified in [1] that individual CAC_5y_ at the 2^nd^ visit is to a good degree approximated by following the individual‘s baseline CAC percentile with age for the time between scans, which yields expected CAC_5y_. The difference between logarithmized observed log(CAC_5y_+1) and this expected log(CAC_5y_+1) at 2nd visit, “log(obs)–log(exp)”, is our first endpoint. This difference between the observed and expected log(CAC_5y_+1) indicates an accelerated increase or decrease of CAC in the 5-year period compared to what was expected from the baseline CAC percentile value. In other words, the higher the deviation, the greater the progression when compared with the expected log(CAC_5y_+1).
  and
- The 5-year progression of CAC (progression of CAC) is defined as the observed 5-year CAC minus baseline CAC, normalized to a 5-year interval: 5*(log(CAC_5y_+1)–log(CAC_b_+1))/T, where T denotes the individual follow-up time (5.1±0.3 years) [10]. Here, we normalized the progression of CAC on the log-scale to a common 5-year difference in time between measurements.

We applied linear regression to study the relationship between the genetic risk scores and the continuous outcomes on log-scale to estimate the effect size and 95% confidence interval (95% CI). The effect size estimates and 95% CIs obtained were transformed to the original scale and presented as the percent deviation from the expected (CAC_5y_+1) for the outcome “log(obs)–log(exp)” [1] and the percent change in (CAC+1) for the outcome “progression of CAC” [10]. The residual distribution plots for the percent deviation from expected (CAC_5y_+1) and the progression of CAC showed a spike at “0” but were acceptably normal. We did not observe a skewed or nonlinear relationship between the outcomes and any of the genetic risk scores. Furthermore, we did not detect multicollinearity between the variables used in the adjusted models.

We controlled for multiple testing at 5% for our primary question relating the association of genetic risk scores with the progression of CAC in the age-, sex- and log(CAC_b_+1)-adjusted model. Consequently, we corrected for 11 statistical tests, which corresponds to a α_BF_ = 0.005 using the Bonferroni procedure.

For sensitivity analyses, we used the information on the family history of CHD, defined as fatal or nonfatal CHD or sudden cardiac death in a family that occurred before age 55 in the case of father/brother and before age 65 in the case of mother/sister. The data on family history of CHD were available for 2845 (91.9%) participants. Participants who did not know whether their parents had any CHD or who did not know their biological parents (n = 252 (8.1%)) were excluded from the sensitivity analyses.

The continuous data are presented as the mean±SD or median (first quartile: Q1, third quartile: Q3) if the distributions of the data were substantially skewed. The count data are presented as frequencies and percentages. All the statistical analyses were performed using Plink v.19 (https://www.cog-genomics.org/plink2) [45] and SAS v.9.4 (SAS Institute, Cary, North Carolina, USA).

## Results

### Characteristics of the study population

The baseline characteristics of the 3097 Heinz Nixdorf participants in our study are presented in Table 1. During the median follow-up time of 5.1 years, the median percent deviation from the expected (CAC_5y_+1) and 5-year increase in (CAC+1) were 0 (Q1; Q3: -0.32; 0.48) and 45.4% (0%; 171.0%) respectively. The mean±SD for each genetic risk score is listed in Table 1.

**Table 1:** Basic characteristics of the study population.

	N = 3097
Age (years) *	58.9±7.5
Women	1630 (52.6)
Body mass index (kg/m^2^) *	27.6±4.3
Diabetes	348 (11.2)
Diastolic blood pressure (mmHg) *	81.5±10.6
Systolic blood pressure (mmHg) *	132.0±20.0
Hypertension	1630 (52.6)
Antihypertension medication	941 (30.4)
LDL-cholesterol (mg/dL) *	146.1±36.0
HDL-cholesterol (mg/dL) *	59.5±17.3
Triglycerides (mg/dL)) †	122.0 (88.0; 175.0)
Total cholesterol *	231.22±38.5
Lipid-lowering medication	275 (9.5)
Smoking	1754 (56.2)
SES	1049 (33.9)
CAC at baseline †	7.2 (0.0; 91.8)
Log (obs)–log (exp) (median % (Q1, Q3))	0 (-0.32; 0.48)
5-year increase in (CAC+1) (median % (Q1, Q3))	45.4 (0; 171.0)
Coronary artery disease GRS *	0.037±0.003
CAC GRS *	0.107±0.058
Type 2 diabetes GRS *	0.049±0.005
Body mass index GRS *	0.012±0.0009
Systolic blood pressure GRS *	0.272±0.021
Diastolic blood pressure GRS *	0.144±0.011
Pulse pressure GRS *	0.157±0.012
LDL-cholesterol GRS *	0.025±0.002
HDL-cholesterol GRS *	0.022±0.002
Triglyceride GRS *	0.025±0.003
Total cholesterol GRS *	0.027±0.002

LDL: low-density lipoprotein, HDL: high-density lipoprotein, SES: socio economic status, CAC: coronary artery calcification, GRS: genetic risk score. Data are given as number (percentage) unless otherwise indicated.

* Data are given as the mean±SD.

^†^ Data are given as the median (Q1; Q3).

### Association of genetic risk score with the continuous measure of the progression of coronary artery calcification

In linear regression adjusting for age, sex and log(CAC_b_+1), a significant association was observed between the CAD genetic risk score and “log(obs)–log(exp)”, even after adjusting for multiple testing. A per-SD increase in the CAD genetic risk score increased the deviation from the expected (CAC_5y_+1) by 9.7% ((5.2%; 14.5%), p = 1.6x10^-5^), indicating an accelerated increase of CAC in the 5-year period compared with what was expected from the baseline CAC percentile value. Furthermore, Table 2 (Model 1) showed that age, sex and log(CAC_b_+1) contributed significantly to the outcome “log(obs)–log(exp)”. A similar effect was observed in the analysis using the progression of CAC as the outcome and remained significant even after multiple testing. With a per-SD increase in the CAD genetic risk score, the progression of CAC was increased by 7.1% ((3.0%; 11.4%), p = 0.0005) (Table 3). In Model 1 (Table 3), age, sex and log(CAC_b_+1) contributed significantly to the progression of CAC. The CAD genetic risk score explains an additional 0.6% of the observed variance for “log(obs)–log(exp)” compared to the base model adjusted only for age, sex and log(CAC_b_+1) (R^2^ = 3.2%, data not shown) (Table 2). Similarly, the explained phenotypic variance by the CAD genetic risk score for the outcome progression of CAC was 0.4% when compared to the base model adjusted for age, sex and log(CAC_b_+1) (R^2^ = 2.8%, data not shown) (Table 3).

**Table 2:** Estimated effect size for the percentage deviation from expected coronary artery calcification with the genetic risk score for CAD and CAC.

	Percent deviation from the expected (CAC_5y_+1), (95% CI), p-value	Explained variance (%)
	**Model 1 log(obs)–log(exp)~CAD GRS+age+sex+log(CAC_b_+1)**	
Intercept	-63.3 (-74.2; -47.8), <0.0001	
**CAD GRS**	**9.7 (5.2; 14.5), 1.6x10**^**-5**^	**0.6**
Age (years)	2.7 (2.1; 3.3), <0.0001	
Sex	-16.9 (-24.1; -8.9), <0.0001	
log(CAC_b_+1)	-8.9 (-10.7; -7.0), <0.0001	
	**Model 2 log(obs)–log(exp)~CAD GRS+age+sex+log(CAC**_**b**_**+1)+ diabetes+BMI+systolic blood pressure+smoking+use of antihypertensive+lipid lowering medication+social economic status+LDL-cholesterol+HDL-cholesterol**	
Intercept	-61.4 (-78.6; -30.2), 0.002	
**CAD GRS**	**9.0 (4.4; 13.8), 1.6x10**^**-5**^	
Age (years)	2.5 (1.9; 3.2), <0.0001	
Sex	-13.8 (-22.8; -3.7), 0.009	
log(CAC_b_+1)	-10.6 (-12.5; -8.6), <0.0001	
Diabetes	42.0 (23.4; 63.4), <0.0001	
BMI (per kg/m^2^)	-3.2 (-4.2;-2.1), <0.0001	
Systolic blood pressure (per mmHg)	0.5 (0.3; 0.7), <0.0001	
LDL-cholesterol (per mg/dL)	0.2 (0.04; 0.3), 0.009	
HDL-cholesterol (per mg/dL)	-0.1 (-0.4; 0.2), 0.53	
Social economic status	-7.6 (-16.1; 1.9), 0.11	
Antihypertensive medication	13.6 (2.8; 25.4), 0.01	
Lipid-lowering medication	16.8 (0.6; 35.6), 0.04	
Current smoker	34.9 (20.0; 51.7), <0.0001	
Past smoker	10.7 (0; 22.4), 0.05	
	**Model 3 log(obs)–log(exp)~CAC GRS+age+sex+log(CAC**_**b**_**+1)**	
Intercept	-63.1 (-74.1; -47.4), <0.0001	
**CAC GRS**	**6.2 (1.9; 10.8), 0.005**	**0.2**
Age (years)	2.7 (2.1; 3.3), <0.0001	
Sex	-16.5 (-23.8; -8.5), 0.0001	
log(CAC_b_+1)	-8.6 (-10.5; -6.7), <0.0001	
	**Model 4 log(obs)–log(exp)~CAC GRS+age+sex+log(CAC**_**b**_**+1)+diabetes+BMI+systolic blood pressure+smoking+use of antihypertensive+lipid lowering medication+social economic status+LDL-cholesterol+HDL-cholesterol**	
Intercept	-63.8 (-80.1; -34.2), 0.009	
**CAC GRS**	6.8 (2.3; 11.5), 0.003	
Age (years)	2.5 (1.8; 3.2), <0.0001	
Sex	-13.2 (-22.3; -3.0), 0.01	
log(CAC_b_+1)	-10.4 (-12.3; -8.4), <0.0001	
Diabetes	42.7 (23.9; 64.2), <0.0001	
BMI (per kg/m^2^)	-3.3 (-4.4;-2.3), <0.0001	
Systolic blood pressure (per mmHg)	0.5 (0.3; 0.8), <0.0001	
LDL-cholesterol (per mg/dL)	0.2 (0.1; 0.3), 0.003	
HDL-cholesterol (per mg/dL)	-0.1 (-0.4; 0.2), 0.43	
Social economic status	-7.2 (-15.8; 2.3), 0.13	
Antihypertensive medication	13.5 (2.7; 25.4), 0.01	
Lipid-lowering medication	17.8 (1.4; 36.8), 0.03	
Current smoker	33.7 (18.9; 50.3), <0.0001	
Past smoker	10.1 (-0.5; 21.8), 0.06	

GRS: genetic risk score, CAD: coronary artery disease, CAC: coronary artery calcification, LDL: low-density lipoprotein, HDL: high-density lipoprotein and EV: explained variance in percent compared to the model without GRS adjusted for age, sex and log(CAC_b_+1). The association between the genetic risk scores and outcome was carried out using linear regression in SAS. Model 1 and model 3 are adjusted for age, sex and log(CAC_b_+1) and model 2 and model 4 are adjusted for age, sex, log(CAC_b_+1), diabetes, BMI, systolic blood pressure, smoking, use of antihypertensive and lipid lowering medication, social economic status, LDL-cholesterol and HDL-cholesterol. We subtracted the explained variance of the baseline model i.e. adjusted for age, sex and log(CAC_b_+1) to estimate the explained variance because of the genetic risk score.

**Table 3:** Estimated effect size for the 5-year progression of coronary artery calcification with the genetic risk score for CAD and CAC.

	Percent change in (CAC+1), (95% CI), p-value	Explained variance (%)
	**Model 1 Progression of CAC~CAD GRS+age+sex+log(CAC**_**b**_**+1)**	
Intercept	-42.1 (-58.2; -19.8), 0.001	
**CAD GRS**	**7.1 (3.0; 11.4), 0.0005**	**0.4**
Age (years)	2.6 (2.1; 3.2), <0.0001	
Sex	-18.3 (-24.9; -11.1), <0.0001	
log(CAC_b_+1)	-5.3 (-7.0; -3.4), <0.0001	
	**Model 2 Progression of CAC~CAD GRS+age+sex+log(CAC**_**b**_**+1) +diabetes+BMI+systolic blood pressure+smoking+use of antihypertensive+lipid lowering medication+social economic status+LDL-cholesterol+HDL-cholesterol**	
Intercept	-55.4 (-74.4; -22.5), 0.004	
**CAD GRS**	**6.4 (2.3; 10.8), 0.002**	
Age (years)	2.4 (1.8; 3.0), <0.0001	
Sex	-14.9 (-23.2; -5.7), 0.002	
log(CAC_b_+1)	-7.0 (-8.9; -5.1), <0.0001	
Diabetes	34.6 (18.1; 53.3), <0.0001	
BMI (per kg/m^2^)	-2.4 (-3.4; -1.4), <0.0001	
Systolic blood pressure (per mmHg)	0.5 (0.3; 0.7), <0.0001	
LDL-cholesterol (per mg/dL)	0.2 (0.1; 0.3), 0.001	
HDL-cholesterol (per mg/dL)	-0.1 (-0.4; 0.2), 0.51	
Social economic status	-7.6 (-15.5; 1.1), 0.09	
Antihypertensive medication	11.9 (2.1; 22.8), 0.02	
Lipid-lowering medication	15.2 (0.3; 32.3), 0.05	
Current smoker	35.1 (21.2; 50.6), <0.0001	
Past smoker	9.0 (-0.8; 19.6), 0.07	
	**Model 3 Progression of CAC~CAC GRS+age+sex+log(CAC**_**b**_**+1)**	
Intercept	-41.8 (-58.0; -19.4), 0.001	
**CAC GRS**	**4.4 (0.4; 8.5), 0.03**	**0.1**
Age (years)	2.6 (2.0; 3.2), <0.0001	
Sex	-18.0 (-24.6; -10.8), <0.0001	
log(CAC_b_+1)	-5.1 (-6.9; -3.2), <0.0001	

GRS: genetic risk score, CAD: coronary artery disease, CAC: coronary artery calcification, LDL: low-density lipoprotein, HDL: high-density lipoprotein and EV: explained variance in percent compared to the model without GRS adjusted for age, sex and log(CAC_b_+1). The association between the genetic risk scores and outcome was carried out using linear regression in SAS. Model 1 and model 3 are adjusted for age, sex and log(CAC_b_+1) and model 2 is adjusted for age, sex, log(CAC_b_+1), diabetes, BMI, systolic blood pressure, smoking, use of antihypertensive and lipid lowering medication, social economic status, LDL-cholesterol and HDL-cholesterol. We subtracted the explained variance of the baseline model i.e. adjusted for age, sex and log(CAC_b_+1) to estimate the explained variance because of the genetic risk score.

The associations of the genetic risk score related to the CAD risk factors with “log(obs)–log(exp)” and the progression of CAC are shown in Tables 2 and 3 and S1(A) Table and S1(B) Table. The CAC genetic risk score and the TC genetic risk score were associated with the deviation from the expected (CAC_5y_+1). A per-SD increase in the CAC genetic risk score and the TC genetic risk score increased the “log(obs)–log(exp)” by 6.2% ((1.9%; 10.8%), p = 0.005) and 5.3% ((1.0%; 9.8%), p = 0.02) respectively. Similarly, a per-SD increase in the CAC genetic risk score and the TC genetic risk score increased the progression of CAC by 4.4% ((0.4%; 8.5%), p = 0.03) and 4.5% ((0.6%; 8.6%), p = 0.02), respectively. However, after controlling for multiple testing, only the association between the CAC genetic risk score and the deviation from the expected (CAC_5y_+1) remained significant. Tables 2 and 3 further shows that age, sex and log(CAC_b_+1) contributed significantly to the “log(obs)-log(exp)” and the progression of CAC outcomes (Model 3). The CAC genetic risk score explained 0.2% of the observed variance with “log(obs)-log(exp)” and 0.1% with the progression of CAC compared to the base model (Tables 2 and 3). None of the other genetic risk scores showed any association with “log(obs)–log(exp)” or with the progression of CAC and the variance explained by their respective models ranged between 0–0.2% (S1(A) Table and S1(B) Table). After adjusting for risk factors, the CAD and CAC genetic risk scores were significantly associated with “log(obs)–log(exp)” and the CAD genetic risk score was significantly associated with the progression of CAC (Tables 2 and 3). Summary statistics for the association between the individual SNPs with “log(obs)–log(exp)” and the progression of CAC are shown in (S2A Table–S2K Table). We then determined which of the CAD and CAC SNPs showed an association at the nominal significance level with the progression of CAC in our study. The CAD- (10% SNPs) and CAC- (66.7% SNPs) associated risk alleles were associated with an increased level of CAC progression in our study (S3 Table), indicating that the alleles that increase the risk for CAD or increase the level of CAC collectively tend to increase the CAC progression.

We additionally combined the nonoverlapping CAD and CAC associated SNPs (72 SNPs) into a single genetic risk score and tested its association with the deviation from expected (CAC_5y_+1) and the progression of CAC. The effect size and the explained phenotypic variation hardly changed for the deviation from expected (CAC_5y_+1) [10.0% (5.4%; 14.7%), p = 1.1x10^-5^ (explained phenotypic variance = 0.6%)] as well as for the progression of CAC [7.2% (3.1%; 11.4%), p = 0.0005 (explained phenotypic variance = 0.4%), data not shown]. To determine whether the prediction of CAC progression could be improved further, we included CAD and CAC genetic risk scores as separate predictors in a linear regression model. The explained phenotypic variation hardly changed (explained phenotypic variance = 0.6%) for the deviation from expected (CAC_5y_+1) phenotype or the progression of CAC (explained phenotypic variance = 0.4%) (S4 Table). Furthermore, we investigated the joint role of the three CAC SNPs by including them as separate predictors in a single linear model. The explained phenotypic variation improved minimally (deviation from expected (CAC_5y_+1) = 0.3% and progression of CAC = 0.2%); however, the effect of the association of individual SNP with the deviation from expected (CAC_5y_+1) (rs9349379: 4.0% (-2.1%; 10.6%), p = 0.21; rs10965219: 4.9% (-2.3%; 12.5%), p = 0.19 and rs1333049: 3.7% (-3.4%; 11.4%), p = 0.31) and the progression of CAC (rs9349379: 2.5% (-3.1%; 8.4%), p = 0.39; rs10965219: 3.4% (-3.1%; 10.3%), p = 0.31 and rs1333049: 2.8% (-3.8%; 9.8%), p = 0.42) were not significant (data not shown).

Since CAD and CAC genetic risk scores showed an association with “log(obs)–log(exp)” and the CAD genetic risk score showed an association with the progression of CAC, we further performed four sensitivity analyses: i) we carried out 75^th^ percentile quantile regression as the phenotypes were heavily tailed; ii) we looked at the model with covariates possibly showing heteroscedasticity; iii) we looked at whether the genetic risk score provided additional information relative to the family history of CHD (S5 Table); and iv) we divided the CAD and CAC genetic risk scores into quartiles, and the risk for “log(obs)–log(exp)” and the progression of CAC was tested in each group, using Q1 as a reference (S6(A) Table and S6(B) Table).

The per-SD increase in the CAD genetic risk score increased the deviation from the expected (CAC_5y_+1) by 12.9% ((7.0%; 19.1%), p = 9.08x10^-6^) and the progression of CAC by 5.9% ((1.5%; 10.4%), p = 0.008) in the 75^th^ percentile in the quantile regression. However, the per-SD increase in the CAC genetic risk score showed a nonsignificant association with the deviation from the expected (CAC_5y_+1) (5.5% ((-0.8%; 12.1%), p = 0.09) in the 75^th^ percentile in the quantile regression (data not shown).

Calculating the models for the CAD genetic risk score and the CAC genetic risk score consistent with possible heteroscedasticity in covariates only marginally altered the results for both outcomes.

S5 Table shows the association between the genetic risk scores with “log(obs)–log(exp)” and the progression of CAC after adjusting for family history of CHD. The CAD genetic risk score explains an additional 0.5% (log(obs)–log(exp)) and 0.3% (progression of CAC) of the observed variance compared to the model adjusted for age, sex, log(CAC_b_+1) and family history (R^2^ = 3.6% for log(obs)–log(exp) and 3.1% for the progression of CAC; data not shown). The effect size and explained variance remained similar to the analyses without information on family history of CHD (Tables 2 and 3). Similar results were obtained for the CAC genetic risk score.

For “log(obs)–log(exp)” analyses, those with a higher CAD genetic risk score and CAC genetic risk score (Q4) had a 26.3% ((12.2%; 42.3%), p = 0.0001) and 17.5% ((3.9%; 33.0%), p = 0.01) higher percent deviation from the expected (CAC_5y_+1) compared to those with lower CAD and CAC genetic risk scores respectively (S6(A) Table). Similarly, those with a higher CAD genetic risk score had a 21.2% ((8.6%; 35.3%), p = 0.0006) increased progression of CAC compared with those with a lower CAD genetic risk score in the model adjusted for age, sex and log(CAC_b_+1) (S6(B) Table). After adjustment for the risk factors, the higher quartile of CAD genetic risk score remained significantly associated with “log(obs)–log(exp)” and the progression of CAC. The higher quartile for CAC genetic risk score also remained associated with “log(obs)–log(exp)” (S6(A) Table and S6(B) Table).

## Discussion & conclusion

Our study shows that the progression of CAC was associated with the genetic risk score for coronary artery disease. We also found a positive relationship between the genetic risk score for coronary artery calcification and the progression of CAC.

The result for the CAC genetic risk score constructed using three CAC SNPs is consistent with the findings from an observational study showing that the baseline CAC is one of the predictors for the progression of CAC [46]. Two of the three CAC SNPs also showed a positive association with the progression of CAC in our study. In one of the studies, investigating the role of the CAC genetic risk score for the association with the burden of calcification in different vessels showed a positive association of the CAC genetic risk score with calcification in the aortic arch and the extracranial and intracranial carotid arteries [47]. However, in our analysis investigating the joint role of three CAC SNPs as multiple predictors did not show any significant association of the three SNPs with the percent deviation from expected (CAC_5y_+1) or the progression of CAC. The percent of the phenotypic variance explained by the CAC genetic risk score as well as the joint role of three CAC SNPs in our study was low (0.1–0.3%), indicating that the CAC genetic risk score and the three known CAC SNPs predict the traits poorly. However, we expect that there are several other CAC-associated common genetic variants that have yet to be discovered and might play an important role in the progression of CAC. All the GWAS analyses conducted on CAC consisted of smaller sample sizes (largest sample size = 9961) compared to the GWAS meta-analyses for other phenotypes (CAD, BMI, T2D, lipids and blood pressure traits); hence, the CAC genetic risk score is much less comprehensive than those for the other phenotypes.

The association of the CAD genetic risk score constructed using 70 known CAD SNPs explained an additional 0.4–0.6% of the phenotypic variation of the progression of CAC. Out of the 70 known CAD SNPs, only rs1333049 (*CDKN2A/B* at 9p21) is present in the CAC genetic risk score. The two *PHACTR1* loci (rs9349379 (CAC SNP) and rs12526453 (CAD SNP)) are 23587 base pairs apart and are not in LD (r^2^ = 0.32). Since there is an overlap of 1 (rs1333049) out of 3 CAC SNPs with the CAD SNPs, we performed an analysis by constructing a new genetic risk score consisting of nonoverlapping CAC and CAD SNPs. However, the effect size and the explained phenotypic variance hardly changed. In the analysis using the CAD and CAC genetic risk scores as separate factors in a linear regression model, the explained phenotypic variation hardly changed for the progression of CAC and improved minimally for the deviation from the expected (CAC_5y_+1) phenotype, demonstrating that the prediction of CAC progression could not be further improved by using the information from both the CAD and CAC genetic risk scores in a single model. In a study investigating the association of the CAD genetic risk score with the cross-sectional value of CAC, a positive association between the genetic risk score and CAC was observed [48]. In a recent study, using different algorithms for the progression of CAC, CAC progression was shown to be associated with coronary and cardiovascular events [1]. The association of the CAD genetic risk score with the progression of CAC strengthens our understanding that the progression of CAC and cross-sectional CAC are risk factors for CHD.

The CAD (10%) and CAC (66.7%) SNPs in our study showed a suggestive association with the CAC progression. These associations are also not surprising, as the progression of CAC is known to be correlated with CAD. In our study, the contribution of the CAC and CAD genetic risk score models to the variance explained for progression of CAC was small, but it is consistent with the variance explained in several GWAS analyses where these common variants explain only a fraction of the phenotypic variance Klicken Sie hier, um Text einzugeben. Additionally, considering the phenotypic heterogeneity among several CAD GWAS analyses, it is not direct to comprehend the size of the effect of CAD SNPs on the progression of CAC phenotype. Constructing the unweighted CAD and CAC genetic risk scores i.e., ignoring the effect of the SNPs, produced similar results for the percent deviation from the expected (CAC_5y_+1) (CAC_GeneticRiskScore_: 6.2% (1.8%; 10.8%), p = 0.005 and CAD_GeneticRiskScore_: 7.8% (3.3%; 12.4%), p = 0.0005) and the progression of CAC (CAC_GeneticRiskScore_: 4.4% (0.4%; 8.5%), p = 0.03 and CAD_GeneticRiskScore_: 5.7% (1.6%; 9.8%), p = 0.006) in our study (data not shown).

Observational studies have shown a positive association between total cholesterol and LDL-cholesterol levels and the progression of coronary artery calcification [13–15]. Studies have shown that several of the TC-associated SNPs as well as the TC genetic risk score are also associated with CAD [20,24,34]. However, our finding regarding the association of the TC genetic risk score with the progression of CAC did not remain significant after controlling for multiple testing. In our study, we also did not observe a significant association of the LDL-cholesterol genetic risk score with the progression of CAC, which is surprising as the LDL-cholesterol genetic risk score has been shown to be associated with CAD [20,24,34]. Although few of the observational studies have shown the association of HDL-cholesterol level with the progression of CAC [13,14,19], we did not observe any significant associations for the HDL-cholesterol genetic risk score in our study. The result of our study is consistent with studies showing that genes influencing HDL-cholesterol may not have a significant impact on CAD/MI risk; hence, we can speculate that the HDL-cholesterol genetic risk score also does not influence the progression of CAC [49,50]. Additionally, we did not observe any association of the triglyceride genetic risk score with the progression of CAC. In studies assessing the association between the triglyceride genetic risk score and CHD, the triglyceride genetic risk score was associated with CHD only in one of the study subgroups [50,51].

In several observational studies, diabetes mellitus has been shown to be one of the risk factors for the progression of CAC [13–15,19,52]. However, the T2D genetic risk score showed no association with the progression of CAC in our study. One possible explanation could be that there are several diabetes-associated common variants that are yet to be discovered, or it could be that the pathway/s through which diabetes influences the progression of CAC is modulated by the use of medication; one such example is statin use. In a prospective study carried out in type 2 diabetic subjects, statin use was shown to be a risk factor for the progression of CAC [52]. BMI has been shown to be associated with the progression of CAC in observational studies [8,14,19]. However, the BMI genetic risk score in our study was not associated with the progression of CAC.

However, despite the association between high blood pressure and the risk of CHD in observational as well as in genetic studies, we did not observe any association of the genetic risk scores for systolic blood pressure, diastolic blood pressure and pulse pressure with the progression of CAC in our study [14,15,19,27,53].

The strengths of the present study are the longer follow-up scan time of 5 years and the use of two definitions for the progression of CAC (the deviation between the log-transformed observed CAC_5y_ and expected CAC_5y_ and the difference between log-transformed CAC_5y_ and CAC_b_). It would be interesting to see if the results of our study could be replicated in other studies. Furthermore, in our study we excluded those individuals with stent implementation, bypass, balloon dilatation or myocardial infarction during the 5-year follow-up because different revascularization procedures would have disturbed the CAC score measurement [17]. It is known that statins influences the degree of CAC progression [54] and hence we have adjusted our analyses for the use of lipid medications. However, we could not rule out the influence of statins use between the baseline and first follow-up on the progression of CAC in our study. From our results the baseline log(CAC_b_+1) showed a negative impact on the progression of CAC which suggests a higher baseline CAC was associated with a lower proportional increase, however absolute Agatston score values increases more in those with higher than in those with lower baseline CAC. Regarding negative results, the lack of an association for the TC and LDL-cholesterol genetic risk scores with the progression of CAC could be because of the modest sample size of our study relative to the smaller effect size observed for any of the individual SNPs. Hence, the precision available for such interpretations is extremely limited. Additionally, it is possible that the environmental drivers of CAD risk factors, such as diet and exercise, overwhelm the effects of genetics on CAC progression; therefore, even though there might be some small effects, they are unimportant and would require an enormous sample to detect the associations. Therefore, the apparent low precision in our study prevents us from drawing any strong or meaningful conclusions from our results regarding the associations of the genetic risk scores related to the CAD risk factors with the progression of CAC. Also, the effect size of the association of the CAD genetic risk score with CAC progression as well as the phenotypic variance explained in our study was small, further studies in large cohorts are needed to first confirm the findings of our study. Once the results are confirmed in larger studies, additional studies will then be necessary to test for the clinical utility of including CAD GRS into current risk models for progression of CAC. This may eventually help to better identify those individuals at highest risk and can contribute to reducing the number of coronary events in the general population.

In conclusion, in the present study, we investigated the effect of the genetic risk scores associated with CAD and traditional CAD risk factors with two different algorithms for the progression of CAC. The genetic risk scores associated with CAD and CAC are involved with continuous measures for the progression of CAC, suggesting that the risk associated with CAD is facilitated through the calcification of arteries. Collaborative work with larger studies or consortium will be useful to identify novel loci leading to the progression of CAC.

## Supporting information

S1 Table a) Estimated effect size for the percentage deviation from the expected coronary artery calcification with the genetic risk scores for coronary artery disease and coronary artery calcification. CAD: coronary artery disease, CAC: coronary artery disease, LDL: low-density lipoprotein, HDL: high-density lipoprotein. The association between the genetic risk scores and the outcome was carried out using linear regression in SAS. The models are adjusted for age, sex, log(CAC_b_+1), diabetes, BMI, systolic blood pressure, smoking, use of antihypertensive and lipid lowering medication, social economic status, LDL and HDL. b) Estimated effect size for the 5-year progression of coronary artery calcification with the coronary artery disease genetic risk score. CAD: coronary artery disease, CAC: coronary artery disease, LDL: low density lipoprotein, HDL: high density lipoprotein. The association between the genetic risk score and the outcome was carried out using linear regression in SAS. The model is adjusted for age, sex, log(CAC_b_+1), diabetes, BMI, systolic blood pressure, smoking, use of antihypertensive and lipid lowering medication, social economic status, LDL and HDL.
(DOCX)

S2 Table a) Association of coronary artery diseases-associated SNPs with log(obs)–log(exp) and the 5-year progression of CAC in the Heinz Nixdorf Recall study. b) Association of coronary artery calcification-associated SNPs with log(obs)–log(exp) and the 5-year progression of CAC in the Heinz Nixdorf Recall study. c) Association of diabetes-associated SNPs with log(obs)–log(exp) and the 5-year progression of CAC in the Heinz Nixdorf Recall study. d) Association of body mass index-associated SNPs with log(obs)–log(exp) and the 5-year progression of CAC in the Heinz Nixdorf Recall study. e) Association of systolic blood pressure-associated SNPs with log(obs)–log(exp) and the 5-year progression of CAC in the Heinz Nixdorf Recall study. f) Association of diastolic blood pressure-associated SNPs with log(obs)–log(exp) and the 5-year progression of CAC in the Heinz Nixdorf Recall study. g) Association of pulse pressure-associated SNPs with log(obs)–log(exp) and the 5-year progression of CAC in the Heinz Nixdorf Recall study. h) Association of low-density lipoprotein-cholesterol-associated SNPs with log(obs)–log(exp) and the 5-year progression of CAC in the Heinz Nixdorf Recall study. i) Association of high-density lipoprotein-cholesterol-associated SNPs with log(obs)–log(exp) and the 5-year progression of CAC in the Heinz Nixdorf Recall study. j) Association of triglyceride-associated SNPs with log(obs)–log(exp) and the 5-year progression of CAC in the Heinz Nixdorf Recall study. k) Association of total cholesterol-associated SNPs with log(obs)–log(exp) and the 5-year progression of CAC in the Heinz Nixdorf Recall study. CHR: chromosome, BP: base position (hgBuild37), CA: coded allele, NCA: non coded allele, CAF: coded allele frequency, 95%CI: 95% confidence interval, CAC: coronary artery calcification, “log(obs)–log(exp)”: percent deviation from the expected (CAC_5y_+1). The association between each SNP and outcomes was carried out using linear regression in PLINK. The models are adjusted for age, sex and log(CAC_b_ + 1).
(DOCX)

S3 Table Association of the coronary artery disease and coronary artery calcification SNPs that showed an association with log(obs)–log(exp) or the 5-year progression in CAC or both at the nominal significance level. CAD: coronary artery disease, CAC: coronary artery calcification. CHR: chromosome, BP: base position (hgBuild37), CA: coded allele, NCA: non coded allele, CAF: coded allele frequency, 95%CI: 95% confidence interval, CAC: coronary artery calcification, “log(obs)–log(exp)”: percent deviation from the expected (CAC_5y_+1). The association between each SNP and outcomes was carried out using linear regression in PLINK. The models are adjusted for age, sex and log(CAC_b_+1).
(DOCX)

S4 Table Association between the CAD and CAC genetic risk scores as separate predictors in a linear regression model with log(obs)–log(exp) and the 5-year progression in CAC. CAD: coronary artery disease, CAC: coronary artery calcification, CADPlusCAC: CAD and CAC genetic risk scores are included as separate predictors in a linear regression model, EV: explained variance. The association between the genetic risk scores and outcomes was carried out using linear regression in SAS. The models are adjusted for age, sex and log(CAC_b_+1).
(DOCX)

S5 Table Association between the genetic risk scores with log(obs)–log(exp) and the 5-year progression in CAC adjusted for a family history of CHD. CAD: coronary artery disease, CAC: coronary artery calcification, CAD_CAC: combined CAD and CAC genetic risk score, BMI: body-mass index, TC: total cholesterol, EV: explained variance. The association between the genetic risk scores and outcomes was carried out using linear regression in SAS. The models are adjusted for age, sex, log(CAC_b_+1) and family history of CHD.
(DOCX)

S6 Table a) Association of the genetic risk score quartiles with the percentage deviation from the expected coronary artery calcification. GRS: genetic risk score, CAC: coronary artery calcification, CI: confidence interval, medium GRS quartile consists of Q2 and Q3 and high GRS quartile consist of Q4. The association between the genetic risk scores and outcome was carried out using linear regression in SAS. Model 1: adjusted for age, sex and log(CAC_b_+1). Model 2: adjusted for age, sex, log(CAC_b_+1) and coronary artery disease risk factors (type 2 diabetes, body mass index, socio economic status, systolic blood pressure, smoking, antihypertensive medication, lipid lowering medication, LDL, HDL). b) Association of the genetic risk score quartiles with the 5-year progression of coronary artery calcification. GRS: genetic risk score, CAD: coronary artery disease, CI: confidence interval, medium GRS quartile consists of Q2 and Q3 and high GRS quartile consist of Q4. The association between the genetic risk score and outcome was carried out using linear regression in SAS. Model 1: adjusted for age, sex and log(CAC_b_+1). Model 2: adjusted for age, sex, log(CAC_b_+1) and CAD risk factors (type 2 diabetes, body mass index, socio economic status, systolic blood pressure, smoking, antihypertensive medication, lipid lowering medication, LDL, HDL).
(DOCX)

S7 Table a): R2 between the coronary artery diseases-associated SNPs. b): R2 between the coronary artery calcification-associated SNPs. c): R2 between the type 2 diabetes-associated SNPs. d): R2 between the body mass index-associated SNPs. e): R2 between the systolic blood pressure-associated SNPs. f): R2 between the diastolic blood pressure-associated SNPs. g): R2 between the pulse pressure-associated SNPs. h): R2 between the low-density lipoprotein-cholesterol-associated SNPs. i): R2 between the high-density lipoprotein-cholesterol-associated SNPs. j): R2 between the triglyceride-associated SNPs. k): R2 between the total cholesterol-associated SNPs.
(XLSX)
