# Supplementary material for: Genetic risk scores for coronary artery disease and its traditional risk factors: Their role in the progression of coronary artery calcification—Results of the Heinz Nixdorf Recall study
Source: PLoS One. 2020 May 7;15(5):e0232735. doi: 10.1371/journal.pone.0232735 (PMC7205301; doi:10.1371/journal.pone.0232735)
Supplement: S1 Table — a) Estimated effect size for the percentage deviation from the expected coronary artery calcification with the genetic risk scores for coronary artery disease and coronary artery calcification. CAD: coronary artery disease, CAC: coronary artery disease, LDL: low-density lipoprotein, HDL: high-density lipoprotein. The association between the genetic risk scores and the outcome was carried out using linear regression in SAS. The models are adjusted for age, sex, log(CACb+1), diabetes, BMI, systolic blood pressure, smoking, use of antihypertensive and lipid lowering medication, social economic status, LDL and HDL. b) Estimated effect size for the 5-year progression of coronary artery calcification with the coronary artery disease genetic risk score. CAD: coronary artery disease, CAC: coronary artery disease, LDL: low density lipoprotein, HDL: high density lipoprotein. The association between the genetic risk score and the outcome was carried out using linear regression in SAS. The model is adjusted for age, sex, log(CACb+1), diabetes, BMI, systolic blood pressure, smoking, use of antihypertensive and lipid lowering medication, social economic status, LDL and HDL. (DOCX) [file pone.0232735.s001.docx]

Table S1 (a): Estimated effect size for the percentage deviation from the expected coronary artery calcification with the genetic risk scores for CAD-associated risk factors.

|  | **Percent deviation from the expected (CAC_5y_+1),**  **(95% CI), p-value** | **Explained variance**  **(%)** |
| --- | --- | --- |
| Intercept | -62.9 (-74.0; -47.1), <0.0001 |  |
| **Type 2 diabetes GRS** | **1.0 (-3.1; 5.3), 0.63** | **0.01** |
| Age (years) | 2.6 (2.0; 3.3), <0.0001 |  |
| Sex | -15.6 (-23.0; -7.6), 0.0003 |  |
| log(CAC_b_+1) | -8.3 (-10.2; -6.4), <0.0001 |  |
| Intercept  **Body mass index GRS** | -62.7 (-73.8; -46.8), <0.0001  **-2.8 (-6.8; 1.3), 0.18** | **0.06** |
| Age (years) | 2.6 (2.0; 3.2), <0.0001 |  |
| Sex | -15.5 (-22.9; -7.5), 0.0003 |  |
| log(CAC_b_+1) | -8.3 (-10.2; -6.4), <0.0001 |  |
| Intercept | -63.1 (-74.1; 47.4), <0.0001 |  |
| **Systolic blood pressure GRS** | **2.3 (-1.9; 6.7), 0.29** | **0.04** |
| Age (years) | 2.6 (2.0; 3.3), <0.0001 |  |
| Sex | -15.5 (-22.8; -7.4), 0.0003 |  |
| log(CAC_b_+1) | -8.3 (-10.2; -6.4), <0.0001 |  |
| Intercept | -62.9 (-74.0; -47.2), <0.0001 |  |
| **Diastolic blood pressure GRS** | **1.5 (-2.7; 5.8), 0.50** | **0.01** |
| Age (years) | 2.6 (2.0; 3.3), <0.0001 |  |
| Sex | -15.6 (-23.0; -7.6), 0.0003 |  |
| log(CAC_b_+1) | -8.3 (-10.2; -6.4), <0.0001 |  |
| Intercept | -62.9 (-74.0; -47.2), <0.0001 |  |
| **Pulse pressure GRS** | **-0.7 (-4.8; 3.5), 0.74** | **0.004** |
| Age (years) | 2.6 (2.0; 3.3), <0.0001 |  |
| Sex | -15.6 (-23.0; -7.6), 0.0003 |  |
| log(CAC_b_+1) | -8.3 (-10.2; -6.4), <0.0001 |  |
| Intercept | -63.1 (-74.1; -47.4), <0.0001 |  |
| **LDL-cholesterol GRS** | **3.8 (-0.5; 8.2), 0.08** | **0.1** |
| Age (years) | 2.7 (2.1; 3.3), <0.0001 |  |
| Sex | -15.8 (-23.2; -7.8), 0.0002 |  |
| log(CAC_b_+1) | -8.4 (-10.3; -6.5), <0.0001 |  |
| Intercept | -62.8 (-73.9; -47.1), <0.0001 |  |
| **HDL-cholesterol GRS** | **0.6 (-3.6; 4.8), 0.79** | **0.002** |
| Age (years) | 2.6 (2.0; 3.3), <0.0001 |  |
| Sex | -15.6 (-23.0; -7.6), 0.0003 |  |
| log(CAC_b_+1) | -8.3 (-10.2; -6.4), <0.0001 |  |
| Intercept | -62.9 (-73.9; -47.1), <0.0001 |  |
| **Triglycerides GRS** | **0.1 (-4.0; 4.4), 0.96** | **0** |
| Age | 2.6 (2.0; 3.3), <0.0001 |  |
| Sex | -15.6 (-23.0; -7.6), 0.0003 |  |
| log(CAC_b_+1) | -8.3 (-10.2; -6.4), <0.0001 |  |
| Intercept | -62.8 (-73.9; -47.0), <0.0001 |  |
| **Total cholesterol GRS** | **5.3 (1.0; 9.8), 0.02** | **0.2** |
| Age (years) | 2.7 (2.0; 3.3), <0.0001 |  |
| Sex | -15.9 (-23.3; -7.9), 0.0002 |  |
| log(CAC_b_+1) | -8.4 (-10.3; -6.5), <0.0001 |  |

GRS: genetic risk score, CAC: coronary artery calcification, LDL: low-density lipoprotein, HDL: high-density lipoprotein and EV: explained variance in percent compared to the model without GRS adjusted for age, sex and log(CAC_b_+1). The association between the genetic risk scores and outcome was carried out using linear regression in SAS. The model is adjusted for age, sex and log(CAC_b_+1). We subtracted the explained variance of the baseline model i.e. adjusted for age, sex and log(CAC_b_+1) to estimate the explained variance because of the genetic risk score.

Table S1 (b): Estimated effect size for the 5-year progression of coronary artery calcification with the genetic risk score for CAD-associated risk factors.

|  | **Percent change in (CAC+1),**  **(95% CI), p-value** | **Explained variance**  **(%)** |
| --- | --- | --- |
| Intercept | -41.6 (-57.8; -19.1), 0.001 |  |
| **Type 2 diabetes GRS** | **0.4 (-3.4; 4.3), 0.84** | **0.001** |
| Age (years) | 2.6 (2.0; 3.1), <0.0001 |  |
| Sex | -17.4 (-24.0; -10.1), <0.0001 |  |
| log(CAC_b_+1) | -4.8 (-6.6; -3.0), <0.0001 |  |
| Intercept | -41.3 (-57.7; -18.7), 0.001 |  |
| **Body mass index GRS** | **-2.0 (-5.7; 1.9), 0.32** | **0.03** |
| Age (years) | 2.5 (2.0; 3.1), <0.0001 |  |
| Sex | -17.3 (-24.0; -10.1), <0.0001 |  |
| log(CAC_b_+1) | -4.8 (-6.6; -3.0), <0.0001 |  |
| Intercept | -41.8 (-58.0; -19.4), 0.001 |  |
| **Systolic blood pressure GRS** | **2.3 (-1.5; 6.4), 0.24** | **0.04** |
| Age (years) | 2.6 (2.0; 3.1), <0.0001 |  |
| Sex | -17.2 (-23.9; -10.0), <0.0001 |  |
| log(CAC_b_+1) | -4.8 (-6.6; -3.0), <0.0001 |  |
| Intercept | -41.6 (-57.8; -19.1), 0.001 |  |
| **Diastolic blood pressure GRS** | **1.4 (-2.4; 5.4), 0.47** | **0.02** |
| Age (years) | 2.6 (2.0; 3.1), <0.0001 |  |
| Sex | -17.4 (-24.0; -10.1), <0.0001 |  |
| log(CAC_b_+1) | -4.8 (-6.6; -3.0), <0.0001 |  |
| Intercept | -41.6 (-57.9; -19.1),0.001 |  |
| **Pulse pressure GRS** | **-0.4 (-4.2; 3.5), 0.82** | **0.002** |
| Age (years) | 2.6 (2.0; 3.1), <0.0001 |  |
| Sex | -17.4 (-24.0; -10.1), <0.0001 |  |
| log(CAC_b_+1) | -4.8 (-6.6; -3.0), <0.0001 |  |
| Intercept | -41.8 (-58.0; -19.4), 0.001 |  |
| **LDL-cholesterol GRS** | **3.2 (-0.7; 7.3), 0.11** | **0.1** |
| Age (years) | 2.6 (2.0; 3.1), <0.0001 |  |
| Sex | -17.6 (-24.2; -10.3), <0.0001 |  |
| log(CAC_b_+1) | -4.9 (-6.7; -3.1), <0.0001 |  |
| Intercept | -41.5 (-57.8; -19.0), 0.001 |  |
| **HDL-cholesterol GRS** | **0.4 (-3.4; 4.4), 0.83** | **0.001** |
| Age (years) | 2.6 (2.0; 3.1), <0.0001 |  |
| Sex | -17.4 (-24.0; -10.1), <0.0001 |  |
| log(CAC_b_+1) | -4.8 (-6.6; -3.0), <0.0001 |  |
| Intercept | -41.6 (-57.9; -19.1), 0.001 |  |
| **Triglycerides GRS** | **-0.6 (-4.4; 3.3), 0.75** | **0.003** |
| Age | 2.6 (2.0; 3.1), <0.0001 |  |
| Sex | -17.3 (-24.0; -10.1), <0.0001 |  |
| log(CAC_b_+1) | -4.8 (-6.6; -3.0), <0.0001 |  |
| Intercept | -41.4 (-57.7; -18.8), 0.001 |  |
| **Total cholesterol GRS** | **4.5 (0.6; 8.6), 0.02** | **0.2** |
| Age (years) | 2.6 (2.0; 3.1), <0.0001 |  |
| Sex | -17.6 (-24.3; -10.4), <0.0001 |  |
| log(CAC_b_+1) | -4.9 (-6.7; -3.1), <0.0001 |  |

GRS: genetic risk score, CAC: coronary artery calcification, LDL: low-density lipoprotein, HDL: high-density lipoprotein and EV: explained variance in percent compared to the model without GRS adjusted for age, sex and log(CAC_b_+1). The association between the genetic risk scores and outcome was carried out using linear regression in SAS. The model is adjusted for age, sex and log(CAC_b_+1). We subtracted the explained variance of the baseline model i.e. adjusted for age, sex and log(CAC_b_+1) to estimate the explained variance because of the genetic risk score.
