# Supplementary material for: Genetic risk scores for coronary artery disease and its traditional risk factors: Their role in the progression of coronary artery calcification—Results of the Heinz Nixdorf Recall study
Source: PLoS One. 2020 May 7;15(5):e0232735. doi: 10.1371/journal.pone.0232735 (PMC7205301; doi:10.1371/journal.pone.0232735)
Supplement: S2 Table — a) Association of coronary artery diseases-associated SNPs with log(obs)–log(exp) and the 5-year progression of CAC in the Heinz Nixdorf Recall study. b) Association of coronary artery calcification-associated SNPs with log(obs)–log(exp) and the 5-year progression of CAC in the Heinz Nixdorf Recall study. c) Association of diabetes-associated SNPs with log(obs)–log(exp) and the 5-year progression of CAC in the Heinz Nixdorf Recall study. d) Association of body mass index-associated SNPs with log(obs)–log(exp) and the 5-year progression of CAC in the Heinz Nixdorf Recall study. e) Association of systolic blood pressure-associated SNPs with log(obs)–log(exp) and the 5-year progression of CAC in the Heinz Nixdorf Recall study. f) Association of diastolic blood pressure-associated SNPs with log(obs)–log(exp) and the 5-year progression of CAC in the Heinz Nixdorf Recall study. g) Association of pulse pressure-associated SNPs with log(obs)–log(exp) and the 5-year progression of CAC in the Heinz Nixdorf Recall study. h) Association of low-density lipoprotein-cholesterol-associated SNPs with log(obs)–log(exp) and the 5-year progression of CAC in the Heinz Nixdorf Recall study. i) Association of high-density lipoprotein-cholesterol-associated SNPs with log(obs)–log(exp) and the 5-year progression of CAC in the Heinz Nixdorf Recall study. j) Association of triglyceride-associated SNPs with log(obs)–log(exp) and the 5-year progression of CAC in the Heinz Nixdorf Recall study. k) Association of total cholesterol-associated SNPs with log(obs)–log(exp) and the 5-year progression of CAC in the Heinz Nixdorf Recall study. CHR: chromosome, BP: base position (hgBuild37), CA: coded allele, NCA: non coded allele, CAF: coded allele frequency, 95%CI: 95% confidence interval, CAC: coronary artery calcification, “log(obs)–log(exp)”: percent deviation from the expected (CAC5y+1). The association between each SNP and outcomes was carried out using linear regression in PLINK. The models are a [file pone.0232735.s002.docx]

**Table S2(a).** Association of coronary artery diseases-associated SNPs with log(obs)–log(exp) and the 5-year progression of CAC in the Heinz Nixdorf Recall study

| CHR | SNP | Gene(s) in/nearby region | BP | CA | NCA | CAF | log(obs)–log(exp)  %(95%CI),p | 5-year progression of CAC  %(95%CI),p |
| --- | --- | --- | --- | --- | --- | --- | --- | --- |
| 1 | rs11206510 | *PCSK9* | 55496039 | T | C | 0.81 | 6.7( -1.2;15.2),0.1 | 4.9(-2.2;12.6),0.18 |
| 1 | rs17114036 | *PPAP2B* | 56962821 | A | G | 0.92 | 2.8( -8;14.9),0.62 | 2.6(-7.4;13.6),0.62 |
| 1 | rs599839 | *SORT1* | 109822166 | A | G | 0.76 | -0.4( -7.2;6.8),0.9 | 0.3(-6;6.9),0.94 |
| 1 | rs4845625 | *IL6R* | 154422067 | T | C | 0.44 | -2.2( -7.8;3.9),0.47 | -2.6(-7.8;3),0.36 |
| 1 | rs17464857 | *TAF1A* | 222762709 | T | G | 0.85 | -2.9( -10.6;5.4),0.48 | -3.3(-10.3;4.4),0.39 |
| 1 | rs17465637 | *MIA3* | 222823529 | C | A | 0.74 | 2.1( -4.7;9.3),0.56 | 1.4(-4.8;8),0.67 |
| 2 | rs515135 | *APOB* | 21286057 | C | T | 0.82 | 0.5( -6.9;8.5),0.9 | 0.1(-6.7;7.4),0.98 |
| 2 | rs6544713 | *ABCG5-ABCG8* | 44073881 | T | C | 0.31 | -1.7( -7.8;4.9),0.61 | -1.8(-7.4;4.2),0.55 |
| 2 | rs1561198 | *AMP5-VAMP8-GGC* | 85809989 | T | C | 0.44 | 4.1( -1.9;10.6),0.18 | 2.6(-2.9;8.4),0.36 |
| 2 | rs2252641 | *ZEB2-AC074093.1* | 145801461 | C | T | 0.44 | -2.8( -8.5;3.2),0.35 | -2.2(-7.5;3.3),0.42 |
| 2 | rs6725887 | *WDR12* | 203745885 | C | T | 0.13 | 0.4( -8.1;9.7),0.93 | 3.7(-4.4;12.6),0.38 |
| 2 | rs1801251 | *KCNJ13-*  *GIGYF2* | 233633460 | A | G | 0.33 | 5.3(-1.2;12.3),0.11 | 3.0(-0.8;11.5), 0.09 |
| 3 | rs9818870 | *MRAS* | 138122122 | T | C | 0.16 | -2.6( -10.1;5.6),0.53 | -2.6(-9.6;4.9),0.49 |
| 4 | rs17087335 | *NOA1* | 57838583 | T | G | 0.2 | 4.3( -3.1;12.2),0.26 | 4.2(-2.6;11.5),0.24 |
| 4 | rs1878406 | *EDNRA* | 148393664 | T | C | 0.14 | 5.9( -2.6;15.3),0.18 | 6.2(-1.7;14.8),0.13 |
| 4 | rs7692387 | *GUCY1A3* | 156635309 | G | A | 0.8 | 9.7( 1.9;18.2),0.01 | 10.3(3;18.1),0.005 |
| 5 | rs273909 | *SLC22A4-SLC22A5* | 131667353 | G | A | 0.12 | -4.6( -12.8;4.5),0.31 | -3.7(-11.4;4.6),0.37 |
| 6 | rs12526453 | *PHACTR1* | 12927544 | C | G | 0.66 | 2.0( -4.1;8.5),0.53 | 1.2(-4.4;7.2),0.68 |
| 6 | rs3130683 | *C2* | 31888367 | T | C | 0.86 | 4.4(-4.0;13.7),0.31 | 2.6(-5.3;10.0),0.50 |
| 6 | rs17609940 | *ANKS1A* | 35034800 | G | C | 0.81 | 3.0( -4.5;11.1),0.44 | 1.3(-5.5;8.6),0.72 |
| 6 | rs10947789 | *KCNK5* | 39174922 | T | C | 0.75 | -2.3( -8.8;4.6),0.5 | -3.6(-9.5;2.6),0.25 |
| 6 | rs12190287 | *TCF21* | 134214525 | C | G | 0.64 | -0.6( -6.5;5.7),0.86 | -0.4(-5.9;5.4),0.89 |
| 6 | rs2048327 | *SLC22A3* | 160863532 | C | T | 0.35 | -1.4( -7.4;4.9),0.65 | 0.6(-5;6.5),0.83 |
| 6 | rs3798220 | *SLC22A3-LPAL2-LPA* | 160961137 | C | T | 0.02 | 13.1( -10.8;43.4),0.31 | 14.1(-8.3;42),0.24 |
| 6 | rs4252120 | *PLG* | 161143608 | T | C | 0.7 | 1.5( -4.8;8.2),0.66 | 2.2(-3.7;8.4),0.48 |
| 7 | rs2023938 | *HDAC9* | 19036775 | C | T | 0.09 | 0.9( -9.1;12),0.86 | 0.8(-8.5;11),0.87 |
| 7 | rs12539895 | *7q22* | 107091849 | A | C | 0.2 | -6( -12.6;1.3),0.1 | -4.8(-11;1.9),0.16 |
| 7 | rs11556924 | *ZC3HC1* | 129663496 | C | T | 0.61 | 7( 0.6;13.7),0.03 | 4.7(-1;10.7),0.11 |
| 7 | rs3918226 | *NOS3* | 150690176 | T | C | 0.09 | 2.6( -7.7;14),0.64 | -0.7(-9.9;9.5),0.89 |
| 8 | rs264 | *LPL* | 19813180 | G | A | 0.86 | -4.5( -12.4;4),0.29 | -5.3(-12.5;2.5),0.18 |
| 8 | rs2954029 | [*AC091114.1*](https://www.ebi.ac.uk/gwas/genes/AC091114.1) | 126490972 | A | T | 0.51 | -0.4( -6.1;5.6),0.9 | -0.1(-5.4;5.5),0.97 |
| 9 | rs3217992 | *CDKN2A/B* | 22003223 | T | C | 0.37 | 6.6( 0.3;13.3),0.04 | 4.7(-1;10.8),0.11 |
| 9 | rs1333049 | *CDKN2A/B* | 22125503 | C | G | 0.45 | 7.1( 0.9;13.7),0.03 | 5.2(-0.5;11.2),0.07 |
| 9 | rs579459 | *ABO* | 136154168 | C | T | 0.24 | -1.3( -8;5.8),0.7 | -2.8(-8.9;3.6),0.38 |
| 10 | rs2505083 | *KIAA1462* | 30335122 | C | T | 0.44 | -1.3( -6.9;4.7),0.67 | -1.3(-6.5;4.2),0.64 |
| 10 | rs2047009 | CXCL12 | 44539913 | G | T | 0.51 | 7.5( 1.3;14.1),0.02 | 4.9(-0.7;10.8),0.09 |
| 10 | rs501120 | *CXCL12* | 44753867 | T | C | 0.86 | 16.0( 6.3;26.4),0.001 | 13.5(4.8;22.9),0.002 |
| 10 | rs11203042 | *LIPA* | 90989109 | T | C | 0.42 | -3.5( -9.2;2.5),0.25 | -4.2(-9.4;1.3),0.13 |
| 10 | rs2246833 | *LIPA* | 91005854 | T | C | 0.32 | -3.4( -9.3;2.8),0.28 | -3.2(-8.7;2.5),0.27 |
| 10 | rs12413409 | *CYP17A1-CNNM2-NT5C2* | 104719096 | G | A | 0.9 | 12.9( 2.4;24.5),0.01 | 11.2(1.6;21.6),0.02 |
| 11 | rs10840293 | *SWAP70* | 9751196 | A | G | 0.57 | 1.6( -4.4;8),0.61 | 0.5(-5.1;6.3),0.87 |
| 11 | rs974819 | *PDGFD* | 103660567 | T | C | 0.28 | 5.4( -1.3;12.5),0.12 | 4.9(-1.2;11.4),0.12 |
| 11 | rs11042937 | *MRVI1-CTR9* | 10745394 | T | G | 0.45 | -2.7(-8.2;3.2),0.36 | -1.3(-6.5;4.2),0.64 |
| 11 | rs9326246 | *ZNF259-APOA5-APOA1* | 116611733 | C | G | 0.07 | 0( -10.8;12.1),1 | -2.8(-12.5;8),0.6 |
| 11 | rs964184 | *APOA1-C3-A4-A5* | 116648917 | G | C | 0.14 | -1.8( -9.7;6.8),0.67 | -2.4(-9.7;5.5),0.54 |
| 12 | rs11172113 | *LRP1* | 57527283 | C | T | 0.40 | -0.9(-6.7;5.2),0.77 | -0.4(-5.75;5.3),0.89 |
| 12 | rs3184504 | *SH2B3* | 111884608 | T | C | 0.52 | -1.4( -7.1;4.7),0.64 | -1.9(-7.2;3.6),0.49 |
| 12 | rs11830157 | *KSR2* | 118265441 | G | T | 0.41 | 3.4( -3;10.2),0.3 | 4.4(-1.5;10.7),0.15 |
| 12 | rs11057830 | *SCARB1* | 125307053 | A | G | 0.14 | 5.2(-3.42;14.6),0.24 | 7.0(-1.12;15.8),0.09 |
| 13 | rs9319428 | *FLT1* | 28973621 | A | G | 0.31 | 0.3( -5.9;6.9),0.92 | -0.9(-6.5;5.2),0.78 |
| 13 | rs4773144 | *COL4A1-COL4A2* | 110960712 | G | A | 0.44 | 4.2( -1.9;10.6),0.18 | 4.3(-1.3;10.3),0.13 |
| 13 | rs9515203 | *COL4A1-COL4A2* | 111049623 | T | C | 0.73 | 2.4( -4.3;9.6),0.49 | 2.7(-3.5;9.3),0.4 |
| 14 | rs2895811 | *HHIPL1* | 100133942 | C | T | 0.43 | 0.9( -5;7.2),0.76 | 1.4(-4.1;7.3),0.62 |
| 15 | rs56062135 | *SMAD3* | 67455630 | C | T | 0.76 | 2.6( -4.3;10.1),0.47 | 0.9(-5.4;7.6),0.79 |
| 15 | rs3825807 | *ADAMTS7* | 79089111 | A | G | 0.55 | 1.2( -4.6;7.4),0.68 | 1.3(-4.1;7),0.65 |
| 15 | rs7173743 | *ADAMTS7* | 79141784 | T | C | 0.55 | 1.5( -4.4;7.7),0.63 | 1.8(-3.7;7.5),0.53 |
| 15 | rs8042271 | *MFGE8-ABHD2* | 89574218 | A | G | 0.05 | 4.1( -9.7;19.9),0.58 | -4(-15.8;9.3),0.54 |
| 15 | rs17514846 | *FURN-FES* | 91416550 | A | C | 0.48 | 5.9( -0.3;12.4),0.06 | 4(-1.6;9.9),0.17 |
| 16 | rs1800775 | *CETP* | 56995236 | C | A | 0.52 | -1.36(-7.0;4.6),0.65 | -2.0(-7.2;3.4),0.46 |
| 17 | rs216172 | *SMG6* | 2126504 | C | G | 0.34 | 2.3( -4;9),0.49 | 2.2(-3.6;8.4),0.46 |
| 17 | rs12936587 | *RAI1-PEMT-RASD1* | 17543722 | G | A | 0.55 | 3.6( -2.4;9.9),0.25 | 3.2(-2.3;9),0.27 |
| 17 | rs46522 | *UBE2Z* | 46988597 | C | T | 0.46 | -3.6( -9.1;2.3),0.23 | -3.2(-9.0;2.2),0.25 |
| 17 | rs7212798 | *BCAS3* | 59013488 | C | T | 0.16 | 2.4( -5.6;11),0.57 | 1.9(-5.5;9.8),0.63 |
| 18 | rs663129 | *PMAIP1-MC4R* | 57838401 | A | G | 0.23 | 2.7( -4.3;10.1),0.46 | 1.8(-4.5;8.6),0.58 |
| 19 | rs1122608 | *LDLR* | 11163601 | G | T | 0.75 | 3.5( -3.4;10.8),0.33 | 2(-4.2;8.7),0.53 |
| 19 | rs12976411 | *ZNF507-LOC400684* | 32882020 | A | T | 0.97 | -5.8( -22.2;13.9),0.54 | -4.9(-25;12),0.59 |
| 19 | rs2075650 | *TOMM40/ApoE-ApoC1* | 45395619 | G | A | 0.15 | 3.9( -4.4;12.9),0.37 | 1.9(-5.6;10.1),0.62 |
| 19 | rs445925 | *ApoE-ApoC1* | 45415640 | G | A | 0.88 | 6.5( -2.8;16.6),0.17 | 4.1(-4.3;13.2),0.35 |
| 21 | rs9982601 | *MRPS6* | 35599128 | T | C | 0.14 | 1.4( -6.9;10.5),0.74 | 0.3(-7.3;8.5),0.94 |
| 22 | rs180803 | *POMI21L9P-ADORA2A* | 24658858 | A | C | 0.99 | 25.8( -8.3;72.6),0.15 | 27.3(-4.8;70.2),0.1 |

**Table S2(b).** Association of coronary artery calcification-associated SNPs with log(obs)–log(exp) and the 5-year progression of CAC in the Heinz Nixdorf Recall study

| CHR | SNP | Gene(s) in/nearby region | BP | CA | NCA | CAF | log(obs)–log(exp)  %(95%CI),p | 5-year progression of CAC  %(95%CI), p |
| --- | --- | --- | --- | --- | --- | --- | --- | --- |
| 6 | rs9349379 | *PHACTR1* | 12903957 | G | A | 0.39 | 3.9(-2.2;10.4),0.21 | 2.4(-3.1; 8.3),0.40 |
| 9 | rs10965219 | *CDKN2A/B* | 22053687 | G | A | 0.47 | 6.9(0.7;13.5),0.03 | 4.9(-0.7; 10.9),0.09 |
| 9 | rs1333049 | *CDKN2A/B* | 22125503 | C | G | 0.45 | 7.1(0.9;13.7),0.03 | 5.2(-0.5; 11.2),0.07 |

**Table S2(c).** Association of diabetes-associated SNPs with log(obs)–log(exp) and the 5-year progression of CAC in the Heinz Nixdorf Recall study

| CHR | SNP | Gene(s) in/nearby region | BP | CA | NCA | CAF | log(obs)–log(exp)  %(95%CI),p | 5-year progression of CAC  %(95%CI),p |
| --- | --- | --- | --- | --- | --- | --- | --- | --- |
| 1 | rs17106184 | *FAF1* | 50909985 | G | A | 0.91 | -4.5(-13.9;5.9),0.38 | -3.6(-12.4;6.1),0.45 |
| 1 | rs10923931 | *NOTCH2* | 120517959 | T | G | 0.1 | 6.2(-3.6;17.1),0.23 | 6.2(-2.9;16.2),0.19 |
| 1 | rs2075423 | *PROX1* | 214154719 | G | T | 0.64 | 1.3(-4.8;7.7),0.68 | 0.8(-4.8;6.7),0.79 |
| 2 | rs2867125 | *TMEM18* | 622827 | C | T | 0.82 | 3.8(-3.9;12.1),0.35 | 3.6(-3.5;11.2),0.33 |
| 2 | rs780094 | *GCKR* | 27741237 | C | T | 0.6 | 2.1(-3.8;8.4),0.49 | 1.7(-3.7;7.5),0.54 |
| 2 | rs10203174 | *THADA* | 43690030 | C | T | 0.88 | -3.9(-12.3;5.4),0.4 | -4.9(-12.6;3.5),0.25 |
| 2 | rs243088 | *BCL11A* | 60568745 | T | A | 0.46 | 2.8(-3.1;9),0.36 | 1.7(-3.6;7.3),0.54 |
| 2 | rs11123406 | *BCL2L11* | 111950541 | T | C | 0.35 | 5(-1.4;11.9),0.13 | 4.4(-1.5;10.6),0.15 |
| 2 | rs998451 | *TMEM163* | 135429288 | A | G | 0.41 | -5.8(-11.2;0),0.05 | -3.9(-9;1.5),0.16 |
| 2 | rs4410242 | *RBMS1* | 161192070 | G | A | 0.81 | 0.6(-6.8;8.5),0.88 | 0.2(-6.5;7.5),0.94 |
| 2 | rs3923113 | *GRB14* | 165501849 | A | C | 0.61 | -6.4(-11.9;-0.5),0.03 | -4.1(-9.3;1.4),0.14 |
| 2 | rs2943640 | *IRS1* | 227093585 | C | A | 0.64 | 2.1(-3.9;8.5),0.5 | 0.8(-4.7;6.7),0.77 |
| 3 | rs1801282 | *PPARG* | 12393125 | C | G | 0.85 | 7.3(-1.2;16.6),0.1 | 5(-2.7;13.3),0.21 |
| 3 | rs7612463 | *UBE2E2* | 23336450 | C | A | 0.9 | -3.5(-12.5;6.5),0.48 | -0.6(-9.2;8.7),0.89 |
| 3 | rs831571 | *PSMD6* | 64048297 | C | T | 0.81 | -2.7(-9.9;5),0.48 | -2.5(-9.1;4.6),0.48 |
| 3 | rs6795735 | *ADAMTS9* | 64705365 | C | T | 0.59 | -1.7(-7.5;4.4),0.58 | -3(-8.2;2.6),0.29 |
| 3 | rs11717195 | *ADCY5* | 123082398 | T | C | 0.78 | -1.2(-8;6.2),0.74 | -1.4(-7.7;5.3),0.67 |
| 3 | rs4402960 | *IGF2BP2* | 185511687 | T | G | 0.31 | 0.9(-5.5;7.6),0.8 | 2.4(-3.5;8.7),0.43 |
| 3 | rs16861329 | *ST64GAL1* | 186666461 | C | T | 0.87 | 1.2(-7.4;10.6),0.79 | 1.5(-6.4;10.2),0.71 |
| 3 | rs6808574 | *LPP* | 187740523 | C | T | 0.61 | 4.9(-1.4;11.6),0.13 | 4.3(-1.5;10.5),0.15 |
| 4 | rs4458523 | *WFS1* | 6289986 | G | T | 0.59 | 3(-3.1;9.4),0.35 | 3(-2.6;8.9),0.3 |
| 4 | rs7674212 | *CISD2* | 103988899 | G | T | 0.58 | -6.8(-12.2;-1),0.02 | -5.8(-10.9;-0.4),0.04 |
| 4 | rs2706785 | *TMEM155* | 122660250 | G | A | 0.03 | -1.4(-16.6;16.7),0.87 | -4.6(-18.3;11.4),0.55 |
| 4 | rs6813195 | *TMEM154* | 153520475 | C | T | 0.73 | -0.6(-7.1;6.2),0.85 | -2.5(-8.3;3.7),0.42 |
| 4 | rs1996546 | *ACSL1* | 185714289 | G | T | 0.85 | 0.3(-7.8;9.1),0.94 | 0.4(-7.1;8.5),0.92 |
| 5 | rs702634 | *ARL15* | 53271420 | A | G | 0.68 | 0.2(-5.9;6.6),0.96 | -0.1(-5.7;5.8),0.97 |
| 5 | rs459193 | *ANKRD55* | 55806751 | G | A | 0.73 | 3(-3.6;10.2),0.38 | 1.8(-4.3;8.3),0.57 |
| 5 | rs6878122 | *ZBED3* | 76427311 | G | A | 0.29 | -2.6(-8.7;4),0.44 | -3.4(-9.1;2.6),0.26 |
| 5 | rs329122 | *PHF15* | 133864599 | A | G | 0.41 | 2(-4;8.4),0.52 | 2.3(-3.3;8.2),0.43 |
| 6 | rs9505118 | *SSR1/RREB1* | 7290437 | A | G | 0.6 | 1.6(-4.3;7.8),0.6 | 1.6(-3.8;7.4),0.57 |
| 6 | rs7756992 | *CDKAL1* | 20679709 | G | A | 0.29 | 5.1(-1.7;12.3),0.14 | 5.4(-0.8;12),0.09 |
| 6 | rs3130501 | *POU5F1/TCF19* | 31136453 | G | A | 0.73 | -2.8(-9;3.7),0.39 | -2.2(-7.9;3.8),0.46 |
| 6 | rs2050188 | *HLA-DRB5* | 32339897 | T | C | 0.62 | 1.9(-4.3;8.4),0.56 | 2.2(-3.5;8.2),0.46 |
| 6 | rs9271775 | *HLA-DQA1* | 32594328 | T | C | 0.82 | 7.1(-0.7;15.5),0.08 | 4.7(-2.3;12.3),0.19 |
| 6 | rs9470794 | *ZFAND3* | 38106844 | T | C | 0.92 | 8.1(-2.6;17.7),0.13 | 7.8(-2.1;16.7),0.12 |
| 6 | rs4407733 | *IL20RA* | 137299152 | A | G | 0.53 | -3.4(-9;2.5),0.25 | -3.3(-8.5;2.1),0.23 |
| 6 | rs622217 | *SLC22A3* | 160766770 | T | C | 0.48 | 2.4(-3.6;8.7),0.44 | 2.8(-2.7;8.7),0.32 |
| 7 | rs17168486 | *DGKB* | 14898282 | T | C | 0.18 | -7(-13.9;0.4),0.06 | -7.7(-14;-0.9),0.03 |
| 7 | rs849135 | *JAZF1* | 28196413 | G | A | 0.5 | 4.4(-1.6;10.8),0.15 | 3.5(-2;9.3),0.22 |
| 7 | rs10278336 | *GCK* | 44245363 | A | G | 0.57 | -0.6(-6.5;5.7),0.86 | 0.8(-4.8;6.7),0.79 |
| 7 | rs6467136 | *GCC1* | 127164958 | A | G | 0.45 | -2(-8.2;3.9),0.51 | -1.5(-7.1;3.9),0.6 |
| 7 | rs13233731 | *KLF14* | 130437689 | G | A | 0.51 | -1.5(-7.1;4.5),0.61 | 0.3(-5;5.8),0.92 |
| 7 | rs9648716 | *BRAF* | 140612163 | T | A | 0.14 | -0.3(-8.5;8.6),0.94 | 1.8(-5.9;10.1),0.66 |
| 7 | rs1182397 | *MNX1* | 157031407 | G | T | 0.84 | 3.7(-4.4;12.5),0.39 | 4.2(-3.3;12.3),0.28 |
| 8 | rs12681990 | *KCNU1* | 36859186 | C | T | 0.19 | 0.8(-6.7;8.8),0.85 | 1.1(-5.8;8.5),0.76 |
| 8 | rs516946 | *ANK1* | 41519248 | C | T | 0.76 | 0.6(-6;7.7),0.85 | 0.7(-5.5;7.2),0.84 |
| 8 | rs7845219 | *TP53INP1* | 95937502 | T | C | 0.54 | -1.1(-6.8;4.9),0.71 | -1.3(-6.6;4.2),0.63 |
| 8 | rs3802177 | *SLC30A8* | 118185025 | G | A | 0.69 | 1.3(-5;8.1),0.69 | 1.8(-4;8.1),0.55 |
| 9 | rs7041847 | *GLIS3* | 4287466 | A | G | 0.51 | -0.7(-6.4;5.4),0.82 | -1.7(-7;3.8),0.53 |
| 9 | rs17584499 | *PTPRD* | 8879118 | T | C | 0.2 | 0(-7.1;7.7),0.99 | -1.9(-8.3;5),0.59 |
| 9 | rs10811661 | *CDKN2A/B* | 22134094 | T | C | 0.83 | 1.5(-6.2;9.9),0.7 | 1.1(-6;8.7),0.78 |
| 9 | rs17791513 | *TLE4* | 81905590 | A | G | 0.93 | 1.3(-10.2;14.2),0.84 | 1.6(-9.1;13.5),0.78 |
| 9 | rs2796441 | *TLE1* | 84308948 | G | A | 0.6 | 0.8(-5.1;7.1),0.8 | 0.8(-4.7;6.5),0.78 |
| 9 | rs495828 | *ABO* | 136154867 | T | G | 0.24 | -0.7(-7.2;6.3),0.85 | -2.3(-8.2;4),0.47 |
| 10 | rs11257655 | *CDC123* | 12307894 | T | C | 0.21 | -0.2(-7.3;7.5),0.97 | -1.1(-7.6;5.9),0.76 |
| 10 | rs1802295 | *VPS26A* | 70931474 | T | C | 0.32 | 3.3(-2.9;10),0.3 | 2.7(-3;8.8),0.36 |
| 10 | rs12571751 | *ZMIZ1* | 80942631 | A | G | 0.53 | 4.2(-1.7;10.5),0.17 | 3.1(-2.4;8.8),0.28 |
| 10 | rs1111875 | *HHEX/IDE* | 94462882 | C | T | 0.59 | -3.2(-8.9;2.8),0.29 | -3.4(-8.7;2.1),0.22 |
| 10 | rs7903146 | *TCF7L2* | 114758349 | T | C | 0.27 | 0.6(-5.9;7.5),0.87 | 0.1(-5.8;6.5),0.97 |
| 10 | rs10886471 | *GRK5* | 121149403 | T | C | 0.45 | 0.3(-5.6;6.5),0.92 | 0.7(-4.7;6.5),0.8 |
| 10 | rs2421016 | *PLEKHA1* | 124167512 | C | T | 0.5 | -2.7(-8.3;3.2),0.36 | -2.6(-7.7;2.8),0.34 |
| 11 | rs2334499 | *DUSP8* | 1696849 | T | C | 0.4 | 3.5(-2.6;10),0.26 | 1.6(-4;7.4),0.58 |
| 11 | rs163184 | *KCNQ1* | 2847069 | G | T | 0.5 | -4.4(-9.9;1.4),0.13 | -3.6(-8.7;1.7),0.18 |
| 11 | rs5215 | *KCNJ11* | 17408630 | C | T | 0.37 | 0.1(-5.8;6.5),0.96 | -0.3(-5.8;5.5),0.92 |
| 11 | rs3736505 | *HSD17B12* | 43876435 | G | A | 0.3 | -3.4(-9.4;3),0.29 | -4.3(-9.8;1.6),0.15 |
| 11 | rs11227234 | *MAP3K11* | 65365171 | T | G | 0.24 | 3(-4;10.5),0.41 | 3.2(-3.3;10.1),0.34 |
| 11 | rs1552224 | *ARAP1 (CENTD2)* | 72433098 | A | C | 0.84 | -11.2(-18.1;-3.9),0.004 | -9.3(-15.8;-2.4),0.01 |
| 11 | rs10830963 | *MTNR1B* | 92708710 | G | C | 0.29 | 7.1(0.2;14.4),0.04 | 6.8(0.5;13.5),0.03 |
| 12 | rs11063069 | *CCND2* | 4374373 | G | A | 0.21 | -0.5(-7.5;7),0.9 | 0.6(-5.9;7.6),0.85 |
| 12 | rs10842994 | *KLHDC5* | 27965150 | C | T | 0.79 | -2(-8.9;5.5),0.6 | -0.2(-6.7;6.8),0.95 |
| 12 | rs2261181 | *HMGA2* | 66212318 | T | C | 0.09 | -4.3(-13.8;6.2),0.41 | -4.9(-13.6;4.7),0.31 |
| 12 | rs7955901 | *TSPAN8* | 71433293 | C | T | 0.45 | 6.1(-0.1;12.6),0.05 | 4.6(-1;10.6),0.11 |
| 12 | rs12427353 | *HNF1B* | 121426901 | G | C | 0.82 | 2.3(-5.2;10.4),0.56 | 1.7(-5.2;9),0.65 |
| 12 | rs1727294 | *MPHOSPH9* | 123616514 | G | A | 0.79 | -6(-12.6;1.1),0.09 | -5.4(-11.5;1.2),0.11 |
| 12 | rs825476 | *CCDC92* | 124568456 | T | C | 0.57 | 3.2(-2.8;9.5),0.31 | 4.2(-1.3;10.1),0.14 |
| 13 | rs10507349 | *RNF6* | 26781528 | G | A | 0.77 | -3.2(-9.8;3.9),0.37 | -2.9(-9.1;3.6),0.37 |
| 13 | rs576674 | *KL* | 33554302 | G | A | 0.17 | 5.9(-2.2;14.6),0.16 | 3.5(-3.8;11.3),0.36 |
| 13 | rs1359790 | *SPRY2* | 80717156 | G | A | 0.72 | 6.7(-0.1;14),0.06 | 4.3(-1.9;10.8),0.18 |
| 13 | rs7985179 | *MIR17HG* | 91940169 | T | A | 0.75 | -5.3(-11.6;1.5),0.12 | -6.4(-12.2;-0.2),0.04 |
| 14 | rs17109256 | *NRXN3* | 79939993 | A | G | 0.23 | -3.7(-10.3;3.3),0.29 | -3.7(-9.8;2.7),0.25 |
| 15 | rs7403531 | *RASGRP1* | 38822905 | T | C | 0.21 | 0.2(-6.9;7.8),0.97 | 0.2(-6.4;7.2),0.96 |
| 15 | rs4502156 | *C2CD4A* | 62383155 | C | T | 0.41 | -0.6(-6.9;5.3),0.85 | 0.1(-5.7;5.5),0.98 |
| 15 | rs7178572 | *HMG20A* | 77747190 | G | A | 0.7 | -1.1(-7.3;5.6),0.74 | 0.7(-5.2;6.9),0.82 |
| 15 | rs11634397 | *ZFAND6* | 80432222 | G | A | 0.68 | -3.4(-9.3;2.8),0.28 | -2.7(-8.2;3.1),0.35 |
| 15 | rs2028299 | *AP3S2* | 90374257 | C | A | 0.27 | 6(-0.8;13.3),0.08 | 4.6(-1.6;11.2),0.15 |
| 15 | rs12899811 | *PRC1* | 91544076 | G | A | 0.31 | 0.6(-5.6;7.3),0.85 | 1.8(-4;8),0.56 |
| 16 | rs9940149 | *ITFG3* | 300641 | G | A | 0.81 | -2.2(-9.3;5.5),0.57 | -2.5(-9.1;4.5),0.47 |
| 16 | rs9936385 | *FTO* | 53819169 | C | T | 0.41 | 0.2(-5.7;6.4),0.95 | 0.5(-5;6.3),0.86 |
| 16 | rs7202877 | *BCAR1* | 75247245 | T | G | 0.89 | -6.6(-15;2.6),0.15 | -4.7(-12.7;3.9),0.27 |
| 16 | rs2925979 | *CMIP* | 81534790 | T | C | 0.3 | 6.7(0;13.7),0.05 | 7.4(1.2;14),0.02 |
| 17 | rs391300 | *SRR* | 2216258 | C | T | 0.64 | 0.1(-6;6.5),0.99 | 0.3(-5.3;6.2),0.91 |
| 17 | rs8068804 | *ZZEF1* | 3985864 | A | G | 0.32 | -1.3(-7.3;5.1),0.68 | 0(-5.6;6),0.99 |
| 17 | rs17676067 | *GLP2R* | 9791375 | C | T | 0.28 | -3.6(-9.7;2.9),0.27 | -4.4(-9.9;1.5),0.14 |
| 17 | rs11651052 | *HNF1B* | 36102381 | G | A | 0.53 | 2.4(-3.5;8.6),0.44 | 3.9(-1.6;9.7),0.17 |
| 17 | rs15563 | *GIP* | 47005193 | G | A | 0.54 | 3.4(-2.6;9.7),0.27 | 2.9(-2.6;8.6),0.31 |
| 18 | rs12970134 | *MC4R* | 57884750 | A | G | 0.26 | 5.1(-1.7;12.4),0.15 | 4(-2.3;10.6),0.22 |
| 19 | rs10401969 | *CILP2* | 19407718 | C | T | 0.08 | -2.7(-13.1;8.9),0.63 | 0.7(-9.2;11.7),0.9 |
| 19 | rs3786897 | *PEPD* | 33893008 | A | G | 0.58 | 5.4(-0.7;11.8),0.08 | 3.5(-2;9.3),0.22 |
| 19 | rs8108269 | *GIPR* | 46158513 | G | T | 0.31 | -1.1(-7.2;5.4),0.72 | -1.6(-7.2;4.3),0.59 |
| 20 | rs4812829 | *HNF4A* | 42989267 | A | G | 0.18 | 0.5(-7;8.6),0.91 | 1.1(-5.9;8.6),0.76 |

**Table S2(d).** Association of body mass index-associated SNPs with log(obs)–log(exp) and the 5-year progression of CAC in the Heinz Nixdorf Recall study

| CHR | SNP | Gene(s) in/nearby region | BP | CA | NCA | CAF | log(obs)–log(exp)  %(95%CI),p | 5-year progression of CAC  %(95%CI),p |
| --- | --- | --- | --- | --- | --- | --- | --- | --- |
| 1 | rs977747 | *TAL1* | 47684677 | T | G | 0.38 | -5.5(-11.2;0.6),0.08 | -3.5(-8.9;2.2),0.22 |
| 1 | rs657452 | *AGBL4* | 49589847 | A | G | 0.39 | -1.7(-7.5;4.4),0.57 | -1.2(-6.5;4.5),0.68 |
| 1 | rs11583200 | *ELAVL4* | 50559820 | C | T | 0.38 | -0.6(-6.4;5.5),0.83 | -0.6(-5.9;5.1),0.84 |
| 1 | rs3101336 | *NEGR1* | 72751185 | C | T | 0.62 | -4.6(-10.3;1.4),0.13 | -5.1(-10.3;0.4),0.07 |
| 1 | rs12566985 | *FPGT-TNNI3K* | 75002193 | G | A | 0.42 | -3.3(-8.9;2.8),0.28 | -1.1(-6.5;4.5),0.69 |
| 1 | rs12401738 | *FUBP1* | 78446761 | A | G | 0.35 | -5(-10.7;1),0.1 | -4(-9.3;1.6),0.15 |
| 1 | rs11165643 | *PTBP2* | 96924097 | T | C | 0.58 | -3.3(-8.9;2.6),0.26 | -2.3(-7.5;3.3),0.41 |
| 1 | rs17024393 | *GNAT2* | 110154688 | C | T | 0.04 | -3.9(-17.4;11.7),0.6 | -8(-19.9;5.8),0.24 |
| 1 | rs543874 | *SEC16B* | 177889480 | G | A | 0.18 | -1.7(-9;6.1),0.65 | -3.4(-10;3.7),0.34 |
| 1 | rs2820292 | *NAV1* | 201784287 | C | A | 0.56 | -0.4(-6.1;5.6),0.89 | 0.1(-5.2;5.7),0.98 |
| 2 | rs13021737 | *TMEM18* | 632348 | G | A | 0.82 | 2.6(-5;10.9),0.51 | 2.4(-4.7;9.9),0.52 |
| 2 | rs10182181 | *ADCY3* | 25150296 | G | A | 0.45 | 1.3(-4.5;7.5),0.67 | 1.1(-4.2;6.8),0.68 |
| 2 | rs11126666 | *KCNK3* | 26928811 | A | G | 0.27 | -2.9(-9.2;3.9),0.4 | -1.7(-7.6;4.5),0.58 |
| 2 | rs1016287 | *LINC01122* | 59305625 | T | C | 0.27 | 5.1(-1.7;12.4),0.14 | 3.8(-2.5;10.4),0.24 |
| 2 | rs11688816 | *EHBP1* | 63053048 | G | A | 0.53 | 5.6(-0.6;12.2),0.08 | 5.2(-0.5;11.2),0.07 |
| 2 | rs2121279 | *LRP1B* | 143043285 | T | C | 0.14 | 5.1(-3.4;14.4),0.25 | 3.2(-4.5;11.6),0.43 |
| 2 | rs1460676 | *FIGN* | 164567689 | C | T | 0.17 | -0.4(-8;7.9),0.92 | -0.8(-7.9;6.8),0.83 |
| 2 | rs1528435 | *UBE2E3* | 181550962 | T | C | 0.63 | 5.7(-0.6;12.3),0.08 | 4.3(-1.4;10.3),0.14 |
| 2 | rs17203016 | *CREB1* | 208255518 | G | A | 0.2 | -1.7(-8.6;5.8),0.65 | -0.1(-6.6;6.8),0.97 |
| 2 | rs7599312 | *ERBB4* | 213413231 | G | A | 0.72 | -1.1(-7.4;5.6),0.74 | -0.9(-6.7;5.2),0.76 |
| 2 | rs492400 | *USP37* | 219349752 | C | T | 0.41 | 2(-4.1;8.4),0.53 | 2(-3.6;7.9),0.48 |
| 2 | rs2176040 | *LOC646736* | 227092802 | A | G | 0.36 | -2.4(-8.2;3.7),0.44 | -1.1(-6.5;4.6),0.71 |
| 3 | rs6804842 | *RARB* | 25106437 | G | A | 0.59 | 0.3(-5.5;6.5),0.92 | 1.3(-4.1;7.1),0.64 |
| 3 | rs2365389 | *FHIT* | 61236462 | C | T | 0.59 | -2.1(-7.9;3.9),0.48 | -1.4(-6.8;4.2),0.62 |
| 3 | rs3849570 | *GBE1* | 81792112 | A | C | 0.34 | -3.9(-9.6;2.2),0.2 | -3(-8.4;2.6),0.28 |
| 3 | rs13078960 | *CADM2* | 85807590 | G | T | 0.19 | 1(-6.3;9),0.79 | 2.3(-4.6;9.7),0.52 |
| 3 | rs2035935 | *RASA2* | 141306013 | G | A | 0.06 | 1.6(-9.8;14.6),0.79 | 0.7(-9.9;12.4),0.91 |
| 3 | rs1516725 | *E7V5* | 185824004 | C | T | 0.87 | -4.8(-12.8;3.9),0.27 | -4.5(-11.9;3.5),0.26 |
| 4 | rs10938397 | *GNPDA2* | 45182527 | G | A | 0.43 | -1.1(-6.8;4.9),0.71 | -0.5(-5.7;5.1),0.87 |
| 4 | rs17001561 | *SCARB2* | 77096118 | A | G | 0.15 | -2.8(-10.5;5.5),0.49 | -3.8(-10.8;3.7),0.31 |
| 4 | rs13107325 | *SLC39A8* | 103188709 | T | C | 0.06 | -5(-16.1;7.6),0.42 | -6.4(-16.5;4.9),0.26 |
| 4 | rs11727676 | *HHIP* | 145659064 | T | C | 0.91 | -4.1(-13.5;6.4),0.43 | -4.6(-13.3;4.9),0.33 |
| 5 | rs2112347 | *POC5* | 75015242 | T | G | 0.63 | -1.9(-7.8;4.3),0.54 | -2.2(-7.6;3.6),0.45 |
| 5 | rs7715256 | *GALNT10* | 153537893 | G | T | 0.42 | 10.9(4.5;17.7),0.0007 | 11(5.1;17.2),0.0002 |
| 6 | rs205262 | *C6orf106* | 34563164 | G | A | 0.28 | -3.5(-9.6;3.1),0.29 | -1.9(-7.7;4.2),0.53 |
| 6 | rs2033529 | *TDRG1* | 40348653 | G | A | 0.29 | -0.2(-6.5;6.5),0.96 | -0.4(-6.2;5.8),0.9 |
| 6 | rs2207139 | *TFAP2B* | 50845490 | G | A | 0.18 | 2.9(-4.7;11.1),0.47 | 3.3(-3.7;10.9),0.36 |
| 6 | rs9400239 | *FOXO3* | 108977663 | C | T | 0.7 | -1.4(-7.5;5.2),0.68 | -0.3(-6;5.8),0.93 |
| 6 | rs9374842 | *LOC285762* | 120185665 | T | C | 0.75 | 5.2(-1.8;12.6),0.15 | 4(-2.4;10.8),0.22 |
| 6 | rs13201877 | *IFNGR1* | 137675541 | G | A | 0.15 | -0.1(-7.9;8.4),0.98 | -0.3(-7.6;7.5),0.93 |
| 6 | rs13191362 | *PARK2* | 163033350 | A | G | 0.9 | -7.2(-15.8;2.4),0.14 | -4.6(-12.9;4.4),0.3 |
| 7 | rs1167827 | *HIP1* | 75163169 | G | A | 0.57 | -3.9(-9.4;2),0.19 | -2.6(-7.8;2.9),0.34 |
| 7 | rs2245368 | *PMS2L11* | 76608143 | C | T | 0.16 | 2.1(-5.7;10.7),0.6 | -0.1(-7.2;7.6),0.99 |
| 7 | rs9641123 | *CALCR* | 93197732 | C | G | 0.4 | 0.4(-5.7;6.8),0.91 | 1(-4.6;7),0.73 |
| 7 | rs6465468 | *ASB4* | 95169514 | T | G | 0.31 | -0.5(-6.6;6.1),0.89 | -0.5(-6.2;5.5),0.86 |
| 8 | rs17405819 | *HNF4G* | 76806584 | T | C | 0.71 | -2.7(-8.9;3.8),0.4 | -1.7(-7.4;4.4),0.58 |
| 8 | rs16907751 | *ZBTB10* | 81375457 | C | T | 0.89 | 4.6(-5.1;15.2),0.37 | 4.7(-4.2;14.5),0.31 |
| 8 | rs2033732 | *RALYL* | 85079709 | C | T | 0.74 | 1.4(-5.1;8.3),0.69 | -1.4(-7.2;4.8),0.65 |
| 9 | rs4740619 | *C9orf93* | 15634326 | T | C | 0.57 | -1.3(-7;4.8),0.67 | -0.5(-5.9;5.1),0.85 |
| 9 | rs10968576 | *LINGO2* | 28414339 | G | A | 0.31 | 1.3(-5;8),0.69 | -0.5(-6.2;5.6),0.87 |
| 9 | rs6477694 | *EPB41L4B* | 111932342 | C | T | 0.38 | -2(-7.8;4.1),0.51 | -1.2(-6.6;4.5),0.68 |
| 9 | rs1928295 | *TLR4* | 120378483 | T | C | 0.53 | -0.9(-6.5;5.1),0.77 | -0.1(-5.4;5.4),0.96 |
| 9 | rs10733682 | *LMX1B* | 129460914 | A | G | 0.5 | 2.5(-3.4;8.8),0.41 | 2.2(-3.2;7.9),0.43 |
| 10 | rs7899106 | *GRID1* | 87410904 | G | A | 0.04 | -3.5(-16.5;11.4),0.62 | -5.5(-17.3;7.9),0.4 |
| 10 | rs17094222 | *HIF1AN* | 102395440 | C | T | 0.2 | 3.4(-4.1;11.5),0.39 | 3.3(-3.6;10.7),0.36 |
| 10 | rs11191560 | *NT5C2* | 104869038 | C | T | 0.1 | -11.7(-19.9;-2.6),0.01 | -10.6(-18.2;2.2),0.01 |
| 10 | rs7903146 | *TCF7L2* | 114758349 | C | T | 0.73 | -0.6(-7;6.3),0.87 | -0.1(-6.1;6.2),0.97 |
| 11 | rs4256980 | *TRIM66* | 8673939 | G | C | 0.64 | -3.8(-9.5;2.2),0.21 | -3(-8.3;2.7),0.29 |
| 11 | rs11030104 | *BDAF* | 27684517 | A | G | 0.78 | 2.1(-5;9.8),0.57 | 2.8(-3.8;9.9),0.41 |
| 11 | rs2176598 | *HSD17B12* | 43864278 | T | C | 0.24 | -3.1(-9.4;3.8),0.37 | -3.5(-9.4;2.7),0.26 |
| 11 | rs3817334 | *MTCH2* | 47650993 | T | C | 0.41 | 2.5(-3.5;8.9),0.42 | 2.3(-3.3;8.1),0.43 |
| 11 | rs12286929 | *CADM1* | 115022404 | G | A | 0.55 | -3(-8.7;3),0.32 | -3.4(-8.7;2.1),0.22 |
| 12 | rs7138803 | *BCDIN3D* | 50247468 | A | G | 0.4 | 0.2(-5.7;6.5),0.94 | 1.3(-4.2;7.1),0.65 |
| 12 | rs11057405 | *CLIP1* | 122781897 | G | A | 0.89 | -6.2(-14.7;3.1),0.18 | -5.4(-13.3;3.3),0.22 |
| 13 | rs9581855 | *MTIF3* | 28018014 | A | G | 0.2 | -4.5(-11.2;2.8),0.22 | -3.3(-9.6;3.4),0.32 |
| 13 | rs12429545 | *OLFM4* | 54102206 | A | G | 0.13 | -1.7(-10.3;7.7),0.71 | 0(-8.1;8.8),1 |
| 13 | rs9540493 | *MIR548X2* | 66205704 | A | G | 0.43 | -6(-11.4;-0.3),0.04 | -6.6(-11.6;-1.3),0.02 |
| 13 | rs1441264 | *MIR548A2* | 79580919 | A | G | 0.6 | 1.5(-4.5;7.9),0.63 | 2.2(-3.4;8.1),0.46 |
| 14 | rs10132280 | *STXBP6* | 25928179 | C | A | 0.7 | -0.5(-6.7;6.3),0.89 | -2.9(-8.5;3.2),0.34 |
| 14 | rs12885454 | *PRKD1* | 29736838 | C | A | 0.66 | -1.3(-7.2;5.1),0.69 | -1.3(-6.8;4.5),0.64 |
| 14 | rs11847697 | *PRKD1* | 30515112 | T | C | 0.04 | 8.9(-6.2;26.4),0.26 | 9.8(-4.3;25.9),0.18 |
| 14 | rs7141420 | *NRXN3* | 79899454 | T | C | 0.53 | 1.7(-4.2;8),0.58 | 0.4(-5;6.1),0.89 |
| 15 | rs3736485 | *DMXL2* | 51748610 | A | G | 0.43 | -6.2(-11.6;-0.5),0.03 | -5.1(-10.1;0.3),0.06 |
| 15 | rs16951275 | *M4P2K5* | 68077168 | T | C | 0.77 | 0.5(-6.3;7.8),0.89 | 1.1(-5.2;7.8),0.75 |
| 15 | rs7164727 | *LOC100287559* | 73093991 | T | C | 0.69 | -5.5(-11.4;0.7),0.08 | -5(-10.4;0.8),0.09 |
| 16 | rs758747 | *NLRC3* | 3627358 | T | C | 0.27 | -2.3(-8.7;4.6),0.5 | -0.7(-6.7;5.7),0.82 |
| 16 | rs12446632 | *GPRC5B* | 19935389 | G | A | 0.85 | 7.4(-1.2;16.7),0.1 | 6.7(-1.2;15.3),0.1 |
| 16 | rs2650492 | *SBK1* | 28333411 | A | G | 0.33 | 3.1(-3.2;9.9),0.34 | 2.2(-3.6;8.3),0.46 |
| 16 | rs3888190 | *ATP2A1* | 28889486 | A | C | 0.4 | 0.5(-5.4;6.8),0.86 | 0(-5.4;5.8),0.99 |
| 16 | rs4787491 | *INO80E* | 30015337 | G | A | 0.53 | -5.6(-11.1;0.2),0.06 | -4.3(-9.4;1.2),0.12 |
| 16 | rs9925964 | *KAT8* | 31129895 | A | G | 0.62 | 0.3(-5.8;6.7),0.93 | 0.3(-5.3;6.2),0.92 |
| 16 | rs2080454 | *CBLN1* | 49062590 | C | A | 0.38 | -1.3(-7.1;4.9),0.67 | -1(-6.3;4.7),0.74 |
| 16 | rs1558902 | *FTO* | 53803574 | A | T | 0.42 | 1.1(-4.7;7.4),0.71 | 1.5(-4;7.3),0.6 |
| 17 | rs9914578 | *SMG6* | 2005136 | G | C | 0.2 | -0.8(-7.9;6.8),0.82 | 1.3(-5.4;8.4),0.72 |
| 17 | rs1000940 | *RABEP1* | 5283252 | G | A | 0.29 | -1.2(-7.5;5.6),0.72 | -2.4(-8.2;3.8),0.44 |
| 17 | rs12940622 | *RPTOR* | 78615571 | G | A | 0.55 | 3.2(-2.7;9.6),0.29 | 3.3(-2.2;9.1),0.24 |
| 18 | rs1808579 | *NPC1* | 21104888 | C | T | 0.53 | 1.9(-4;8.1),0.54 | 0.9(-4.5;6.5),0.75 |
| 18 | rs7239883 | *LOC284260* | 40147671 | G | A | 0.4 | -4.3(-10;1.7),0.15 | -2.8(-8.1;2.8),0.32 |
| 18 | rs7243357 | *GRP* | 56883319 | T | G | 0.83 | -5(-12.3;3),0.21 | -5(-11.8;2.3),0.17 |
| 18 | rs6567160 | *MC4R* | 57829135 | C | T | 0.23 | 2.9(-4;10.4),0.42 | 2.2(-4.2;9),0.52 |
| 19 | rs17724992 | *PGPEP1* | 18454825 | A | G | 0.75 | 0.3(-6.3;7.3),0.93 | 0.7(-5.4;7.2),0.83 |
| 19 | rs29941 | *KCTD15* | 34309532 | G | A | 0.69 | -2.4(-8.4;4),0.46 | -2.1(-7.7;3.8),0.47 |
| 19 | rs2075650 | *TOMM40/ ApoE-ApoC1* | 45395619 | A | G | 0.85 | -3.7(-11.4;4.6),0.37 | -1.9(-9.2;5.9),0.62 |
| 19 | rs2287019 | *QPCTL* | 46202172 | C | T | 0.79 | 5.5(-1.9;13.5),0.15 | 5.7(-1.2;13),0.11 |
| 19 | rs3810291 | *ZC3H4* | 47569003 | A | G | 0.68 | 4.3(-2.1;11.1),0.19 | 3.6(-2.2;9.9),0.23 |
| 20 | rs6091540 | *ZFP64* | 51087862 | C | T | 0.75 | -4.4(-10.8;2.4),0.2 | -2.9(-8.9;3.4),0.36 |
| 21 | rs2836754 | *ETS2* | 40291740 | C | T | 0.63 | -2.4(-8.2;3.7),0.43 | -2.3(-7.6;3.4),0.43 |

**Table S2(e).** Association of systolic blood pressure-associated SNPs with log(obs)–log(exp) and the 5-year progression of CAC in the Heinz Nixdorf Recall study

| CHR | SNP | Gene(s) in/nearby region | BP | CA | NCA | CAF | log(obs)–log(exp)  %(95%CI),p | 5-year progression of CAC  %(95%CI),p |
| --- | --- | --- | --- | --- | --- | --- | --- | --- |
| 1 | rs17367504 | *CLCN6* | 11862778 | A | G | 0.85 | 1.6(-6.5;10.4), 0.72 | 3.4(-4.2;11.7),0.39 |
| 1 | rs3820068 | *CELA2A* | 15798197 | A | G | 0.8 | -2.6(-10.2;5.6), 0.52 | -5.2(-12;2.1),0.16 |
| 1 | rs10922502 | *GTF2B* | 89360158 | G | A | 0.38 | -4.4(-10.3;2), 0.18 | -3.4(-9;2.4),0.25 |
| 1 | rs2932538 | *MOV10* | 113216543 | G | A | 0.73 | 0.6(-5.9;7.6), 0.85 | 0.7(-5.3;7.1),0.82 |
| 2 | rs7562 | *FOSL2* | 28635740 | T | C | 0.56 | 1.7(-4.2;8), 0.57 | 1.7(-3.8;7.4),0.56 |
| 2 | rs13420463 | *PRKD3* | 37517566 | A | G | 0.79 | -2.5(-9.8;5.5), 0.53 | -3.2(-9.9;4),0.37 |
| 3 | rs9859176 | *RYK* | 134000025 | T | C | 0.41 | -3.8(-9.9;2.8), 0.26 | -2.5(-8.3;3.6),0.42 |
| 3 | rs419076 | *MECOM* | 169100886 | T | C | 0.46 | -0.5(-6.7;6.1), 0.88 | -0.3(-6;5.8),0.93 |
| 4 | rs1458038 | *FGF5* | 81164723 | T | C | 0.3 | 0.8(-5.5;7.6), 0.8 | -0.2(-5.9;6),0.96 |
| 4 | rs13107325 | *SLC39A8* | 103188709 | C | T | 0.94 | 5.2(-7;19.1), 0.42 | 6.8(-4.7;19.8),0.26 |
| 4 | rs13112725 | *NPNT* | 106911742 | C | G | 0.77 | -3.4(-10.5;4.4), 0.38 | -2.9(-9.5;4.2),0.41 |
| 5 | rs1173771 | *NPR3-C5orf23* | 32815028 | G | A | 0.58 | 1.5(-4.4;7.7), 0.64 | -0.4(-5.7;5.2),0.88 |
| 5 | rs10059921 | *TMEM161B* | 87514515 | G | T | 0.83 | -0.1(-13.6;11.9), 0.99 | 0.8(-11.5;11.7),0.89 |
| 5 | rs6595838 | *FBN2* | 127868199 | A | G | 0.28 | 0.8(-6.1;8.3), 0.82 | 1.6(-4.8;8.5),0.64 |
| 5 | rs11953630 | *FBF1* | 157845402 | C | T | 0.62 | 2.7(-3.4;9.2), 0.39 | 3.6(-2;9.6),0.21 |
| 6 | rs6911827 | *CASC15* | 22130601 | T | C | 0.46 | 1.2(-4.8;7.7), 0.7 | 1.7(-3.9;7.7),0.55 |
| 6 | rs1799945 | *HFE* | 26091179 | G | C | 0.15 | 7.7(-0.8;17), 0.08 | 6.3(-1.4;14.8),0.11 |
| 6 | rs805303 | *BAT2-BAT5* | 31616366 | G | A | 0.62 | -1.2(-7.1;5.1), 0.71 | 0.1(-5.4;6),0.97 |
| 6 | rs78648104 | *TFAP2D* | 50683009 | C | T | 0.07 | 6.6(-4.9;19.6), 0.27 | 4.8(-5.7;16.5),0.38 |
| 7 | rs13238550 | *MKLN1* | 131059056 | A | G | 0.39 | 3.9(-2.6;10.9), 0.24 | 4.5(-1.6;10.9),0.15 |
| 7 | rs1011018 | *HIPK2* | 139463264 | G | A | 0.80 | -5.9(-14.6;2.2), 0.16 | -3.7(-11.5;3.6),0.33 |
| 8 | rs894344 | *ZFAT* | 135612745 | G | A | 0.39 | 0.1(-5.9;6.4), 0.98 | 1(-4.6;6.9),0.74 |
| 10 | rs4373814 | *CACNB2* | 18419972 | C | G | 0.43 | -0.1(-6.2;6.4), 0.98 | -0.5(-6.4;5.2),0.88 |
| 10 | rs1813353 | *CACNB2* | 18707448 | T | C | 0.64 | 0(-6.4;6.8), 1 | 0.3(-5.6;6.6),0.92 |
| 10 | rs4590817 | *C10orf107* | 63467553 | G | C | 0.85 | 5.9(-2.4;15), 0.17 | 6(-1.7;14.4),0.13 |
| 10 | rs932764 | *PLCE1* | 95895940 | G | A | 0.45 | 1.7(-4.2;7.9), 0.58 | 2.3(-3.2;8),0.42 |
| 10 | rs112184198 | *PAX2* | 102604514 | G | A | 0.9 | 11.7(0.5;24.1), 0.04 | -8.6(-19.6;1.4),0.09 |
| 10 | rs11191548 | *CYP17A1-NT5C2* | 104846178 | T | C | 0.9 | 13.7(3.2;25.4), 0.01 | 11.8(2.2;22.3),0.02 |
| 11 | rs7129220 | *ADM* | 10350538 | A | G | 0.11 | 2(-7.3;12.1), 0.69 | 0.7(-7.7;9.9),0.87 |
| 11 | rs381815 | *PLEKHA7* | 16902268 | T | C | 0.28 | 0.5(-5.9;7.3), 0.88 | 1.4(-4.6;7.7),0.66 |
| 11 | rs633185 | *FLJ32810-TMEM133* | 100593538 | C | G | 0.71 | -3.5(-9.5;3), 0.28 | 4.4(-1.4;9.9),0.13 |
| 12 | rs6487543 | *SSPN* | 26438189 | A | G | 0.76 | -2(-14.1;11.8), 0.76 | -0.1(-11.3;12.5),0.99 |
| 12 | rs17249754 | *ATP2B1* | 90060586 | G | A | 0.85 | -2.3(-10.8;6.9), 0.61 | -0.9(-8.8;7.6),0.82 |
| 12 | rs3184504 | *SH2B3* | 111884608 | T | C | 0.52 | -1.4(-7.1;4.7), 0.64 | -1.9(-7.2;3.6),0.49 |
| 12 | rs10850411 | *TBX5-TBX3* | 115387796 | T | C | 0.71 | -3.9(-9.9;2.6), 0.24 | -3.8(-9.3;2.2),0.21 |
| 13 | rs9549328 | *MCF2L* | 113636156 | T | C | 0.22 | -3.8(-10.9;3.9), 0.32 | -4.7(-11.1;2.3),0.19 |
| 14 | rs9888615 | *FERMT2* | 53377540 | C | T | 0.7 | -2.3(-9.1;4.9), 0.51 | -2.4(-8.6;4.2),0.46 |
| 14 | rs8016306 | *PPP2R5E* | 63928546 | A | G | 0.78 | -4(-11.1;3.6), 0.29 | -3.1(-9.7;3.9),0.38 |
| 15 | rs1378942 | *CYP1A2* | 75077367 | C | A | 0.34 | -0.5(-6.6;5.9), 0.87 | -0.2(-5.8;5.8),0.96 |
| 15 | rs35199222 | *ABHD17C* | 81013037 | A | G | 0.44 | 1.3(-5;7.9), 0.7 | 0.5(-5.2;6.6),0.86 |
| 15 | rs2521501 | *FURIN-FES* | 91437388 | T | A | 0.35 | 4.4(-1.9;11.2), 0.17 | 3.6(-2.2;9.8),0.23 |
| 16 | rs11643209 | *CFDP1* | 75331044 | G | T | 0.57 | -0.5(-7.4;5.9), 0.87 | -0.9(-7.2;5.1),0.77 |
| 17 | rs12941318 | *CRK* | 1333598 | C | T | 0.51 | -0.4(-6.9;6.6), 0.91 | 0.2(-5.8;6.7),0.94 |
| 17 | rs12946454 | *PLCD3* | 43208121 | T | A | 0.26 | -1.6(-8.1;5.3), 0.64 | -3.5(-9.4;2.8),0.27 |
| 17 | rs17608766 | *GOSR2* | 45013271 | C | T | 0.13 | 7.2(-1.7;17), 0.12 | 7.3(-1;16.2),0.09 |
| 17 | rs12940887 | *ZNF652* | 47402807 | T | C | 0.39 | 1.8(-4.2;8.2), 0.56 | 1.1(-4.4;6.9),0.7 |
| 17 | rs2467099 | *ACOX1* | 73949045 | C | T | 0.79 | -3.9(-11;3.7), 0.3 | -4.1(-10.6;2.8),0.24 |
| 20 | rs1327235 | *JAG1* | 10969030 | G | A | 0.46 | -0.6(-6.3;5.4), 0.84 | 0.2(-5.1;5.8),0.93 |
| 20 | rs6015450 | *GNAS-EDN3* | 57751117 | G | A | 0.14 | 0.4(-7.8;9.4), 0.92 | -1.3(-8.8;6.8),0.75 |

**Table S2(f)**: Association of diastolic blood pressure-associated SNPs with log(obs)–log(exp) and the 5-year progression of CAC in the Heinz Nixdorf Recall study

| CHR | SNP | Gene(s) in/nearby region | BP | CA | NCA | CAF | log(obs)–log(exp)  %(95%CI),p | 5-year progression of CAC  %(95%CI),p |
| --- | --- | --- | --- | --- | --- | --- | --- | --- |
| 1 | rs17367504 | *CLCN6* | 11862778 | A | G | 0.85 | 1.6(-6.5;10.4),0.72 | 3.4(-4.2;11.7),0.39 |
| 1 | rs6686889 | *AL445648.1* | 25030470 | T | C | 0.25 | 0.8(-6;8),0.83 | 0.9(-5.4;7.5),0.79 |
| 1 | rs2932538 | *MOV10* | 113216543 | G | A | 0.73 | 0.6(-5.9;7.6),0.85 | 0.7(-5.3;7.1),0.82 |
| 1 | rs12405515 | *DNM3* | 172357441 | G | T | 0.42 | 1.5(-4.4;7.9),0.62 | 1.5(-4;7.3),0.59 |
| 1 | rs12408022 | *GPATCH2* | 217718789 | T | C | 0.24 | -1.1(-7.7;6.1),0.77 | -2.3(-8.4;4.2),0.48 |
| 1 | rs10916082 | *CDC42BPA* | 227252626 | G | A | 0.26 | -0.4(-6.9;6.6),0.91 | -0.3(-6.3;6.2),0.94 |
| 1 | rs2760061 | *WNT3A* | 228191075 | A | T | 0.5 | -2.7(-8.4;3.4),0.37 | -2.2(-7.5;3.4),0.44 |
| 1 | rs953492 | *SDCCAG8* | 243471192 | A | G | 0.5 | 4.6(-1.5;11.1),0.14 | 3.4(-2.1;9.3),0.23 |
| 2 | rs55701159 | *ADCY3* | 25139596 | T | G | 0.9 | 2.1(-7.4;12.6),0.67 | 3.4(-5.5;13.1),0.47 |
| 2 | rs4952611 | *SLC8A1* | 40567743 | C | T | 0.4 | 4.3(-2.2;11.2),0.2 | 4(-2;10.4),0.19 |
| 2 | rs76326501 | *ACO16735.1* | 43167878 | A | C | 0.93 | -2.5(-13.1;9.3),0.66 | -1.5(-11.3;9.5),0.78 |
| 2 | rs2579519 | *GPAT2-FAHD2CP* | 96675166 | C | T | 0.39 | -2.9(-8.7;3.3),0.35 | -2.3(-7.7;3.4),0.41 |
| 2 | rs1438896 | *TEX41* | 145646072 | T | C | 0.31 | -3.6(-9.7;2.8),0.26 | -4.3(-9.9;1.5),0.14 |
| 2 | rs79146658 | *CCDC141* | 179786068 | C | T | 0.09 | -10.2(-19.2;-0.2),0.05 | -8.6(-17.1;0.7),0.07 |
| 2 | rs7592578 | *TMEM194B* | 191439591 | G | T | 0.83 | -5.8(-12.8;1.7),0.13 | -6.7(-13.1;0.2),0.06 |
| 2 | rs1063281 | *TNS1* | 218668732 | C | T | 0.4 | 5.8(-0.5;12.4),0.07 | 5.9(0.1;12),0.05 |
| 3 | rs13082711 | *SLC4A7* | 27537909 | C | T | 0.22 | 3(-4.2;10.7),0.43 | 2.2(-4.3;9.3),0.51 |
| 3 | rs3774372 | *ULK4* | 41877414 | C | T | 0.16 | 1.9(-5.9;10.4),0.65 | 1.1(-6.1;8.8),0.78 |
| 3 | rs36022378 | *CAMKV-ACTBP13* | 49913705 | C | T | 0.2 | -2.1(-9.3;5.7),0.59 | -2(-8.6;5.1),0.58 |
| 3 | rs743757 | *CACNA2D2* | 50476378 | C | G | 0.14 | -1.3(-9.3;7.3),0.75 | -0.6(-8;7.4),0.88 |
| 3 | rs9827472 | *FAM208A* | 56726646 | C | T | 0.66 | 2.9(-3.4;9.6),0.38 | 2.4(-3.4;8.5),0.43 |
| 3 | rs2306374 | *MRAS* | 138119952 | C | T | 0.17 | -7.8(-20.6;7),0.28 | -4.1(-10.9;3.2),0.27 |
| 3 | rs143112823 | *RP11-439C8.2* | 154707967 | G | A | 0.93 | 2.2(-9.2;14.9),0.72 | 0.5(-9.9;12),0.93 |
| 3 | rs419076 | *MECOM* | 169100886 | T | C | 0.46 | -1.8(-7.5;4.3),0.56 | -1.2(-6.5;4.4),0.67 |
| 3 | rs12374077 | *SENP2* | 185317674 | C | G | 0.36 | -2.1(-8;4.1),0.49 | -2.8(-8.2;2.9),0.32 |
| 4 | rs1458038 | *FGF5* | 81164723 | T | C | 0.3 | 0.8(-5.5;7.6),0.8 | -0.2(-5.9;6),0.96 |
| 4 | rs13107325 | *SLC39A8* | 103188709 | C | T | 0.94 | 5.2(-7;19.1),0.42 | 6.8(-4.7;19.8),0.26 |
| 4 | rs66887589 | *PDE5A* | 120509279 | C | T | 0.48 | -2.5(-8.1;3.5),0.41 | -2.4(-7.6;3.1),0.39 |
| 4 | rs13139571 | *GUCY1A3-GUCY1B3* | 156645513 | C | A | 0.76 | 9.6(2.3;17.4),0.01 | 9.6(2.9;16.8),0.01 |
| 5 | rs1173771 | *NPR3-C5orf23* | 32815028 | G | A | 0.58 | 1.5(-4.4;7.7),0.64 | -0.4(-5.7;5.2),0.88 |
| 5 | rs10078021 | *SLC25A5P9* | 75038431 | G | T | 0.38 | 5.1(-1.3;12),0.12 | 4.9(-1;11.2),0.1 |
| 5 | rs11953630 | *FBF1* | 157845402 | C | T | 0.62 | 2.7(-3.4;9.2),0.39 | 3.6(-2;9.6),0.21 |
| 5 | rs72812846 | *CPEB4* | 173377636 | T | A | 0.72 | -4.2(-10.5;2.6),0.22 | -3.9(-9.7;2.3),0.21 |
| 6 | rs1799945 | *HFE* | 26091179 | G | C | 0.15 | 7.7(-0.8;17),0.08 | 6.3(-1.4;14.8),0.11 |
| 6 | rs805303 | *BAT2-BAT5* | 31616366 | G | A | 0.62 | -1.2(-7.1;5.1),0.71 | 0.1(-5.4;6),0.97 |
| 6 | rs13205180 | *PKHD1* | 51832494 | T | C | 0.49 | 0.6(-5.3;6.8),0.86 | 0.5(-4.9;6.3),0.85 |
| 6 | rs9372498 | *SLC35F1* | 118572486 | A | T | 0.09 | -0.5(-10.5;10.8),0.93 | -2.8(-11.9;7.2),0.57 |
| 6 | rs147212971 | *PDE10A* | 166178451 | C | T | 0.94 | -1.6(-13.8;12.4),0.81 | -1.3(-12.7;11.6),0.84 |
| 8 | rs2978098 | *SNX31* | 101676675 | A | C | 0.55 | -3.9(-9.5;2),0.19 | -3.8(-8.9;1.7),0.17 |
| 8 | rs62524579 | *RP11-273G15.2* | 144060955 | G | A | 0.45 | -2.1(-7.8;4),0.49 | -2.6(-7.8;2.9),0.35 |
| 9 | rs4364717 | *MTAP* | 21801530 | G | A | 0.46 | -0.9(-6.6;5.2),0.76 | -0.8(-6.1;4.8),0.78 |
| 10 | rs4373814 | *CACNB2* | 18419972 | C | G | 0.44 | -0.5(-6.2;5.6),0.88 | 0(-5.6;5.4),0.99 |
| 10 | rs1813353 | *CACNB2* | 18707448 | T | C | 0.63 | -1.5(-7.4;4.8),0.63 | -1.1(-6.6;4.7),0.7 |
| 10 | rs11191548 | *CYP17A1-NT5C2* | 104846178 | T | C | 0.9 | 13.7(3.2;25.4),0.01 | 11.8(2.2;22.3),0.02 |
| 11 | rs7129220 | *ADM* | 10350538 | A | G | 0.11 | 2(-7.3;12.1),0.69 | 0.7(-7.7;9.9),0.87 |
| 11 | rs381815 | *PLEKHA7* | 16902268 | T | C | 0.28 | 0.5(-5.9;7.3),0.88 | 1.4(-4.6;7.7),0.66 |
| 11 | rs11030119 | *BDNF* | 27728102 | G | A | 0.73 | -1(-7.3;5.8),0.77 | -0.2(-6.1;6),0.94 |
| 11 | rs67330701 | *MYEOV* | 69079707 | C | T | 0.91 | 3.1(-8.6;16.2),0.62 | 2.4(-8.3;14.3),0.68 |
| 11 | rs633185 | *FLJ32810-TMEM133* | 100593538 | C | G | 0.71 | -3.5(-9.5;3),0.28 | 4.4(-1.4;9.9),0.13 |
| 12 | rs17249754 | *ATP2B1* | 90060586 | G | A | 0.86 | -2.5(-10.4;6.2),0.57 | -1.5(-8.9;6.5),0.71 |
| 12 | rs3184504 | *SH2B3* | 111884608 | T | C | 0.52 | -1.4(-7.1;4.7),0.64 | -1.9(-7.2;3.6),0.49 |
| 12 | rs10850411 | *TBX5-TBX3* | 115387796 | T | C | 0.71 | -3.9(-9.9;2.6),0.24 | -3.8(-9.3;2.2),0.21 |
| 15 | rs7178615 | *RP11-321F6.1* | 66869072 | G | A | 0.61 | -0.1(-5.9;6.1),0.97 | 0.7(-4.8;6.4),0.82 |
| 15 | rs1378942 | *CYP1A2* | 75077367 | C | A | 0.34 | -0.5(-6.6;5.9),0.87 | -0.2(-5.8;5.8),0.96 |
| 15 | rs62012628 | *ADAMTS7* | 79070000 | C | T | 0.72 | -2(-8.7;5.1),0.57 | -2.6(-8.7;3.9),0.42 |
| 15 | rs2521501 | *FURIN-FES* | 91437388 | T | A | 0.35 | 4.4(-1.9;11.2),0.17 | 3.6(-2.2;9.8),0.23 |
| 15 | rs12906962 | *AC009432.1* | 95312071 | C | T | 0.32 | -3.3(-9.3;3.1),0.31 | -2.7(-8.3;3.2),0.36 |
| 16 | rs12921187 | *PPL* | 4943019 | G | T | 0.58 | 4.1(-1.9;10.5),0.19 | 3.7(-1.8;9.6),0.19 |
| 16 | rs72799341 | *FBXL19* | 30936743 | A | G | 0.25 | -6.6(-12.8;-0.1),0.05 | -5(-10.8;1.1),0.1 |
| 16 | rs8059962 | *CMIP* | 81574197 | C | T | 0.6 | 0.3(-5.8;6.8),0.92 | 0.5(-5.1;6.5),0.87 |
| 17 | rs12940887 | *ZNF652* | 47402807 | T | C | 0.39 | 1.8(-4.2;8.2),0.56 | 1.1(-4.4;6.9),0.7 |
| 17 | rs4308 | *ACE* | 61559625 | A | G | 0.37 | 5.9(-0.6;12.8),0.08 | 5.7(-0.3;12.1),0.06 |
| 18 | rs745821 | *MAPK4* | 48142854 | T | G | 0.74 | -6.9(-13.1;-0.4),0.04 | -5.6(-11.3;0.5),0.07 |
| 19 | rs62104477 | *CCNE1* | 30294991 | T | G | 0.33 | -5.5(-11.3;0.8),0.08 | -4.4(-9.9;1.4),0.13 |
| 20 | rs6108168 | *PLCB1* | 8626271 | C | A | 0.74 | 1.7(-4.9;8.8),0.62 | 2.8(-3.4;9.4),0.39 |
| 20 | rs1327235 | *JAG1* | 10969030 | G | A | 0.46 | -0.6(-6.3;5.4),0.84 | 0.2(-5.1;5.8),0.93 |
| 20 | rs6015450 | *GNAS-EDN3* | 57751117 | G | A | 0.14 | 0.4(-7.8;9.4),0.92 | -1.3(-8.8;6.8),0.75 |

**Table S2(g).** Association of pulse pressure-associated SNPs with log(obs)–log(exp) and the 5-year progression of CAC in the Heinz Nixdorf Recall study

| CHR | SNP | Gene(s) in/nearby region | BP | CA | NCA | CAF | log(obs)–log(exp)  %(95%CI),p | 5-year progression of CAC  % (95%CI),p |
| --- | --- | --- | --- | --- | --- | --- | --- | --- |
| 1 | rs112557609 | *RP4-710M16.1-PPAP2B* | 56576924 | A | G | 0.33 | 1.9(-4.5;8.7),0.56 | 2.3(-3.6;8.6),0.45 |
| 1 | rs12731740 | *CD34* | 208024820 | C | T | 0.89 | 1.1(-8;11.1),0.82 | 1.6(-6.9;10.8),0.73 |
| 1 | rs3889199 | *FGGY* | 59653742 | A | G | 0.72 | 0.5(-6;7.4),0.89 | 0.4(-5.5;6.8),0.89 |
| 1 | rs4360494 | *SF3A3* | 38455891 | C | G | 0.56 | 3.6(-2.5;10.1),0.25 | 5(-0.7;11.1),0.09 |
| 1 | rs9662255 | *BX323043.1/SPSB1* | 9441949 | C | A | 0.58 | -6.1(-11.6;-0.3),0.04 | -5.9(-11;-0.5),0.03 |
| 2 | rs11689667 | *TCF7L1* | 85491365 | T | C | 0.56 | 3.6(-2.4;9.9),0.25 | 3.1(-2.4;8.9),0.27 |
| 2 | rs11690961 | *PRKCE* | 46363336 | A | C | 0.9 | 6.8(-3.4;18),0.2 | 6.9(-2.5;17.2),0.16 |
| 2 | rs1250259 | *FN1* | 216300482 | T | A | 0.27 | -0.4(-6.7;6.4),0.91 | -0.7(-6.6;5.5),0.82 |
| 2 | rs13002573 | *FIGN* | 164915208 | A | G | 0.79 | 5.7(-1.7;13.7),0.13 | 5.2(-1.6;12.5),0.14 |
| 2 | rs2289081 | *C2orf43* | 20881840 | G | C | 0.64 | -0.8(-6.8;5.6),0.81 | 0.2(-5.4;6.1),0.96 |
| 2 | rs3771371 | *ZNF638* | 71627539 | C | T | 0.41 | 1.7(-4.4;7.5),0.57 | 2.3(-3.3;7.6),0.41 |
| 2 | rs74181299 | *CEP68* | 65283972 | T | C | 0.61 | -1.5(-7.3;4.7),0.63 | -1.4(-6.8;4.2),0.61 |
| 3 | rs62270945 | *GATA2* | 128201889 | T | C | 0.02 | 4.2(-18.3;32.8),0.74 | -2.2(-21.7;22.2),0.85 |
| 4 | rs1566497 | *PALLD* | 169717148 | A | C | 0.42 | 1.6(-4.6;8.2),0.63 | 0.4(-5.3;6.3),0.9 |
| 4 | rs17059668 | *AC106895.1/HAND2-AS1* | 174584663 | G | C | 0.07 | 4.8(-6.9;17.9),0.44 | 3.2(-7.5;15.1),0.58 |
| 4 | rs871606 | *CHIC2* | 54799245 | T | C | 0.9 | -0.7(-10.2;9.9),0.89 | -1.9(-10.6;7.7),0.69 |
| 5 | rs10057188 | *LHFPL2* | 77837789 | G | A | 0.55 | 0.3(-5.7;6.6),0.93 | 0.8(-4.7;6.6),0.78 |
| 6 | rs11154027 | *GJA1* | 121781390 | T | C | 0.43 | -4.2(-9.9;1.8),0.17 | -2.6(-8;3),0.36 |
| 6 | rs1322639 | *THBS2* | 169587103 | A | G | 0.79 | -3.2(-10;4.1),0.38 | -1.4(-7.8;5.5),0.68 |
| 6 | rs449789 | *FNDC1* | 159699125 | C | G | 0.13 | -4.8(-12.8;4),0.27 | -4.3(-11.7;3.8),0.29 |
| 7 | rs17477177 | *PIK3CG* | 106411858 | C | T | 0.2 | 5.4(-2;13.3),0.16 | 3.6(-3.1;10.8),0.3 |
| 7 | rs76206723 | *SUGCT* | 40447971 | G | A | 0.89 | -7.3(-15.5;1.8),0.11 | -7(-14.7;1.3),0.1 |
| 8 | rs2071518 | *NOV* | 120435812 | T | C | 0.25 | 1.3(-5.4;8.5),0.71 | 2.7(-3.6;9.4),0.42 |
| 8 | rs2978456 | *SLC20A2* | 42324765 | C | T | 0.43 | -0.6(-7;6.3),0.87 | -0.5(-6.5;5.8),0.86 |
| 8 | rs4454254 | *TRAPPC9* | 141060027 | G | A | 0.37 | -1(-7;5.3),0.75 | -1.1(-6.6;4.7),0.71 |
| 9 | rs72765298 | *SCAI* | 127900996 | C | T | 0.1 | 5.5(-4.5;16.6),0.29 | 5(-4.2;15.2),0.3 |
| 10 | rs10826995 | *ARHGAP12* | 32082658 | C | T | 0.28 | -1.1(-7.6;5.8),0.75 | -0.7(-6.7;5.7),0.82 |
| 10 | rs9337951 | *KIAA1462* | 30317073 | A | G | 0.31 | 0(-7.3;7.8),0.99 | -1.5(-8;5.6),0.68 |
| 11 | rs11222084 | *ADAMTS8* | 130273230 | T | A | 0.36 | 0.6(-5.4;7.1),0.84 | 0.5(-5.1;6.4),0.87 |
| 11 | rs2289125 | *NOX4* | 89224453 | C | A | 0.79 | -2.7(-9.6;4.7),0.46 | -3.6(-9.9;3.1),0.29 |
| 11 | rs7126805 | *CRACR2B* | 828916 | A | G | 0.73 | 0.1(-6.3;7),0.97 | -0.2(-6.1;6),0.94 |
| 11 | rs8258 | *CEP164* | 117283676 | T | C | 0.35 | -6.3(-12;-0.3),0.04 | -5.2(-10.5;0.5),0.07 |
| 12 | rs139236208 | *CCDC41* | 94880742 | G | A | 0.93 | -2.4(-14.3;11.2),0.72 | -0.8(-12;11.8),0.89 |
| 14 | rs9323988 | *RP11-6101.1* | 98587630 | C | T | 0.38 | -1(-6.8;5.2),0.75 | -0.7(-6.1;5),0.82 |
| 16 | rs117006983 | *VAC14* | 70755610 | A | G | 0 | 2.6(-64;192.7),0.96 | 3.6(-60.4;171.3),0.94 |
| 16 | rs7500448 | *CDH13* | 83045790 | A | G | 0.75 | -1.3(-7.9;5.7),0.71 | -0.8(-7;5.7),0.79 |
| 17 | rs62080325 | *PYY* | 42060631 | G | A | 0.32 | 1(-5.4;8),0.76 | 1.6(-4.5;8),0.62 |
| 17 | rs7226020 | *KIAA0753* | 6473828 | C | T | 0.45 | 3.3(-2.8;9.8),0.29 | 2.9(-2.7;8.9),0.31 |
| 17 | rs740698 | *MRC2* | 60767151 | C | T | 0.42 | -2.2(-8.1;4.1),0.48 | -2.5(-7.9;3.3),0.39 |
| 17 | rs78378222 | *TP53-SLC2A4* | 7571752 | T | G | 0.99 | -8.1(-34.6;29.4),0.63 | -3.6(-29.5;32),0.82 |
| 17 | rs79089478 | *KCNH4-HSD17B1* | 40317241 | T | C | 0.97 | -14.2(-28.5;2.9),0.1 | -16.6(-29.5;-1.3),0.03 |
| 18 | rs7236548 | *SLC14A2* | 43097750 | A | C | 0.18 | -0.6(-7.9;7.4),0.89 | -0.7(-7.5;6.6),0.84 |
| 20 | rs6081613 | *SLC24A3* | 19465907 | A | G | 0.3 | 0.4(-5.9;7.2),0.89 | 1.3(-4.6;7.5),0.67 |
| 22 | rs12628032 | *ARVCF* | 19967980 | T | C | 0.32 | -0.6(-6.7;6),0.86 | -0.7(-6.4;5.3),0.81 |
| 22 | rs73161324 | *XRCC6* | 42038786 | T | C | 0.05 | -2.2(-16.2;14.2),0.78 | -0.8(-13.9;14.4),0.92 |

**Table S2(h).** Association of low-density lipoprotein-cholesterol associated SNPs with log(obs)–log(exp) and the 5-year progression of CAC in the Heinz Nixdorf Recall study

| CHR | SNP | Gene(s) in/nearby region | BP | CA | NCA | CAF | log(obs)–log(exp)  %(95%CI),p | 5-year progression of CAC  %(95%CI),p |
| --- | --- | --- | --- | --- | --- | --- | --- | --- |
| 1 | rs12027135 | *LDLRAP1* | 25775733 | T | A | 0.57 | -2.2(-7.8;3.8),0.47 | -0.3(-5.6;5.3),0.91 |
| 1 | rs12748152 | *PIGV-NROB2* | 27138393 | T | C | 0.08 | 3.3(-7.4;15.3),0.55 | 2.9(-7;13.8),0.58 |
| 1 | rs2479409 | *PCSK9* | 55504650 | G | A | 0.35 | 3.1(-3.1;9.8),0.33 | 1.1(-4.6;7.1),0.71 |
| 1 | rs2131925 | *ANGPTL3* | 63025942 | T | G | 0.68 | 2.5(-3.9;9.2),0.46 | 2.3(-3.6;8.5),0.45 |
| 1 | rs629301 | *SORT1* | 109818306 | T | G | 0.77 | 0.7(-6.2;8),0.85 | 1.2(-5.1;8),0.71 |
| 1 | rs267733 | *ANXA9-CERS2* | 150958836 | A | G | 0.85 | 6.2(-2.1;15.2),0.15 | 6.1(-1.6;14.3),0.12 |
| 1 | rs2807834 | *MOSC1* | 220970593 | G | T | 0.69 | 7(0.3;14.1),0.04 | 7.7(1.5;14.3),0.01 |
| 1 | rs514230 | *IRF2BP2* | 234858597 | T | A | 0.52 | 3.5(-2.5;9.9),0.26 | 3.3(-2.2;9.1),0.25 |
| 2 | rs1367117 | *APOB* | 21263900 | A | G | 0.32 | 1.6(-4.7;8.4),0.62 | 0.9(-4.9;7.1),0.76 |
| 2 | rs4299376 | *ABCG5/8* | 44072576 | G | T | 0.31 | -1.4(-7.5;5.2),0.67 | -1.9(-7.6;4.1),0.52 |
| 2 | rs2710642 | *EHBP1* | 63149557 | A | G | 0.66 | 3.6(-2.7;10.4),0.27 | 4(-1.8;10.2),0.18 |
| 2 | rs10490626 | *INSIG2* | 118835841 | G | A | 0.93 | -6.8(-17.1;4.8),0.24 | -4.5(-14.2;6.4),0.41 |
| 2 | rs2030746 | *LOC84931* | 121309488 | T | C | 0.41 | 2.1(-3.9;8.5),0.50 | 0.4(-5.1;6.2),0.9 |
| 2 | rs1250229 | *FN1* | 216304384 | C | T | 0.73 | 0.4(-6;7.2),0.91 | 0.6(-5.4;6.9),0.85 |
| 2 | rs11563251 | *UGT1A1* | 234679384 | T | C | 0.09 | -4.8(-14.1;5.6),0.35 | -1.6(-10.5;8.3),0.75 |
| 3 | rs7640978 | *CMTM6* | 32533010 | C | T | 0.92 | 2.4(-8.5;14.5),0.68 | 1.2(-8.7;12.2),0.81 |
| 3 | rs17404153 | *ACAD11* | 132163200 | G | T | 0.88 | 1.2(-7.7;10.8),0.81 | 2.4(-5.8;11.4),0.58 |
| 4 | rs6831256 | *LRPAP1* | 3473139 | A | G | 0.58 | -2.4(-8;3.5),0.41 | -2.3(-7.4;3.2),0.41 |
| 5 | rs12916 | *HMGCR* | 74656539 | C | T | 0.42 | 3.3(-2.8;9.8),0.30 | 4(-1.6;10),0.17 |
| 5 | rs4530754 | *CSNK1G3* | 122855416 | A | G | 0.57 | 4.4(-1.7;10.9),0.16 | 3.6(-2;9.5),0.21 |
| 5 | rs6882076 | *TIMD4* | 156390297 | C | T | 0.62 | 1.5(-4.5;7.8),0.63 | 1.9(-3.6;7.7),0.5 |
| 6 | rs3757354 | *MYLIP* | 16127407 | C | T | 0.78 | 6.7(-0.6;14.6),0.07 | 5.8(-0.9;13),0.09 |
| 6 | rs1800562 | *HFE* | 26093141 | G | A | 0.96 | 5.4(-8.6;21.6),0.47 | 5.3(-7.7;20),0.44 |
| 6 | rs3177928 | *HLA* | 32412435 | A | G | 0.14 | -1.2(-9.2;7.5),0.78 | 0.1(-7.4;8.2),0.99 |
| 6 | rs9488822 | *FRK* | 116312893 | T | A | 0.31 | -7.5(14.7;0.8),0.03 | -5.5(-12;0.6),0.08 |
| 6 | rs11153594 | *FRK* | 116354591 | C | T | 0.63 | 6.8(0.5;13.5),0.03 | 5.9(0.1;12),0.05 |
| 6 | rs1564348 | *LPA* | 160578860 | C | T | 0.17 | 0.1(-7.6;8.4),0.98 | 0.3(-6.8;8),0.94 |
| 7 | rs12670798 | *DNAH11* | 21607352 | C | T | 0.24 | 3.5(-3.5;11),0.34 | 2.4(-4;9.3),0.47 |
| 7 | rs4722551 | *MIR148A* | 25991826 | C | T | 0.16 | 3.7(-4.4;12.5),0.38 | 3.7(-3.8;11.8),0.34 |
| 8 | rs2126259 | *PPP1R3B* | 9185146 | C | T | 0.9 | 2.3(-7.5;13.3),0.66 | 1.8(-7.3;11.8),0.7 |
| 8 | rs10102164 | *SOX17* | 55421614 | A | G | 0.19 | -3.8(-10.8;3.7),0.31 | -4.5(-10.9;2.4),0.19 |
| 8 | rs1030431 | *CYP7A1* | 59311697 | A | G | 0.36 | -1(-7;5.4),0.76 | -2.1(-7.6;3.7),0.46 |
| 8 | rs2954029 | [*AC091114.1*](https://www.ebi.ac.uk/gwas/genes/AC091114.1) | 126490972 | A | T | 0.51 | -0.4(-6.1;5.6),0.9 | -0.1(-5.4;5.5),0.97 |
| 8 | rs11136341 | *PLEC1* | 145043543 | G | A | 0.36 | 0.2(-5.9;6.7),0.95 | 1.4(-4.3;7.4),0.64 |
| 9 | rs3780181 | *VLDLR* | 2640759 | A | G | 0.93 | 4(-7.4;16.8),0.51 | 3(-7.5;14.7),0.59 |
| 9 | rs9411489 | *ABO* | 136155000 | T | C | 0.21 | -0.6(-7.5;6.8),0.87 | -2(-8.3;4.7),0.55 |
| 10 | rs1129555 | *GPAM* | 113910721 | A | G | 0.28 | -4.6(-10.6;1.9),0.16 | -3.2(-8.9;2.8),0.29 |
| 11 | rs174546 | *FADS1-2-3* | 61569830 | C | T | 0.68 | -3.6(-9.5;2.7),0.26 | -2.6(-8.1;3.3),0.38 |
| 11 | rs964184 | *APOA1-C3-A4-A5* | 116648917 | G | C | 0.14 | -1.8(-9.7;6.8),0.67 | -2.4(-9.7;5.5),0.54 |
| 11 | rs11220462 | *ST3GAL4* | 126243952 | A | G | 0.14 | -3(-11;5.7),0.49 | -3.6(-10.9;4.4),0.37 |
| 12 | rs11065987 | *BRAP* | 112072424 | A | G | 0.54 | 1.9(-4.1;8.2),0.55 | 1.7(-3.8;7.4),0.56 |
| 12 | rs1169288 | *HNF1A* | 121416650 | C | A | 0.34 | 3.1(-3.2;9.7),0.34 | 2(-3.7;8),0.5 |
| 13 | rs4942486 | *BRCA2* | 32953388 | T | C | 0.47 | -4.6(-10.1;1.2),0.12 | -3.8(-8.9;1.5),0.16 |
| 14 | rs8017377 | *NYNRIN* | 24883887 | A | G | 0.46 | 2.2(-3.7;8.3),0.47 | 1.6(-3.8;7.2),0.57 |
| 16 | rs3764261 | *CETP* | 56993324 | C | A | 0.69 | -2.4(-9.1;3.9),0.47 | -2.2(-8.4;3.6),0.47 |
| 16 | rs2000999 | *HPR* | 72108093 | A | G | 0.2 | 0(-7.1;7.5),0.99 | 0.5(-6;7.5),0.88 |
| 17 | rs314253 | *DLG4* | 7091650 | T | C | 0.64 | 3.8(-2.4;10.4),0.24 | 4.4(-1.3;10.5),0.14 |
| 17 | rs7225700 | *OSBPL7* | 45391804 | C | T | 0.64 | -1.2(-7.1;5.1),0.71 | -0.7(-6.1;5.1),0.82 |
| 17 | rs1801689 | *APOH* | 64210580 | C | A | 0.03 | 0.3(-15.6;19.2),0.97 | -0.1(-14.8;17.1),0.99 |
| 19 | rs6511720 | *LDLR* | 11202306 | G | T | 0.89 | 12.5(2.6;23.5),0.01 | 10.3(1.3;20.1),0.02 |
| 19 | rs10401969 | *CILP2* | 19407718 | T | C | 0.92 | 2.8(-8.1;15),0.63 | -0.7(-10.5;10.2),0.9 |
| 19 | rs4420638 | *APOE* | 45422946 | G | A | 0.17 | 4.9(-3.5;14),0.26 | 4.1(-3.6;12.4),0.3 |
| 20 | rs364585 | *SPTLC3* | 12962718 | G | A | 0.62 | -4.9(-10.5;1.1),0.11 | -4.5(-9.7;1),0.11 |
| 20 | rs2328223 | *SNX5* | 17845921 | C | A | 0.18 | 2.1(-5.4;10.1),0.59 | 3.6(-3.4;11.1),0.32 |
| 20 | rs2902941 | *MAFB* | 39091514 | A | G | 0.68 | -2.9(-8.8;3.4),0.36 | -3.4(-8.9;2.4),0.24 |
| 20 | rs6029526 | *TOP1* | 39672618 | A | T | 0.49 | -1.6(-7.3;4.4),0.59 | -1.7(-6.9;3.9),0.55 |
| 22 | rs5763662 | *MTMR3* | 30378703 | T | C | 0.02 | -0.1(-18;21.7),0.99 | -1.9(-18.2;17.7),0.84 |
| 22 | rs4253772 | *PPARA* | 46627603 | C | T | 0.89 | 4.3(-5.1;14.6),0.39 | 3.3(-5.3;12.7),0.47 |

**Table S2(i).** Association of high-density lipoprotein-cholesterol-associated SNPs with log(obs)–log(exp) and the 5-year progression of CAC in the Heinz Nixdorf Recall study

| CHR | SNP | Gene(s) in/nearby region | BP | CA | NCA | CAF | log(obs)–log(exp)  %(95%CI),p | 5-year progression of CAC  %(95%CI),p |
| --- | --- | --- | --- | --- | --- | --- | --- | --- |
| 1 | rs12748152 | *PIGV-NROB2* | 27138393 | C | T | 0.92 | -3.2(-13.2;7.9),0.56 | -2.8(-12.1;7.5),0.58 |
| 1 | rs4660293 | *PABPC4* | 40028180 | A | G | 0.75 | -0.5(-7.1;6.5),0.88 | 0.4(-5.7;6.9),0.9 |
| 1 | rs12145743 | *HDGF-PMVK* | 156700651 | G | T | 0.37 | 3.2(-3;9.7),0.32 | 2.2(-3.4;8.2),0.45 |
| 1 | rs4650994 | *ANGPTL1* | 178515312 | G | A | 0.48 | -5.4(-10.8;0.3),0.06 | -4.6(-9.6;0.8),0.09 |
| 1 | rs1689800 | *ZNF648* | 182168885 | A | G | 0.62 | 1.5(-4.6;7.9),0.64 | 0.7(-4.9;6.6),0.81 |
| 1 | rs4846914 | *GALNT2* | 230295691 | A | G | 0.61 | 2.3(-3.7;8.7),0.46 | 1.9(-3.6;7.7),0.51 |
| 2 | rs1042034 | *APOB* | 21225281 | C | T | 0.21 | -1.3(-8.3;6.1),0.72 | -0.8(-7.2;6.1),0.82 |
| 2 | rs12328675 | *COBLL1* | 165540800 | C | T | 0.14 | 4.8(-3.8;14.2),0.28 | 2.9(-4.9;11.4),0.47 |
| 2 | rs7422339 | *CPS1* | 211540507 | C | A | 0.68 | -0.4(-6.6;6.1),0.89 | -0.1(-5.8;6),0.98 |
| 2 | rs1515100 | *IRS1* | 227128917 | C | A | 0.37 | -1.8(-7.6;4.3),0.56 | -0.6(-6;5.1),0.84 |
| 3 | rs2606736 | *ATG7* | 11400249 | C | T | 0.38 | -3.5(-9.2;2.5),0.24 | -2.2(-7.5;3.4),0.43 |
| 3 | rs2290547 | *SETD2* | 47061183 | G | A | 0.83 | -3.9(-11.1;3.9),0.32 | -2.3(-9.1;5),0.53 |
| 3 | rs2013208 | *RBM5* | 50129399 | T | C | 0.5 | -1.8(-7.5;4.3),0.56 | -1.5(-6.8;4.1),0.59 |
| 3 | rs13326165 | *STAB1* | 52532118 | A | G | 0.2 | -4.6(-11.4;2.7),0.21 | -3.3(-9.7;3.5),0.33 |
| 3 | rs6805251 | *GSK3B* | 119560606 | T | C | 0.4 | 6.2(0;12.8),0.05 | 6.1(0.4;12.1),0.04 |
| 3 | rs17404153 | *ACAD11* | 132163200 | T | G | 0.12 | -1.1(-9.8;8.3),0.81 | -2.4(-10.2;6.2),0.58 |
| 4 | rs10019888 | *C4orf52* | 26062990 | A | G | 0.84 | 4.6(-3.4;13.3),0.27 | 3.7(-3.6;11.6),0.33 |
| 4 | rs3822072 | *FAM13A* | 89741269 | G | A | 0.52 | 0.2(-5.6;6.3),0.95 | 0.4(-5;6),0.9 |
| 4 | rs2602836 | *ADH5* | 100014805 | A | G | 0.43 | 6.9(0.8;13.5),0.03 | 5.5(-0.1;11.4),0.06 |
| 4 | rs13107325 | *SLC39A8* | 103188709 | C | T | 0.94 | 5.2(-7;19.1),0.42 | 6.8(-4.7;19.8),0.26 |
| 5 | rs6450176 | *ARL15* | 53298025 | G | A | 0.76 | 0.3(-6.4;7.4),0.94 | 1.1(-5;7.7),0.72 |
| 6 | rs2814944 | *C6orf106* | 34552797 | G | A | 0.84 | 9.1(0.7;18.2),0.03 | 7.2(-0.4;15.4),0.06 |
| 6 | rs998584 | *VEGFA* | 43757896 | C | A | 0.53 | -3.3(-8.9;2.5),0.26 | -1.8(-7;3.7),0.52 |
| 6 | rs1936800 | *RSPO3* | 127436064 | C | T | 0.48 | -1.9(-7.6;4.1),0.53 | -2(-7.2;3.5),0.47 |
| 6 | rs605066 | *CITED2* | 139829666 | T | C | 0.58 | -3.2(-9;2.9),0.3 | -4(-9.3;1.6),0.16 |
| 6 | rs1084651 | *LPA* | 161089817 | G | A | 0.84 | -3.5(-11;4.6),0.39 | -3(-10;4.5),0.42 |
| 7 | rs702485 | *DAGLB* | 6449272 | G | A | 0.43 | 2.4(-3.5;8.7),0.43 | 2.6(-2.9;8.3),0.36 |
| 7 | rs4142995 | *SNX13* | 17919258 | G | T | 0.58 | -3.4(-9;2.5),0.25 | -3.3(-8.4;2.2),0.24 |
| 7 | rs4917014 | *IKZF1* | 50305863 | G | T | 0.33 | 1.7(-4.5;8.3),0.6 | 0.1(-5.5;6.1),0.97 |
| 7 | rs17145738 | *MLXIPL* | 72982874 | T | C | 0.11 | -1.4(-10.2;8.2),0.76 | 0.6(-7.6;9.6),0.89 |
| 7 | rs4731702 | *KLF14* | 130433384 | T | C | 0.49 | 2(-3.8;8.2),0.51 | 0.2(-5.1;5.8),0.95 |
| 7 | rs17173637 | *TMEM176A* | 150529449 | T | C | 0.89 | -0.4(-9.5;9.5),0.93 | -1.6(-9.9;7.4),0.72 |
| 8 | rs9987289 | *PPP1R3B* | 9183358 | G | A | 0.91 | 4(-6.6;15.7),0.47 | 4.6(-5.2;15.4),0.37 |
| 8 | rs12678919 | *LPL* | 19844222 | G | A | 0.09 | 6.1(-4.5;17.8),0.27 | 4.9(-4.7;15.6),0.33 |
| 8 | rs2293889 | *TRPS1* | 116599199 | G | T | 0.57 | 1.7(-4.2;8),0.58 | 0(-5.4;5.6),0.99 |
| 8 | rs2954029 | [*AC091114.1*](https://www.ebi.ac.uk/gwas/genes/AC091114.1) | 126490972 | T | A | 0.49 | 0.4(-5.3;6.5),0.9 | 0.1(-5.2;5.7),0.97 |
| 9 | rs643531 | *TTC39B* | 15296034 | A | C | 0.87 | -1.2(-9.3;7.7),0.79 | -1.3(-8.9;6.8),0.74 |
| 9 | rs1883025 | *ABCA1* | 107664301 | C | T | 0.76 | 4.8(-2.2;12.4),0.18 | 4.4(-2.1;11.3),0.19 |
| 10 | rs970548 | *MARCH8-ALLOX5* | 46013277 | C | A | 0.25 | -2.7(-9.2;4.2),0.44 | -2.5(-8.5;3.9),0.44 |
| 11 | rs2923084 | *AMPD3* | 10388782 | A | G | 0.82 | 2(-5.5;10.3),0.61 | 3(-4.1;10.6),0.42 |
| 11 | rs3136441 | *LRP4* | 46743247 | C | T | 0.14 | -0.3(-8.3;8.5),0.95 | -1(-8.4;6.9),0.79 |
| 11 | rs11246602 | *OR4C46* | 51512090 | C | T | 0.13 | 1.2(-7.4;10.6),0.79 | 0.7(-7.2;9.4),0.86 |
| 11 | rs174546 | *FADS1-2-3* | 61569830 | C | T | 0.68 | -3.6(-9.5;2.7),0.26 | -2.6(-8.1;3.3),0.38 |
| 11 | rs12801636 | *KAT5* | 65391317 | A | G | 0.22 | -0.9(-7.7;6.5),0.81 | -2(-8.2;4.6),0.54 |
| 11 | rs499974 | *MOGAT2-DGAT2* | 75455021 | C | A | 0.81 | -5.8(-12.8;1.6),0.12 | -4.2(-10.7;2.8),0.23 |
| 11 | rs964184 | *APOA1-C3-A4-A5* | 116648917 | C | G | 0.86 | 1.8(-6.4;10.8),0.67 | 2.5(-5.2;10.8),0.54 |
| 11 | rs7941030 | *UBASH3B* | 122522375 | C | T | 0.41 | 0.6(-5.3;6.9),0.85 | 0.5(-5;6.3),0.87 |
| 12 | rs7134375 | *PDE3A* | 20473758 | A | C | 0.42 | -6.5(-12;-0.7),0.03 | -5.1(-10.3;0.3),0.06 |
| 12 | rs3741414 | *LRP1* | 57844049 | T | C | 0.25 | 1.3(-5.4;8.6),0.71 | 1.8(-4.5;8.5),0.59 |
| 12 | rs7134594 | *MVK* | 110000193 | T | C | 0.54 | 2.4(-3.6;8.7),0.44 | 1.7(-3.8;7.5),0.56 |
| 12 | rs4759375 | *SBNO1* | 123796238 | T | C | 0.08 | 7.6(-3.4;19.9),0.18 | 6.9(-3.3;18),0.19 |
| 12 | rs4765127 | *ZNF664* | 124460167 | T | G | 0.32 | -2.1(-8;4.2),0.5 | -2.8(-8.2;3),0.34 |
| 12 | rs838880 | *SCARB1* | 125261593 | C | T | 0.32 | -2.2(-8.2;4.3),0.5 | -2.1(-7.6;3.8),0.48 |
| 14 | rs4983559 | *ZBTB42-AKT1* | 105277209 | G | A | 0.41 | 6.4(0.2;13),0.04 | 4.8(-0.8;10.8),0.1 |
| 15 | rs1532085 | *LIPC* | 58683366 | A | G | 0.36 | 1.2(-4.9;7.6),0.71 | 0.2(-5.3;6.1),0.94 |
| 15 | rs2652834 | *LACTB* | 63396867 | G | A | 0.8 | -2.2(-9.1;5.2),0.55 | -2.1(-8.5;4.7),0.54 |
| 16 | rs1121980 | *FTO* | 53809247 | G | A | 0.56 | -1.9(-7.6;4.1),0.52 | -1.8(-7.1;3.7),0.51 |
| 16 | rs3764261 | *CETP* | 56993324 | A | C | 0.31 | 2.4(-3.9;9.1),0.47 | 2.2(-3.6;8.4),0.47 |
| 16 | rs16942887 | *LCAT* | 67928042 | A | G | 0.14 | -5.3(-13.1;3.2),0.21 | -4.9(-12.1;2.9),0.21 |
| 16 | rs2925979 | *CMIP* | 81534790 | C | T | 0.7 | -6.2(-12.1;0),0.05 | -6.9(-12.3;-1.2),0.02 |
| 17 | rs11869286 | *STARD3* | 37813856 | C | G | 0.66 | 1.5(-4.9;7.5),0.64 | 2.1(-3.8;7.6),0.47 |
| 17 | rs4148008 | *ABCA8* | 66875294 | C | G | 0.71 | 3.4(-3.1;10.4),0.31 | 1.9(-4;8.2),0.54 |
| 17 | rs4129767 | *PGS1* | 76403984 | A | G | 0.51 | 1.6(-4.3;7.8),0.6 | -2.6(-7.8;2.9),0.35 |
| 18 | rs7241918 | *LIPG* | 47160953 | T | G | 0.84 | -2.3(-9.8;5.9),0.57 | -2.4(-9.3;5.1),0.52 |
| 18 | rs12967135 | *MC4R* | 57849023 | G | A | 0.77 | -2.4(-8.9;4.7),0.5 | -1.8(-7.9;4.8),0.59 |
| 19 | rs7255436 | *ANGPTL4* | 8433196 | A | C | 0.53 | 3.3(-2.7;9.7),0.29 | 3.1(-2.4;9),0.28 |
| 19 | rs737337 | *ANGPTL8* | 11347493 | T | C | 0.91 | 5.4(-5.3;17.2),0.34 | 4.2(-5.5;15),0.41 |
| 19 | rs731839 | *PEPD* | 33899065 | A | G | 0.65 | -5.2(-11;0.9),0.09 | -3(-8.5;2.8),0.31 |
| 19 | rs4420638 | *APOE* | 45422946 | A | G | 0.83 | -4.7(-12.3;3.6),0.26 | -4(-11;3.7),0.3 |
| 19 | rs17695224 | *HAS1* | 52324216 | G | A | 0.73 | -2.8(-9;3.7),0.39 | -2.4(-8.1;3.7),0.43 |
| 19 | rs386000 | *LILRA3* | 54792761 | C | G | 0.2 | 0(-7.3;7.8),1 | 1(-5.8;8.2),0.79 |
| 20 | rs1800961 | *HNF4A* | 43042364 | C | T | 0.98 | 24.1(2.5;50.4),0.03 | 25.3(5;49.4),0.01 |
| 20 | rs6065906 | *PLTP* | 44554015 | T | C | 0.82 | -7.9(-14.7;-0.6),0.03 | -6.6(-13;0.2),0.06 |
| 22 | rs181362 | *UBE2L3* | 21932068 | C | T | 0.8 | -8(-14.5;-0.9),0.03 | -6.8(-13;-0.3),0.04 |

**Table S2(j).** Association of triglyceride-associated SNPs with log(obs)–log(exp) and the 5-year progression of CAC in the Heinz Nixdorf Recall study

| CHR | SNP | Gene(s) in/nearby region | BP | CA | NCA | CAF | log(obs)–log(exp)  %(95%CI),p | 5-year progression of CAC  %(95%CI),p |
| --- | --- | --- | --- | --- | --- | --- | --- | --- |
| 1 | rs12748152 | *PIGV-NROB2* | 27138393 | T | C | 0.08 | 3.3(-7.4;15.3),0.56 | 2.9(-7;13.8),0.58 |
| 1 | rs2131925 | *ANGPTL3* | 63025942 | T | G | 0.68 | 2.5(-3.9;9.2),0.46 | 2.3(-3.6;8.5),0.45 |
| 1 | rs1321257 | *GALNT2* | 230305312 | G | A | 0.38 | -2.7(-8.5;3.4),0.37 | -2.1(-7.4;3.5),0.46 |
| 2 | rs1042034 | *APOB* | 21225281 | T | C | 0.79 | 1.3(-5.8;9),0.72 | 0.8(-5.8;7.8),0.82 |
| 2 | rs1260326 | *GCKR* | 27730940 | T | C | 0.42 | -2.2(-7.9;3.9),0.47 | -1.9(-7.1;3.7),0.5 |
| 2 | rs10195252 | *COBLL1* | 165513091 | T | C | 0.57 | -5.7(-11.2;0.1),0.06 | -3.7(-8.9;1.8),0.18 |
| 2 | rs2943645 | *IRS1* | 227099180 | T | C | 0.63 | 2.7(-3.3;9.2),0.38 | 1.3(-4.2;7.2),0.64 |
| 3 | rs645040 | *MSL2L1* | 135926622 | T | G | 0.8 | 3.3(-4;11.2),0.39 | 2.3(-4.4;9.5),0.51 |
| 4 | rs6831256 | *LRPAP1* | 3473139 | G | A | 0.42 | 2.5(-3.4;8.7),0.41 | 2.3(-3.1;8),0.41 |
| 4 | rs442177 | *KLHL8* | 88030261 | T | G | 0.59 | 1.7(-4.3;8.1),0.59 | 1.6(-3.9;7.4),0.58 |
| 5 | rs9686661 | *MAP3K1* | 55861786 | T | C | 0.18 | -1.6(-8.8;6.2),0.68 | -2.9(-9.5;4.1),0.4 |
| 5 | rs1553318 | *TIMD4* | 156479323 | G | C | 0.37 | -1.6(-7.4;4.5),0.6 | 1.5(-4.2;6.8),0.6 |
| 6 | rs2247056 | *HLA* | 31265490 | C | T | 0.74 | -1.2(-7.6;5.6),0.71 | -0.1(-6.1;6.2),0.96 |
| 6 | rs998584 | *VEGFA* | 43757896 | A | C | 0.47 | 3.4(-2.5;9.7),0.26 | 1.8(-3.6;7.5),0.52 |
| 6 | rs1936800 | *RSPO3* | 127436064 | T | C | 0.52 | 1.9(-4;8.2),0.53 | 2(-3.4;7.8),0.47 |
| 7 | rs4722551 | *MIR148A* | 25991826 | C | T | 0.16 | 3.7(-4.4;12.5),0.38 | 3.7(-3.8;11.8),0.34 |
| 7 | rs13238203 | *TYW1B* | 72129667 | C | T | 0.96 | 2.5(-8.2;14.6),0.66 | 0.1(-9.6;10.9),0.98 |
| 7 | rs7811265 | *MLXIPL* | 72934510 | A | G | 0.82 | -1.7(-9;6.2),0.66 | -2.9(-9.6;4.2),0.41 |
| 7 | rs38855 | *MET* | 116358044 | A | G | 0.55 | 1.8(-4.2;8),0.57 | 2.5(-3;8.4),0.37 |
| 8 | rs11776767 | *PINX1* | 10683929 | C | G | 0.35 | -3.5(-9.3;2.6),0.26 | -3.8(-9.1;1.8),0.18 |
| 8 | rs1495741 | *NAT2* | 18272881 | G | A | 0.23 | -5.4(-11.9;1.5),0.12 | -4(-10;2.4),0.21 |
| 8 | rs12678919 | *LPL* | 19844222 | A | G | 0.91 | -5.7(-15.1;4.7),0.27 | -4.7(-13.5;4.9),0.33 |
| 8 | rs2954029 | [*AC091114.1*](https://www.ebi.ac.uk/gwas/genes/AC091114.1) | 126490972 | A | T | 0.51 | -0.4(-6.1;5.6),0.9 | -0.1(-5.4;5.5),0.97 |
| 10 | rs1832007 | *AKR1C4* | 5254847 | A | G | 0.85 | -5.5(-13;2.7),0.19 | -3.6(-10.7;4.1),0.36 |
| 10 | rs10761731 | *JMJD1C* | 65027610 | A | T | 0.56 | -1.7(-7.6;4.6),0.59 | -0.2(-5.7;5.7),0.96 |
| 10 | rs2068888 | *CYP26A1* | 94839642 | G | A | 0.54 | 5.1(-1;11.5),0.1 | 3.6(-2;9.4),0.21 |
| 11 | rs174546 | *FADS1-2-3* | 61569830 | T | C | 0.32 | 3.7(-2.7;10.5),0.26 | 2.6(-3.2;8.8),0.38 |
| 11 | rs964184 | *APOA1-C3-A4-A5* | 116648917 | G | C | 0.14 | -1.8(-9.7;6.8),0.67 | -2.4(-9.7;5.5),0.54 |
| 12 | rs11613352 | *LRP1* | 57792580 | C | T | 0.75 | -0.6(-7.2;6.5),0.87 | -1.1(-7.2;5.4),0.74 |
| 12 | rs4765127 | *ZNF664* | 124460167 | G | T | 0.68 | -2.1(-8;4.2),0.5 | 2.8(-2.9;8.9),0.34 |
| 15 | rs2412710 | *CAPN3* | 42683787 | A | G | 0.02 | 7.4(-13.1;32.6),0.51 | 3.9(-14.5;26.2),0.7 |
| 15 | rs2929282 | *FRMD5* | 44245931 | T | A | 0.04 | -5.9(-18.7;9.1),0.42 | -6.8(-18.6;6.8),0.31 |
| 15 | rs261342 | *LIPC* | 58731153 | G | C | 0.2 | 1(-6.4;8.9),0.8 | -1.1(-7.7;6),0.76 |
| 16 | rs3198697 | *PDXDC1* | 15129940 | C | T | 0.61 | 2.1(-3.8;8.4),0.49 | 1.6(-3.8;7.4),0.57 |
| 16 | rs11649653 | *CTF1* | 30918487 | C | G | 0.6 | 1.3(-4.7;7.7),0.68 | 1.6(-4;7.5),0.58 |
| 16 | rs1121980 | *FTO* | 53809247 | G | A | 0.56 | -1.9(-7.6;4.1),0.52 | -1.8(-7.1;3.7),0.51 |
| 16 | rs3764261 | *CETP* | 56993324 | C | A | 0.69 | -2.4(-9.1;3.9),0.47 | -2.2(-8.4;3.6),0.47 |
| 16 | rs7205804 | *CETP* | 57004889 | G | A | 0.57 | -2.1(-7.8;3.9),0.48 | -2.5(-7.8;3),0.36 |
| 17 | rs8077889 | *MPP3* | 41878166 | C | A | 0.2 | 3.6(-3.8;11.7),0.35 | 5.4(-1.6;13),0.13 |
| 19 | rs7248104 | *INSR* | 7224431 | G | A | 0.6 | 0(-5.8;6.2),1 | 0.9(-4.6;6.6),0.76 |
| 19 | rs10401969 | *CILP2* | 19407718 | T | C | 0.92 | 2.8(-8.1;15),0.63 | -0.7(-10.5;10.2),0.9 |
| 19 | rs731839 | *PEPD* | 33899065 | G | A | 0.35 | 5.5(-0.9;12.4),0.09 | 3.1(-2.7;9.3),0.31 |
| 19 | rs439401 | *APOE-C1-C2* | 45414451 | C | T | 0.65 | 4.2(-2.1;10.9),0.19 | 4.7(-1.2;10.8),0.12 |
| 20 | rs4810479 | *PLTP* | 44545048 | C | T | 0.25 | 6.7(-0.3;14.1),0.06 | 5.3(-1.1;12.1),0.1 |
| 22 | rs5756931 | *PLA2G6* | 38546033 | T | C | 0.61 | -3(-8.8;3.2),0.33 | -3.7(-9.1;1.9),0.19 |

**Table S2(k)**. Association of total cholesterol-associated SNPs with log(obs)–log(exp) and the 5-year progression of CAC in the Heinz Nixdorf Recall study

| CHR | SNP | Gene(s) in/nearby region | BP | CA | NCA | CAF | log(obs)–log(exp)  %(95%CI),p | 5-year progression of CAC  %(95%CI),p |
| --- | --- | --- | --- | --- | --- | --- | --- | --- |
| 1 | rs1077514 | *ASAP3* | 23766233 | T | C | 0.86 | 3.6(-4.8;12.6),0.41 | 3.1(-4.6;11.4),0.44 |
| 1 | rs12027135 | *LDLRAP1* | 25775733 | T | A | 0.57 | -2.2(-7.8;3.8),0.47 | -0.3(-5.6;5.3),0.91 |
| 1 | rs2479409 | *PCSK9* | 55504650 | G | A | 0.35 | 3.1(-3.1;9.8),0.34 | 1.1(-4.6;7.1),0.71 |
| 1 | rs2131925 | *ANGPTL3* | 63025942 | T | G | 0.68 | 2.5(-3.9;9.2),0.46 | 2.3(-3.6;8.5),0.45 |
| 1 | rs7515577 | *EVI5* | 93009438 | A | C | 0.8 | 8.6(0.8;17),0.03 | 8(0.9;15.7),0.03 |
| 1 | rs629301 | *SORT1* | 109818306 | T | G | 0.77 | 0.7(-6.2;8),0.85 | 1.2(-5.1;8),0.71 |
| 1 | rs2642442 | *MOSC1* | 220973563 | T | C | 0.69 | 8.5(1.7;15.7),0.01 | 9.2(2.8;15.9),0.01 |
| 1 | rs514230 | *IRF2BP2* | 234858597 | T | A | 0.52 | 3.5(-2.5;9.9),0.26 | 3.3(-2.2;9.1),0.25 |
| 2 | rs1367117 | *APOB* | 21263900 | A | G | 0.32 | 1.6(-4.7;8.4),0.62 | 0.9(-4.9;7.1),0.76 |
| 2 | rs1260326 | *GCKR* | 27730940 | T | C | 0.42 | -2.2(-7.9;3.9),0.47 | -1.9(-7.1;3.7),0.5 |
| 2 | rs4299376 | *ABCG5/8* | 44072576 | G | T | 0.31 | -1.4(-7.5;5.2),0.67 | -1.9(-7.6;4.1),0.52 |
| 2 | rs10490626 | *INSIG2* | 118835841 | A | G | 0.07 | 7.4(-4.5;20.7),0.23 | 4.8(-5.9;16.7),0.39 |
| 2 | rs2030746 | *LOC84931* | 121309488 | T | C | 0.41 | 2.1(-3.9;8.5),0.51 | 0.3(-5.1;6.1),0.9 |
| 2 | rs7570971 | *RAB3GAP1* | 135837906 | A | C | 0.43 | 3.7(-2.3;10.1),0.23 | 2.6(-2.9;8.3),0.37 |
| 2 | rs2287623 | *ABCB11* | 169830155 | G | A | 0.39 | 3.4(-2.7;9.9),0.28 | 5.2(-0.6;11.2),0.08 |
| 2 | rs11694172 | *FAM117B* | 203532304 | G | A | 0.24 | -4.7(-11;2),0.17 | -5.7(-11.5;0.4),0.07 |
| 2 | rs11563251 | *UGT1A1* | 234679384 | T | C | 0.09 | -4(-13.6;6.7),0.45 | -1.2(-10.3;8.8),0.8 |
| 3 | rs2290159 | *RAF1* | 12628920 | G | C | 0.81 | -0.3(-7.5;7.5),0.93 | -0.6(-7.2;6.6),0.87 |
| 3 | rs7640978 | *CMTM6* | 32533010 | C | T | 0.92 | 2.3(-8.6;14.4),0.69 | 1.1(-8.8;12.1),0.83 |
| 3 | rs13315871 | *PXK* | 58381287 | G | A | 0.91 | 1.9(-8;12.9),0.71 | 2.9(-6.4;13.1),0.55 |
| 4 | rs6831256 | *LRPAP1* | 3473139 | A | G | 0.58 | -1.7(-7.4;4.2),0.56 | -1.7(-6.9;3.8),0.54 |
| 5 | rs12916 | *HMGCR* | 74656539 | C | T | 0.42 | 3.3(-2.8;9.8),0.3 | 4(-1.6;10),0.17 |
| 5 | rs4530754 | *CSNK1G3* | 122855416 | A | G | 0.57 | 5.1(-1;11.6),0.11 | 4.2(-1.4;10.1),0.15 |
| 5 | rs6882076 | *TIMD4* | 156390297 | C | T | 0.62 | 1.5(-4.5;7.8),0.63 | 1.9(-3.6;7.7),0.5 |
| 6 | rs3757354 | *MYLIP* | 16127407 | C | T | 0.78 | 6.7(-0.6;14.6),0.07 | 5.8(-0.9;13),0.09 |
| 6 | rs1800562 | *HFE* | 26093141 | G | A | 0.96 | 5.4(-8.6;21.6),0.47 | 5.3(-7.7;20),0.44 |
| 6 | rs3177928 | *HLA* | 32412435 | A | G | 0.15 | 0.2(-7.9;9),0.97 | 1(-6.5;9.2),0.79 |
| 6 | rs2814982 | *C6orf106* | 34546560 | C | T | 0.89 | 5.4(-3.9;15.6),0.27 | 4.2(-4.3;13.5),0.34 |
| 6 | rs2758886 | *KCNK17* | 39250837 | A | G | 0.28 | -0.9(-7.1;5.7),0.78 | -1.8(-7.5;4.3),0.56 |
| 6 | rs9488822 | *FRK* | 116312893 | T | A | 0.31 | -7.5(14.7;0.8),0.03 | -5.5(-12;0.6),0.08 |
| 6 | rs9376090 | *HBS1L* | 135411228 | C | T | 0.27 | 0.7(-5.8;7.5),0.85 | 1.2(-4.8;7.6),0.7 |
| 6 | rs1564348 | *LPA* | 160578860 | C | T | 0.17 | -0.1(-7.8;8.2),0.98 | 0.3(-7.3;7.4),0.94 |
| 7 | rs1997243 | *GPR146* | 1083777 | G | A | 0.18 | 1.2(-6.3;9.2),0.77 | 1.3(-5.6;8.7),0.72 |
| 7 | rs12670798 | *DNAH11* | 21607352 | C | T | 0.24 | 3(-4;10.5),0.42 | 1.9(-4.5;8.7),0.57 |
| 7 | rs4722551 | *MIR148A* | 25991826 | C | T | 0.16 | 3.8(-4.5;12.9),0.37 | 4(-3.7;12.3),0.32 |
| 7 | rs2072183 | *NPC1L1* | 44579180 | C | G | 0.24 | 4(-3;11.6),0.27 | 3.6(-2.9;10.5),0.29 |
| 8 | rs2126259 | *PPP1R3B* | 9185146 | C | T | 0.9 | 2.3(-7.5;13.3),0.66 | 1.8(-7.3;11.8),0.7 |
| 8 | rs1495741 | *NAT2* | 18272881 | G | A | 0.23 | -5.4(-11.9;1.5),0.12 | -4(-10;2.4),0.21 |
| 8 | rs10102164 | *SOX17* | 55421614 | A | G | 0.19 | -3.8(-10.8;3.8),0.32 | -4.5(-10.9;2.3),0.19 |
| 8 | rs2081687 | *CYP7A1* | 59388565 | T | C | 0.36 | -1(-6.9;5.3),0.76 | -1.8(-7.2;4),0.53 |
| 8 | rs2737229 | *TRPS1* | 116648565 | A | C | 0.71 | -0.9(-7.2;5.7),0.77 | -0.5(-6.3;5.6),0.87 |
| 8 | rs2954029 | [*AC091114.1*](https://www.ebi.ac.uk/gwas/genes/AC091114.1) | 126490972 | A | T | 0.5 | 0.3(-5.5;6.4),0.93 | 0.5(-4.8;6.2),0.85 |
| 8 | rs11136341 | *PLEC1* | 145043543 | G | A | 0.36 | 0.2(-5.9;6.7),0.95 | 1.4(-4.3;7.4),0.64 |
| 9 | rs3780181 | *VLDLR* | 2640759 | A | G | 0.93 | 3.9(-7.5;16.7),0.52 | 2.9(-7.6;14.5),0.6 |
| 9 | rs581080 | *TTC39B* | 15305378 | C | G | 0.81 | -0.5(-7.7;7.3),0.91 | -0.8(-7.5;6.3),0.82 |
| 9 | rs1883025 | *ABCA1* | 107664301 | C | T | 0.76 | 4.8(-2.2;12.4),0.18 | 4.4(-2.1;11.3),0.19 |
| 9 | rs9411489 | *ABO* | 136155000 | T | C | 0.21 | -0.6(-7.5;6.8),0.87 | -2(-8.3;4.7),0.55 |
| 10 | rs10904908 | *VIM-CUBN* | 17260290 | G | A | 0.42 | -1.4(-7.1;4.7),0.65 | -1.7(-6.9;3.9),0.55 |
| 10 | rs970548 | *MARCH8-ALLOX5* | 46013277 | A | C | 0.75 | 2.2(-4.8;8.7),0.52 | 2.1(-4.4;8.1),0.52 |
| 10 | rs2255141 | *GPAM* | 113933886 | A | G | 0.28 | -4.3(-10.4;2.2),0.19 | -2.9(-8.6;3.1),0.33 |
| 11 | rs11603023 | *PHLDB1* | 118486067 | T | C | 0.41 | 1.8(-4.2;8.2),0.57 | 1.7(-3.8;7.6),0.55 |
| 11 | rs10128711 | *SPTY2D1* | 18632984 | C | T | 0.73 | -4.8(-11;1.8),0.15 | -4.7(-10.4;1.4),0.13 |
| 11 | rs174546 | *FADS1-2-3* | 61569830 | C | T | 0.68 | -3.6(-9.5;2.7),0.26 | -2.6(-8.1;3.3),0.38 |
| 11 | rs964184 | *APOA1-C3-A4-A5* | 116648917 | G | C | 0.14 | -1.8(-9.7;6.8),0.67 | -2.4(-9.7;5.5),0.54 |
| 11 | rs7941030 | *UBASH3B* | 122522375 | C | T | 0.41 | 0.6(-5.3;6.9),0.85 | 0.5(-5;6.3),0.87 |
| 11 | rs11220462 | *ST3GAL4* | 126243952 | A | G | 0.14 | -3.6(-11.6;5.1),0.4 | -4.2(-11.5;3.8),0.3 |
| 12 | rs4883201 | *PHC1-A2ML1* | 9082581 | A | G | 0.89 | 3.8(-5.5;14.1),0.43 | 2.8(-5.7;12.2),0.53 |
| 12 | rs11065987 | *BRAP* | 112072424 | A | G | 0.54 | 1.9(-4.1;8.2),0.55 | 1.7(-3.8;7.4),0.56 |
| 12 | rs1169288 | *HNF1A* | 121416650 | C | A | 0.34 | 3.1(-3.2;9.7),0.34 | 2(-3.7;8),0.5 |
| 15 | rs1532085 | *LIPC* | 58683366 | A | G | 0.36 | 1.2(-4.9;7.6),0.71 | 0.2(-5.3;6.1),0.94 |
| 16 | rs3764261 | *CETP* | 56993324 | A | C | 0.31 | 2.4(-3.9;9.1),0.47 | 2.2(-3.6;8.4),0.47 |
| 16 | rs2000999 | *HPR* | 72108093 | A | G | 0.2 | 0(-7.1;7.5),0.99 | 0.5(-6;7.5),0.88 |
| 17 | rs314253 | *DLG4* | 7091650 | T | C | 0.64 | 3.4(-2.8;10),0.29 | 4.1(-1.6;10.2),0.16 |
| 17 | rs7206971 | *EFCAB13* | 45425115 | A | G | 0.48 | 1.2(-4.6;7.3),0.7 | 1.9(-3.5;7.6),0.5 |
| 18 | rs7241918 | *LIPG* | 47160953 | T | G | 0.84 | -2.3(-9.8;5.9),0.57 | -2.4(-9.3;5.1),0.52 |
| 19 | rs6511720 | *LDLR* | 11202306 | G | T | 0.89 | 12.5(2.6;23.5),0.01 | 10.3(1.3;20.1),0.02 |
| 19 | rs10401969 | *CILP2* | 19407718 | T | C | 0.92 | 2.8(-8.1;15),0.63 | -0.7(-10.5;10.2),0.9 |
| 19 | rs4420638 | *APOE* | 45422946 | G | A | 0.17 | 4.9(-3.5;14),0.26 | 4.1(-3.6;12.4),0.3 |
| 19 | rs492602 | *FLJ36070* | 49206417 | G | A | 0.41 | 2.8(-3.1;9.1),0.36 | 2.4(-3.1;8.1),0.41 |
| 20 | rs2277862 | *ERGIC3* | 34152782 | C | T | 0.85 | -0.1(-8.1;8.5),0.98 | 1.5(-6;9.5),0.71 |
| 20 | rs2902940 | *MAFB* | 39091487 | A | G | 0.72 | -5.5(-11.5;1),0.09 | -5.5(-11;0.5),0.07 |
| 20 | rs6029526 | *TOP1* | 39672618 | A | T | 0.49 | -1.6(-7.3;4.4),0.59 | -1.7(-6.9;3.9),0.55 |
| 20 | rs1800961 | *HNF4A* | 43042364 | C | T | 0.98 | 24.1(2.5;50.4),0.03 | 25.3(5;49.4),0.01 |
| 22 | rs138777 | *TOM1* | 35711098 | A | G | 0.35 | -0.5(-6.5;5.8),0.86 | 0(-5.5;5.9),0.99 |
| 22 | rs4253772 | *PPARA* | 46627603 | T | C | 0.11 | -4.1(-12.7;5.4),0.39 | -3.1(-11.2;5.7),0.47 |

CHR: chromosome, BP: base position (hgBuild37), CA: coded allele, NCA: non coded allele, CAF: coded allele frequency, 95%CI: 95% confidence interval, CAC: coronary artery calcification, “log(obs)–log(exp)”: percent deviation from the expected (CAC_5y_+1). The association between each SNP and outcomes was carried out using linear regression in PLINK. The models are adjusted for age, sex and log(CAC_b_+1).
