# Supplementary material for: Genetic risk scores for coronary artery disease and its traditional risk factors: Their role in the progression of coronary artery calcification—Results of the Heinz Nixdorf Recall study
Source: PLoS One. 2020 May 7;15(5):e0232735. doi: 10.1371/journal.pone.0232735 (PMC7205301; doi:10.1371/journal.pone.0232735)
Supplement: S3 Table — CAD: coronary artery disease, CAC: coronary artery calcification. CHR: chromosome, BP: base position (hgBuild37), CA: coded allele, NCA: non coded allele, CAF: coded allele frequency, 95%CI: 95% confidence interval, CAC: coronary artery calcification, “log(obs)–log(exp)”: percent deviation from the expected (CAC5y+1). The association between each SNP and outcomes was carried out using linear regression in PLINK. The models are adjusted for age, sex and log(CACb+1). (DOCX) [file pone.0232735.s003.docx]

**Table S3.** Association of coronary artery disease and coronary artery calcification SNPs that showed an association with log(obs)–log(exp) or the 5-year progression in CAC or both at nominal significance level.

| Phenotype | CHR | SNP | BP | CA | NCA | CAF | log(obs)–log(exp)  %(95%CI),p | 5-year progression of CAC  %(95%CI),p |
| --- | --- | --- | --- | --- | --- | --- | --- | --- |
| CAD | 4 | rs7692387 | 156635309 | G | A | 0.8 | 9.7( 1.9;18.2),0.01 | 10.3(3;18.1),0.005 |
| CAD | 7 | rs11556924 | 129663496 | C | T | 0.61 | 7( 0.6;13.7),0.03 | 4.7(-1;10.7),0.11 |
| CAD | 9 | rs3217992 | 22003223 | T | C | 0.37 | 6.6( 0.3;13.3),0.04 | 4.7(-1;10.8),0.11 |
| CAD | 9 | rs1333049 | 22125503 | C | G | 0.45 | 7.1( 0.9;13.7),0.03 | 5.2(-0.5;11.2),0.07 |
| CAD | 10 | rs2047009 | 44539913 | G | T | 0.51 | 7.5( 1.3;14.1),0.02 | 4.9(-0.7;10.8),0.09 |
| CAD | 10 | rs501120 | 44753867 | T | C | 0.86 | 16.0( 6.3;26.4),0.001 | 13.5(4.8;22.9),0.002 |
| CAD | 10 | rs12413409 | 104719096 | G | A | 0.9 | 12.9( 2.4;24.5),0.01 | 11.2(1.6;21.6),0.02 |
| CAC | 9 | rs10965219 | 22053687 | G | A | 0.47 | 6.9(0.7;13.5),0.03 | 4.9(-0.7; 10.9),0.09 |
| CAC | 9 | rs1333049 | 22125503 | C | G | 0.45 | 7.1(0.9;13.7),0.03 | 5.2(-0.5; 11.2),0.07 |

CAD: coronary artery disease, CAC: coronary artery calcification. CHR: chromosome, BP: base position (hgBuild37), CA: coded allele, NCA: non coded allele, CAF: coded allele frequency, 95%CI: 95% confidence interval, CAC: coronary artery calcification, “log(obs)–log(exp)”: percent deviation from the expected (CAC_5y_+1). The association between each SNP and outcomes was carried out using linear regression in PLINK. The models are adjusted for age, sex and log(CAC_b_+1).
