# Supplementary material for: Genetic risk scores for coronary artery disease and its traditional risk factors: Their role in the progression of coronary artery calcification—Results of the Heinz Nixdorf Recall study
Source: PLoS One. 2020 May 7;15(5):e0232735. doi: 10.1371/journal.pone.0232735 (PMC7205301; doi:10.1371/journal.pone.0232735)
Supplement: S4 Table — CAD: coronary artery disease, CAC: coronary artery calcification, CADPlusCAC: CAD and CAC genetic risk scores are included as separate predictors in a linear regression model, EV: explained variance. The association between the genetic risk scores and outcomes was carried out using linear regression in SAS. The models are adjusted for age, sex and log(CACb+1). (DOCX) [file pone.0232735.s004.docx]

**Table S4.** Association between the coronary artery disease and coronary artery calcification genetic risk scores as separate predictors in a linear regression model with log(obs)–log(exp) and the 5-year progression in CAC.

|  | log(obs)–log(exp) | EV (%) | 5-year progression of CAC | EV (%) |
| --- | --- | --- | --- | --- |
|  | % deviation from expected (CAC+1) (95% CI), P |  | % change in (CAC+1) (95% CI), P |  |
| CADPlusCAC  Intercept  Age (years)  Sex  log(CAC_b_+1)  CAD GRS  CAC GRS | -63.3 (-74.2; -47.8), <0.0001  2.7 (2.1; 3.4), <0.0001  -17.1 (-24.3; -9.1), <0.0001  -8.9 (-10.8; -7.0), <0.0001  **8.5 (3.6; 13.8), 0.0006**  **2.4 (-2.4; 7.3), 0.33** | 0.6 | -42., (-58.2; -19.8), 0.001  2.6 (2.1; 3.2), <0.0001  -18.4(-25.0; -11.2), <0.0001  -5.3 (-7.1; -3.5), <0.0001  **6.4 (1.9; 11.1), 0.005**  **1.5 (-2.8; 6.0), 0.50** | 0.4 |

CAD: coronary artery disease, CAC: coronary artery calcification, CADPlusCAC: CAD and CAC genetic risk scores are included as separate predictors in a linear regression model, EV: explained variance. The association between the genetic risk scores and outcomes was carried out using linear regression in SAS. The models are adjusted for age, sex and log(CAC_b_+1).
