# Supplementary material for: Genetic risk scores for coronary artery disease and its traditional risk factors: Their role in the progression of coronary artery calcification—Results of the Heinz Nixdorf Recall study
Source: PLoS One. 2020 May 7;15(5):e0232735. doi: 10.1371/journal.pone.0232735 (PMC7205301; doi:10.1371/journal.pone.0232735)
Supplement: S5 Table — CAD: coronary artery disease, CAC: coronary artery calcification, CAD_CAC: combined CAD and CAC genetic risk score, BMI: body-mass index, TC: total cholesterol, EV: explained variance. The association between the genetic risk scores and outcomes was carried out using linear regression in SAS. The models are adjusted for age, sex, log(CACb+1) and family history of CHD. (DOCX) [file pone.0232735.s005.docx]

**Table S5**. Association between the genetic risk scores with log(obs)–log(exp) and the 5-year progression in CAC adjusted for a family history of CHD.

|  | log(obs)–log(exp) | EV (%) | 5-year progression of CAC | EV (%) |
| --- | --- | --- | --- | --- |
|  | % deviation from expected (CAC+1) (95% CI), P |  | % change in (CAC+1) (95% CI), P |  |
| Intercept  CAD GRS  Age (years)  Sex  log(CAC_b_+1)  Family history of CHD | -63.5 (-75.0; -46.7), <0.0001  **9.4 (4.7; 14.4), 6.2x10^-5^**  2.8 (2.2; 3.5), <0.0001  -18.9 (-26.3; -10.7), <0.0001  -9.7 (-11.6; -7.7), <0.0001  7.8 (-2.2; 18.8), 0.12 | **0.5** | -42.8 (-59.7;-18.9), 0.002  **6.7 (2.5; 11.2), 0.002**  2.7 (2.1; 3.3), <0.0001  -20.3 (-27.1; -13), <0.0001  -5.9(-7.8; -4.0), <0.0001  10.7 (1.2; 21.1), 0.03 | **0.3** |
| Intercept  CAC GRS  Age (years)  Sex  log(CAC_b_+1)  Family history of CHD | -62.9 (-74.6; -45.8), <0.0001  **6.9 (2.2; 11.8), 0.003**  2.8 (2.1; 3.4), <0.0001  -18.6 (-26.1; -10.4), <0.0001  -9.5 (-11.5; -7.6), <0.0001  7.8 (-2.2; 18.7), 0.13 | **0.3** | NA  NA  NA  NA  NA  NA | **NA** |
| Intercept  CAD_CAC GRS  Age (years)  Sex  log(CAC_b_+1)  Family history of CHD | -63.5 (-75.0; -46.7), <0.0001  **10.1 (5.3; 15.1), 0.0006**  2.8 (2.2; 3.5), <0.0001  -19.2 (-26.6; -11.1), <0.0001  -9.8 (-11.8; -7.8), <0.0001  7.6 (-2.4; 18.5), 0.14 | **0.4** | -42.8 (-59.6; -18.8), 0.002  **7.1 (2.8; 11.6), 0.00**  2.7 (2.1; 3.3), <0.0001  -20.6 (-27.3; -13.2), <0.0001  -6.0 (-7.9; -4.1), <0.0001  10.5 (1.1; 20.9), 0.03 | **0.4** |

CAD: coronary artery disease, CAC: coronary artery calcification, CAD_CAC: combined CAD and CAC genetic risk score, BMI: body-mass index, TC: total cholesterol, EV: explained variance. The association between the genetic risk scores and outcomes was carried out using linear regression in SAS. The models are adjusted for age, sex, log(CAC_b_+1) and family history of CHD.
