# Supplementary material for: Genetic risk scores for coronary artery disease and its traditional risk factors: Their role in the progression of coronary artery calcification—Results of the Heinz Nixdorf Recall study
Source: PLoS One. 2020 May 7;15(5):e0232735. doi: 10.1371/journal.pone.0232735 (PMC7205301; doi:10.1371/journal.pone.0232735)
Supplement: S6 Table — a) Association of the genetic risk score quartiles with the percentage deviation from the expected coronary artery calcification. GRS: genetic risk score, CAC: coronary artery calcification, CI: confidence interval, medium GRS quartile consists of Q2 and Q3 and high GRS quartile consist of Q4. The association between the genetic risk scores and outcome was carried out using linear regression in SAS. Model 1: adjusted for age, sex and log(CACb+1). Model 2: adjusted for age, sex, log(CACb+1) and coronary artery disease risk factors (type 2 diabetes, body mass index, socio economic status, systolic blood pressure, smoking, antihypertensive medication, lipid lowering medication, LDL, HDL). b) Association of the genetic risk score quartiles with the 5-year progression of coronary artery calcification. GRS: genetic risk score, CAD: coronary artery disease, CI: confidence interval, medium GRS quartile consists of Q2 and Q3 and high GRS quartile consist of Q4. The association between the genetic risk score and outcome was carried out using linear regression in SAS. Model 1: adjusted for age, sex and log(CACb+1). Model 2: adjusted for age, sex, log(CACb+1) and CAD risk factors (type 2 diabetes, body mass index, socio economic status, systolic blood pressure, smoking, antihypertensive medication, lipid lowering medication, LDL, HDL). (DOCX) [file pone.0232735.s006.docx]

**Table S6(a)**. Association of the genetic risk score quartiles with the percentage deviation from the expected coronary artery calcification

|  | N (%) | Percent deviation  from expected (CAC_5y_+1), (95% CI), P |
| --- | --- | --- |
| Model 1 |  |  |
| **CAD**  **Low GRS Quartile (Q1)**  **Medium GRS Quartile (Q2, Q3)**  **High GRS Quartile (Q4)**  Intercept  Age (years)  Sex  log(CACb+1) | 775 (25.0)  1548 (50.0)  774 (25.0) | **Reference**  **4.4 (-5.8; 15.6), 0.41**  **26.3 (12.2; 42.3), 0.0001**  -66.3 (-76.5; -51.5), <0.0001  2.7 (2.1; 3.3), <0.0001  -16.5 (-23.9; -8.6), 0.0001  -8.8 (-10.6; -6.9), <0.0001 |
| Model 2  **CAD**  **Low GRS Quartile (Q1)**  **Medium GRS Quartile (Q2, Q3)**  **High GRS Quartile (Q4)**  Intercept  Age (years)  Sex  log(CACb+1)  Diabetes  BMI  Systolic blood pressure  LDL-cholesterol  HDL-cholesterol  Social economic status  Antihypertensive medication  Lipid-lowering medication  Past smoker  Current smoker | 729 (25.3)  1440 (49.9)  716 (24.8) | **Reference**  **4.5 (-5.8; 16.0), 0.40**  **24.3 (10.1; 40.4), 0.0004**  -67.4 (-82.2; -40.4), 0.003  2.5 (1.8; 3.2), <0.0001  -13.1 (22.2; -2.9), 0.01  -10.5 (-12.4; -8.5), <0.0001  41.7 (23.1; 63.1), <0.0001  -3.2 (-4.3; -2.1), <0.0001  0.5 (0.3; 0.8), <0.0001  0.2 (0; 0.3), 0.008  -0.1(-0.4; 0.2), 0.5  -7.1 (-15.7; 2.4), 0.14  13.4 (2.6; 25.2), 0.01  17 (0.8; 35.9), 0.04  10.4 (-0.2; 22.1), 0.055  34.4 (19.5; 51.1),<0.0001 |
| Model 1  **CAC**  **Low GRS Quartile (Q1)**  **Medium GRS Quartile (Q2, Q3)**  **High GRS Quartile (Q4)**  Intercept  Age (years)  Sex  log(CACb+1) | 780 (25.2)  1652 (53.3)  665 (21.5) | **Reference**  **5.8 (-4.4; 17.1), 0.28**  **17.5 (3.9; 33.0), 0.01**  -65.3 (-75.8; -50.2), <0.0001  2.7 (2.1; 3.3), <0.0001  -16.2 (-23.5; -8.2), 0.0001  -8.5 (-10.4; -6.6), <0.0001 |
|  |  |  |
| Model 2  **CAC**  **Low GRS Quartile (Q1)**  **Medium GRS Quartile (Q2, Q3)**  **High GRS Quartile (Q4)**  Intercept  Age (years)  Sex  log(CACb+1)  Diabetes  BMI  Systolic blood pressure  LDL-cholesterol  HDL-cholesterol  Social economic status  Antihypertensive medication  Lipid-lowering medication  Past smoker  Current smoker | 719 (24.9)  1544 (53.5)  622 (21.6) | **Reference**  **8.3 (-2.3; 20.1), 0.13**  **20.8 (6.5; 37.0), 0.003**  -65.8 (-81.3; -37.5), 0.0005  2.5 (1.8; 3.1), <0.0001  -12.1 (-21.2; -2.0), 0.02  -10.3 (-12.2; -8.3), <0.0001  42.4 (23.7; 63.9),<0.0001  -3.4 (-4.5; -2.3), <0.0001  0.5 (0.3; 0.8), 0.0001  0.2 (0.1; 0.3), 0.03  -0.1 (-0.4; 0.2), 0.42  -7.4 (-16.0; 2.1), 0.13  13.6 (2.8; 25.5), 0.01  17.5 (1.2; 36.5), 0.03  10.1 (-0.5;21.8), 0.06  33.5 (18.7; 50.1), <0.0001 |

GRS: genetic risk score, CAD: coronary artery disease, CAC: coronary artery calcification, CI: confidence interval, medium GRS quartile consists of Q2 and Q3 and high GRS quartile consist of Q4. The association between the genetic risk scores and outcome was carried out using linear regression in SAS. Model 1: adjusted for age, sex and log(CAC_b_+1). Model 2: adjusted for age, sex, log(CAC_b_+1) and coronary artery disease risk factors (type 2 diabetes, body mass index, socio economic status, systolic blood pressure, smoking, antihypertensive medication, lipid lowering medication, LDL-cholesterol, HDL-cholesterol).

**Table S6(b)**. Association of the genetic risk score quartiles with the 5-year progression of coronary artery calcification

|  | N (%) | Percent change in (CAC+1), (95%CI), p-value |
| --- | --- | --- |
| Model 1 |  |  |
| **CAD**  **Low GRS Quartile (Q1)**  **Medium GRS Quartile (Q2, Q3)**  **High GRS Quartile (Q4)**  Intercept  Age (years)  Sex  log(CACb+1) | 775 (25.0)  1548 (50.0)  774 (25.0) | **Reference**  **4.6 (-4.9; 15.0), 0.35**  **21.2 (8.6; 35.3), 0.0006**  -46.2 (-61.4; -25.0), 0.0003  2.6 (2.0; 3.2), <0.0001  -18.1 (-24.7; -10.9), <0.0001  -5.2 (-7.0;-3.4), <0.0001 |
| Model 2  **CAD**  **Low GRS Quartile (Q1)**  **Medium GRS Quartile (Q2, Q3)**  **High GRS Quartile (Q4)**  Intercept  Age (years)  Sex  log(CACb+1)  Diabetes  BMI  Systolic blood pressure  LDL-cholesterol  HDL-cholesterol  Social economic status  Antihypertensive medication  Lipid-lowering medication  Past smoker  Current smoker | 729 (25.3)  1440 (49.9)  716 (24.8) | **Reference**  **4.6 (-5.0; 15.2), 0.36**  **19.3 (6.6; 33.5), 0.002**  -57.4 (-75.7; -25.5), 0.003  2.4 (1.8; 3.0), <0.0001  -13.4 (-21.8; -4.2), 0.005  -6.9 (-8.8; -5), <0.0001  34.4 (18; 53.1), <0.0001  -2.5 (-3.5; -1.5), <0.0001  0.5 (0.3; 0.7), <0.0001  0.2 (0.1; 0.3), 0.001  -0.1 (-0.4; 0.2), 0.38  -7.7(-15.6; 1), 0.08  11.9 (2.0; 22.7), 0.02  15.3 (0.4; 32.4), 0.04  8.8 (-0.9; 19.5), 0.08  34.9 (21.0; 50.3), <0.0001 |

GRS: genetic risk score, CAD: coronary artery disease, CI: confidence interval, medium GRS quartile consists of Q2 and Q3 and high GRS quartile consist of Q4. The association between the genetic risk scores and outcome was carried out using linear regression in SAS. Model 1: adjusted for age, sex and log(CAC_b_+1). Model 2: adjusted for age, sex, log(CAC_b_+1) and CAD risk factors (type 2 diabetes, body mass index, socio economic status, systolic blood pressure, smoking, antihypertensive medication, lipid lowering medication, LDL-cholesterol, HDL-cholesterol).
